# Transient regulation of focal adhesion via Tensin3 is required for nascent oligodendrocyte differentiation

**DOI:** 10.7554/eLife.80273

**Published:** 2022-10-10

**Authors:** Emeric Merour, Hatem Hmidan, Corentine Marie, Pierre-Henri Helou, Haiyang Lu, Antoine Potel, Jean-Baptiste Hure, Adrien Clavairoly, Yi Ping Shih, Salman Goudarzi, Sebastien Dussaud, Philippe Ravassard, Sassan Hafizi, Su Hao Lo, Bassem A Hassan, Carlos Parras

**Affiliations:** 1 Paris Brain Institute, Sorbonne Université, Inserm U1127, CNRS UMR 7225, Hôpital Pitié‐Salpêtrière Paris France; 2 Department of Biochemistry and Molecular Medicine, University of California, Davis Davis United States; 3 School of Pharmacy and Biomedical Sciences, University of Portsmouth Portsmouth United Kingdom; Max Planck Institute of Experimental Medicine Germany; University of Hong Kong Hong Kong

**Keywords:** oligodendrocyte differentiation, tensin, oligodendroglial survival, immature oligodedrocyte marker, Human, Mouse

## Abstract

The differentiation of oligodendroglia from oligodendrocyte precursor cells (OPCs) to complex and extensive myelinating oligodendrocytes (OLs) is a multistep process that involves large-scale morphological changes with significant strain on the cytoskeleton. While key chromatin and transcriptional regulators of differentiation have been identified, their target genes responsible for the morphological changes occurring during OL myelination are still largely unknown. Here, we show that the regulator of focal adhesion, Tensin3 (Tns3), is a direct target gene of Olig2, Chd7, and Chd8, transcriptional regulators of OL differentiation. Tns3 is transiently upregulated and localized to cell processes of immature OLs, together with integrin-β1, a key mediator of survival at this transient stage. Constitutive *Tns3* loss of function leads to reduced viability in mouse and humans, with surviving knockout mice still expressing Tns3 in oligodendroglia. Acute deletion of *Tns3* in vivo, either in postnatal neural stem cells (NSCs) or in OPCs, leads to a twofold reduction in OL numbers. We find that the transient upregulation of Tns3 is required to protect differentiating OPCs and immature OLs from cell death by preventing the upregulation of p53, a key regulator of apoptosis. Altogether, our findings reveal a specific time window during which transcriptional upregulation of Tns3 in immature OLs is required for OL differentiation likely by mediating integrin-β1 survival signaling to the actin cytoskeleton as OL undergo the large morphological changes required for their terminal differentiation.

## Introduction

Oligodendrocyte (OL) lineage cells, mainly constituted by oligodendrocyte precursor cells (OPCs) and OLs, play key roles during brain development and neuronal support by allowing saltatory conduction of myelinated axons and metabolically supporting these axons with lactate or glucose shuttling through the myelin sheath (Fünfschilling et al., 2012; Lee et al., 2012; Meyer et al., 2018). Accumulating evidence also indicates their fundamental contribution to different aspects of adaptive myelination, a type of brain plasticity (Mount and Monje, 2017; Yang et al., 2020), shown by the requirement of new oligodendrogenesis for proper learning and memory in motor, spatial, and fear-conditioning learning paradigms (McKenzie et al., 2014; Xiao et al., 2016; Steadman et al., 2019; Pan et al., 2020; Wang et al., 2020; Xin and Chan, 2020). Furthermore, oligodendroglia and myelin pathologies have been recently linked not only to the development of glioma (Liu et al., 2011) but to developmental (Castelijns et al., 2020; Phan et al., 2020), neurodegenerative (Grubman et al., 2019; Bryois et al., 2020), and psychiatric (Nott et al., 2019) diseases.

Unlike most precursor cells, OPCs constitute a stable population of the postnatal and adult central nervous system (CNS) (Ffrench-Constant and Raff, 1986; Suzuki and Goldman, 2003). Therefore, OPCs need to keep a tight balance between proliferation, survival, and differentiation. This balance is crucial to maintain the OPC pool while contributing to myelin plasticity in adult life and to remyelination in diseases such as multiple sclerosis (MS). Demyelinated MS plaques can be normally repaired in early stages of the disease, presumably by endogenous OPCs, but this repair process becomes increasingly inefficient with aging, when OPC differentiation seems to be partially impaired (Chang et al., 2002; Compston and Coles, 2002; Neumann et al., 2019). Therefore, understanding the mechanisms involved in OPC differentiation is critical to foster successful (re)myelination in myelin pathologies.

A large diversity of extrinsic signals, including those mediated by integrin signaling (reviewed in Bergles and Richardson, 2016), as well as many intrinsic factors, including transcription factors (TFs) and chromatin remodelers (reviewed in Emery and Lu, 2015; Parras et al., 2020), are involved in OPC proliferation, survival, and differentiation. However, the mechanisms for how these signals balance OPC behavior is largely unknown. OPC differentiation requires profound changes in chromatin and gene expression (Emery and Lu, 2015; Küspert and Wegner, 2016; Wheeler and Fuss, 2016). TFs, such as Olig2, Sox10, Nkx2.2, or Ascl1, are key regulators of OL differentiation by directly controlling the transcription of genes implicated in this process (Qi et al., 2001; Stolt et al., 2002; Nakatani et al., 2013; Yu et al., 2013), but being already expressed at the OPC stage, it is still unclear how these TFs control the induction of differentiation. A growing body of evidence suggests that some of these TFs work together with chromatin remodeling factors during transcriptional initiation/elongation to drive robust transcription (Zaret and Mango, 2016). Accordingly, Olig2 and Sox10 TFs have been shown to cooperate with chromatin remodelers such as Brg1 (Yu et al., 2013), Chd7 (He et al., 2016; Marie et al., 2018), Chd8 (Marie et al., 2018; Zhao et al., 2018), and EP400 (Elsesser et al., 2019), to promote the expression of OL differentiation genes. To improve our understanding of the mechanisms of OL differentiation, we searched for novel common targets of these key regulators by generating and analyzing the common binding profiles of Olig2, Chd7, and Chd8 in gene regulatory elements of differentiating oligodendroglia. We identified *Tns3*, coding for the focal adhesion protein Tensin3, as one such target and showed that it is expressed in immature OLs (iOLs) during myelination and remyelination, thus constituting a marker for this transient oligodendroglial stage. Using different genetic strategies to induce *Tns3* loss-of-function mutations in vivo, we describe the function of a Tensin family member in the CNS, demonstrating that Tns3 is required for OL differentiation in the postnatal mouse brain, at least in part by mediating integrin-β1 signaling, essential for survival of differentiating oligodendroglia (Colognato et al., 2002; Benninger et al., 2006).

## Results

### *Tns3* is a direct target gene of key regulators of oligodendrocyte differentiation

**Figure 1.**
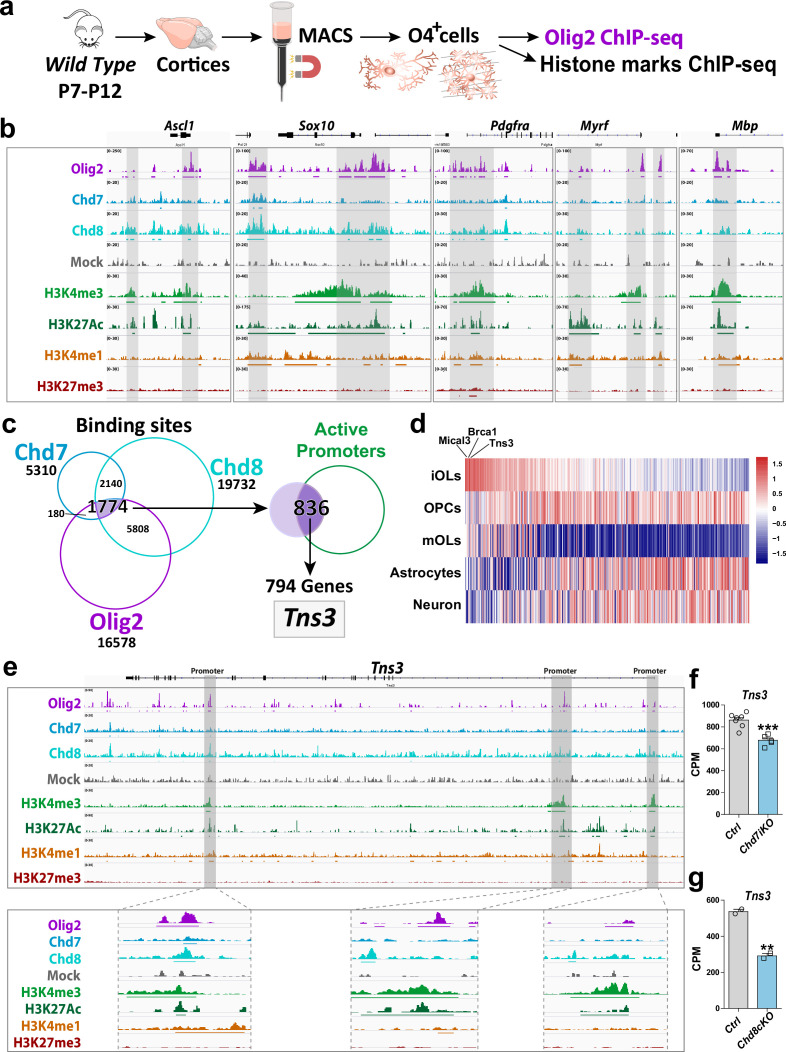
*Tns3* is a target gene of Olig2 and Chd7/8 regulators of oligodendrocyte differentiation. (**a**) Scheme representing MACSorting of O4^+^ cells from wild-type cortices followed by ChIP-seq for Olig2 and histone marks (H3K4me3, H3K27Ac, H3K4me1, and H3K27me3). (**b**) Tracks from IGV browser of *Ascl1, Sox10, Pdgfra, Myrf,* and *Mbp* gene regions depicting ChIP-seq data in O4^+^ cells (OPCs/OLs) for transcription factor Olig2, chromatin remodelers Chd7 and Chd8, and epigenetic marks (H3K4me3, H3K27ac, H3K4me1, and H3K27me3). Mock (control IgG) shows no peaks in the regions of interest. Lines present below peaks indicate statistical significance (by peak calling). (**c**) Strategy used to identify *Tns3* as a gene target of Olig2, Chd7, and Chd8, potentially involved in oligodendrogenesis. Left: Venn diagrams depicting the overlap of binding peaks between Chd7 (blue), Chd8 (cyan), and Olig2 (purple) in O4^+^ cells. Right: Venn diagram showing that 836 (47%) of the 1774 common regions have marks of active promoters, corresponding to 794 genes, including *Tns3*. (**d**) Heatmap representing the expression of the 794 genes in immature oligodendrocytes (iOLs) compared to oligodendrocyte precursor cells (OPCs), myelinating oligodendrocytes (mOLs), astrocytes, and neurons. *Tns3* is the third most specific (data from Zhang et al., 2014). (**e**) Tracks from IGV browser of *Tns3* gene region depicturing ChIP-seq data in O4^+^ cells (OPCs/OLs) for transcription factor Olig2 and epigenetic marks (H3K4me3, H3K27ac, H3K4me1, and H3K27me3), zooming in Tns3 alternative promoters. Mock (control IgG) shows no peaks in the regions of interest. Horizontal lines present below peaks indicate statistical significance (peak calling). (**f, g**) Barplots showing *Tns3* transcript count per million (CPM) in O4^+^ cells upon tamoxifen-induced *Chd7* deletion (*Chd7iKO*), (**f**) or *Chd8* deletion (*Chd8cKO*), (**g**) compared to control (Ctrl). Statistics done using edgeR suite.

To find new factors involved in OL differentiation, we screened for target genes of Olig2, Chd7, and Chd8, key regulators of oligodendrogenesis (Lu et al., 2000; Lu et al., 2002; Yu et al., 2013; He et al., 2016; Marie et al., 2018; Zhao et al., 2018; Parras et al., 2020). We generated and compared the genome-wide binding profiles for these factors in acutely purified oligodendroglial cells from postnatal mouse brain cortices by magnetic cell sorting (MACS) of O4^+^ cells (Marie et al., 2018). MACS-purified cells, composed of 80% PDGFRα^+^ OPCs and 20% of Nkx2.2^+^/CC1^+^ iOLs, were subjected to chromatin immunoprecipitation followed by sequencing (ChIP-seq) for Olig2 and histone modifications marking the transcription activity of gene regulatory elements (H3K4me3, H3K4me1, H3K27me3, and H3K27ac; Figure 1a). The profile of activity histone marks at Olig2-binding sites indicated that Olig2 binds promoters (60%) and enhancers (40%) with either active or more poised/repressive states (Figure 1—figure supplement 1a–f), supporting the suggested pioneer function of Olig2 in oligodendrogenesis (Yu et al., 2013). Among the 16,578 chromatin sites bound by Olig2 corresponding to 8672 genes (Figure 1—figure supplement 1d), there were key regulators of OL differentiation, including *Ascl1, Sox10, Myrf, Chd8,* and *Smarca4/Brg1* (Figure 1b; Supplementary file 1). Combining Olig2 with Chd7 and Chd8 binding profiles, which we previously generated using the same protocol (Marie et al., 2018), we found 1774 chromatin sites commonly bound by the three regulators, with half of them (47% and 832 peaks) corresponding to active promoters (H3K4me3/H3K27ac marks) of 654 protein-coding genes (Figure 1c, Supplementary file 1). Among these genes, *Tns3* coding for Tensin3, a focal adhesion protein deregulated in certain cancers (Martuszewska et al., 2009), showed the highest expression levels in iOLs relative to other brain cell types (Zhang et al., 2014; Figure 1d). Indeed, Olig2, Chd7, and Chd8 commonly bound three putative promoters of *Tns3* having active transcription marks in purified oligodendroglia (H3K27ac/H3K24me3; Figure 1e). To directly assess whether *Tns3* expression requires the activity of these key regulators, we interrogated the transcriptomes of these oligodendroglial cells purified from *Chd7iKO* (*Pdgfra-CreER^T^; Chd7^flox/flox^*), *Chd8cKO (Olig1^Cre^; Chd8 ^flox/flox^*), and their respective control cortices (Marie et al., 2018; Zhao et al., 2018). *Tns3* transcripts were largely downregulated upon acute deletion of these factors in postnatal OPCs/iOLs (Figure 1f and g), indicating that *Tns3* expression in OPCs/iOLs is directly controlled by Chd7 and Chd8 chromatin remodelers, key regulators of OL differentiation.

**Figure 1—figure supplement 1.**
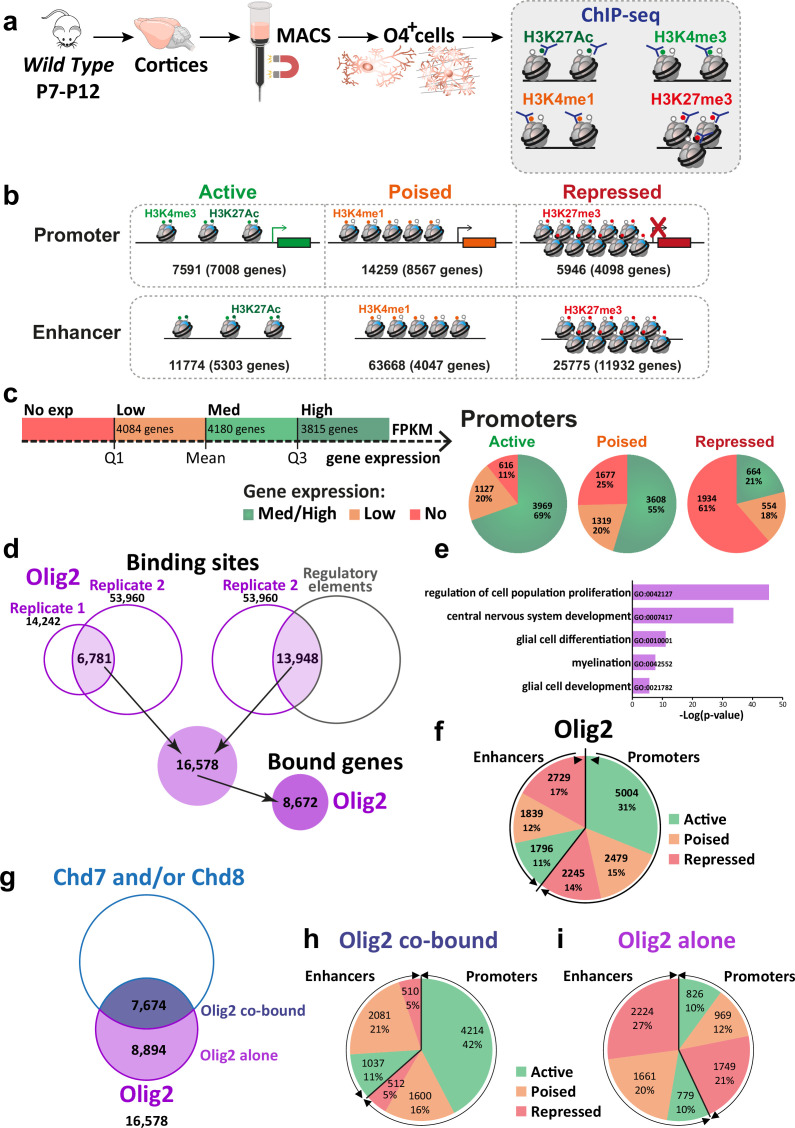
*Tns3* is a target gene of Olig2 and Chd7/8 regulators of oligodendrocyte differentiation. (**a**) Scheme representing MACSorting of O4^+^ cells from wild-type cortices followed by ChIP-seq for histone marks H3K4me3, H3K27Ac, H3K4me1, and H3K27me3. (**b**) Schematic representing gene regulatory elements in O4^+^ cells as active, poised, and repressed, according to their histone marks. (**c**) Left: diagram representing the classification of gene expression (data from Marie et al., 2018). Right: pie chart representing percentage of medium-to-high, low or not expressed genes having an active, poised, and repressed promoter in O4^+^ cells. Note the high percentage of medium-to-high expressed genes having active marks in their promoter and the high percentage of not expressed genes having repression marks in their promoter. (**d**) Venn diagram representing the method used to determine Olig2 binding peaks. As the quality of the peaks in replicate 2 was better than in replicate 1, we considered peaks common in both replicate and the peaks of replicate 2 that were present in regulatory elements (regions with histone marks). 16,578 peaks were found corresponding to 8672 genes bound by Olig2. (**e**) Gene Ontology category found enriched in Olig2-bound genes. (**f**) Pie chart representing the number and repartition of Olig2-binding sites in regulatory regions with different activity histone marks. Note that Olig2 binds in both promoters (60%) and enhancers (40%), and that it is found in either active, poised, or repressed regions. (**g**) Venn diagram representing the amount of Olig2-binding sites that are also bound by Chd7 and/or Chd8 (7674; Olig2 co-bound) or not (8894; Olig2 alone). (**h, i**) Pie chart representing the number and repartition of Olig2 co-bound (**h**) or alone (**i**) sites in regulatory regions with different activity histone marks. Note that 42% Olig2 co-bound sites are in active promoters, whereas 48% of Olig2 alone sites are in repressed promoter or enhancers.

**Figure 1—figure supplement 2.**
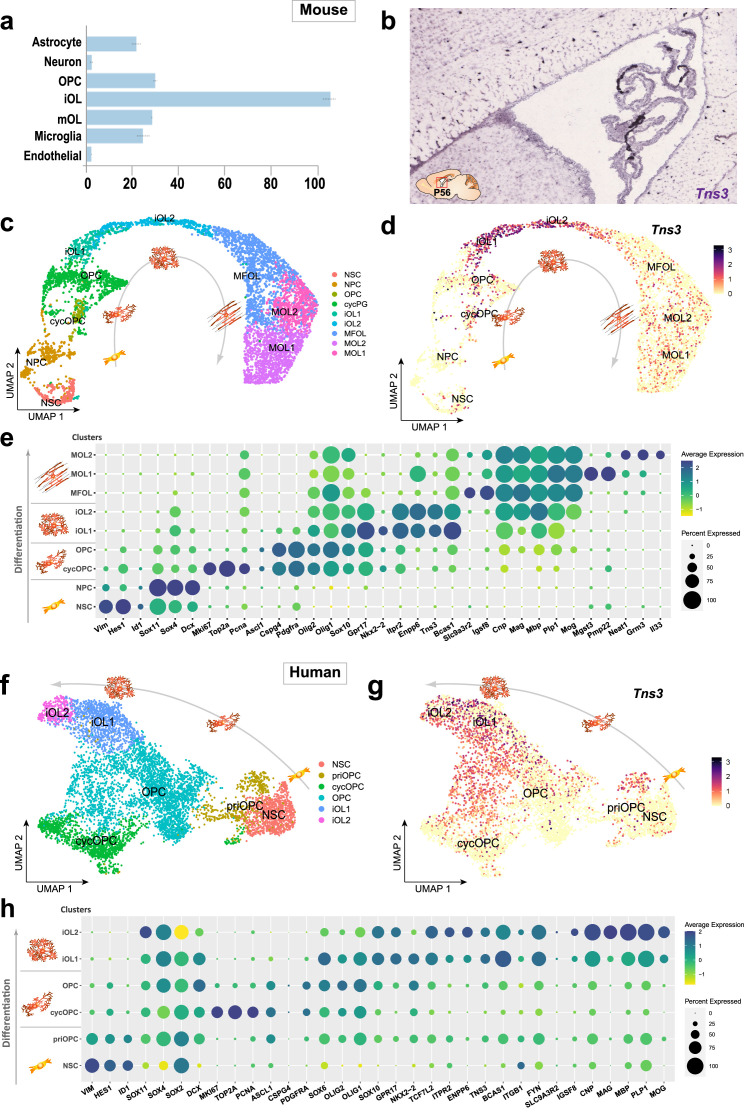
Strong expression of *Tns3* transcripts in mouse and human immature oligodendrocytes. (**a**) Barplot of Tns3 mRNA transcript levels (FKPM) in postnatal brain cell types (Zhang et al., 2014; brainrnaseq.org). (**b**) In situ hybridization in sagittal section of the adult (P56) mouse brain at the level of the lateral ventricles showing *Tns3* transcript expression in sparse white matter cells (Allen Brain Atlas, portal.brain-map.org). (**c**) Uniform manifold approximation and projection (UMAP) representation of neural progenitors and oligodendroglial cells extracted and integrated using Seurat from scRNA-seq datasets (Marques et al., 2016), representing different clusters corresponding to different oligodendroglial stages of neural stem cells (NSCs) to myelinating oligodendrocytes (mOLs). (**d**) Feature plot representing relative expression levels of *Tns3* transcript. (**e**) Dot plot representing the transcript expression of key markers for each cell stage/subtype and key oligodendroglial factors in the different clusters showing the predominant expression of *Tns3* in iOL1s and iOL2s clusters, similar to *Enpp6* and *Itpr2*. NPC, neural progenitor cells; cycOPC, cycling OPC; MFOL, myelin-forming OL; MOL1/2, myelinating OL 1/2. (**f–h**) *TNS3* expression in human oligodendroglial cells differentiated from iPSCs by scRNA-seq (Chamling et al., 2021), showing *TNS3* high levels of expression in immature oligodendrocytes. (**f**) Uniform manifold approximation and projection (UMAP) representation showing six clusters (NSC, cycOPC, OPCs, and two iOL stages, iOL1 and iOL2, similar to mouse oligodendroglia). (**g**) Feature plot showing *TNS3* expression levels. (**h**) Dot plots showing *TNS3* expression pattern together with key markers of each cluster and regulators of oligodendrogenesis.

**Figure 1—figure supplement 3.**
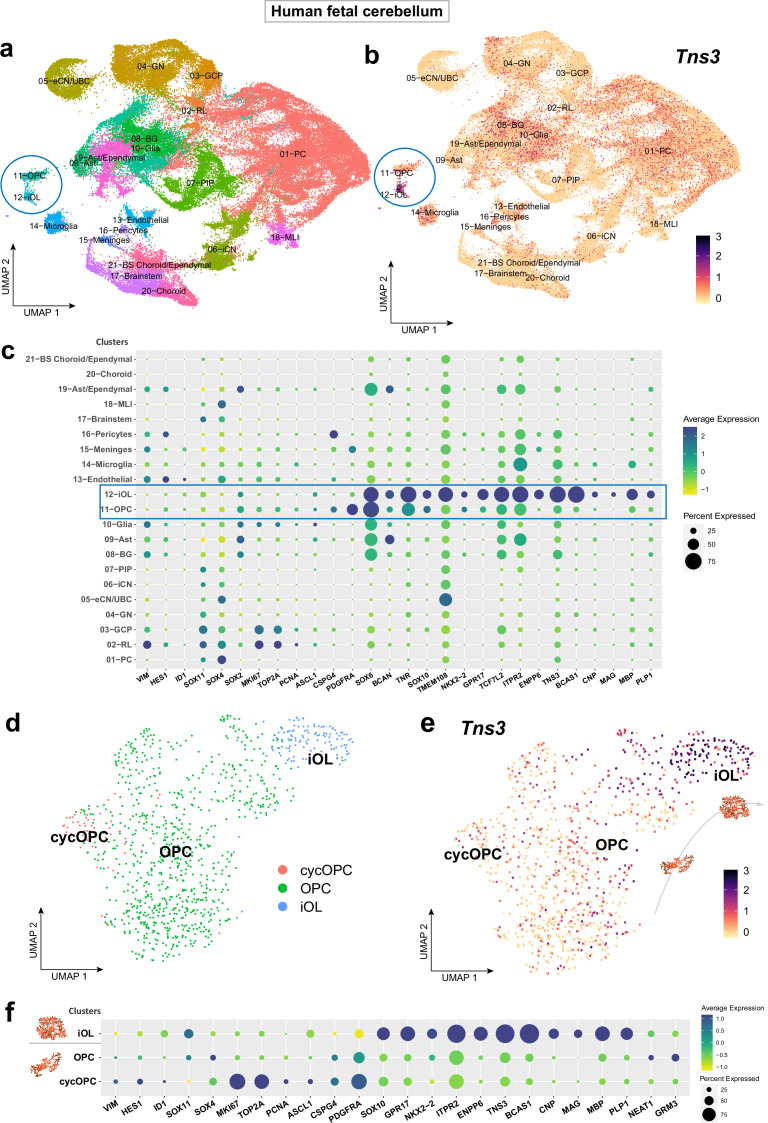
Strong expression of *Tns3* transcripts in immature oligodendrocytes (iOLs) of human fetal cerebellum. (**a**) Uniform manifold approximation and projection (UMAP) representation of different cell-type cluster found in human fetal cerebellum (GW 9–22), including oligodendrocyte precursor cells (OPCs) and iOLs clusters (blue circle). (**b**) Feature plot representing relative expression levels of *Tns3* transcript, presenting high levels in iOL cluster cells (**c**) Dot plot representing the transcript expression of key markers for each cell stage/subtype and key oligodendroglial factors in the different clusters showing the predominant expression of *TNS3* in iOLs clusters (OPC and iOL clusters highlighted by blue rectangle). (**d**) UMAP representation of new clustering of oligodendroglial cells (11-OPC and 12-iOL clusters) showing OPCs, cycling (cyc) OPCs, and iOLs. (**e**) Feature plot representing relative expression levels of *TNS3* transcript showing highest levels in iOL cells. (**f**) Dot plot showing *TNS3* expression pattern together with key markers of each cluster and regulators of oligodendrogenesis, showing similar expression pattern of *TNS3* and other suggested iOL markers (including *ITPR2, ENPP6,* and *BCAS1*).

### *Tns3* transcripts are highly expressed in mouse and human immature oligodendrocytes

We then investigated *Tns3* expression pattern in the brain. High expression levels of *Tns3* transcripts in iOLs, compared to its low expression in other cells of the postnatal mouse brain detected by bulk transcriptomics (Figure 1—figure supplement 2a; Zhang et al., 2014), were paralleled by the sparse labeling with *Tns3* probes enriched in the white matter of the postnatal and adult brain detected by in situ hybridization (Figure 1—figure supplement 2b; Allen Brain Atlas, https://portal.brain-map.org/). By harnessing single-cell transcriptomics (scRNA-seq), we sought to create an integrative gene profiling for OL lineage cells by bioinformatics integration and analyses of OL lineage cells at embryonic, postnatal, and adult stages (Marques et al., 2016). We thus integrated these datasets using Seurat (Stuart et al., 2019) and selected 5516 progenitor and oligodendroglial cells. Unsupervised clustering and visualization of cells in two dimensions with uniform manifold approximation and projection (UMAP) identified nine different clusters following a differentiation trajectory. Based on known cell-subtype-specific markers (Figure 1—figure supplement 2e and Supplementary file 1), we could identify these clusters as (Figure 1—figure supplement 2c and e): (1) two types of neural stem/progenitor cells, which we named NSCs and NPCs according to their expression of stem cell (*Vim, Hes1, Id1*) and neural progenitor (*Sox11, Sox4, Dcx*) markers; (2) OPCs expressing their known markers (*Pdgfra, Cspg4, Ascl1*) and cycling OPCs also enriched in cell cycle markers (*Mki67, Pcna, Top2*); (3) two stages of iOLs, both expressing the recently proposed markers *Itpr2* and *Enpp6* (Marques et al., 2016; Xiao et al., 2016), and which are split by the expression of *Nkx2-2* (iOL1 being *Nkx2-2*^+^ and iOL2 being *Nkx2-2*^-^), in agreement with our previous characterization by immunofluorescence (Nakatani et al., 2013; Marie et al., 2018); (4) myelin-forming oligodendrocytes (MFOLs), enriched in markers such as *Slc9a3r2* and *Igsf8*; and (5) two clusters of myelinating OLs, which we named MOL1 and MOL2, expressing transcripts of myelin proteins (*Cnp, Mag, Mbp*, *Plp1*, *Mog*) and some specific markers of each cluster, including *Mgst3, Pmp22* for MOL1 (corresponding to MOL1/2/3/4 clusters of Marques et al., 2016), and *Neat1*, *Grm3*, *Il33* for MOL2 (corresponding to MOL5/6 clusters of Marques et al., 2016). Interestingly, *Tns3* transcripts were strongly expressed in both iOL1 and iOL2 clusters (Figure 1—figure supplement 2d and e), similar to the recently proposed iOL markers *Itpr2* and *Enpp6* (Figure 1—figure supplement 2e), and downregulated in mature/terminally differentiated OLs, indicating that high levels of *Tns3* expression are specific to iOLs. We finally assessed whether *Tns3* expression pattern was conserved in human oligodendroglia pursuing a similar bioinformatics analysis using single-cell transcriptomes from human oligodendroglia differentiated from embryonic stem cells (Chamling et al., 2021). Upon integration with Seurat and identification of cluster cell types using specific markers, we selected 7690 progenitor and oligodendroglial cells that corresponded to six main cluster cell types following a differentiation trajectory from neural cells (NSCs) up to iOLs (iOL1 and iOL2), as depicted by UMAP representation (Figure 1—figure supplement 2f and h). Cells expressing high levels of *TNS3* corresponded to iOLs (iOL1 and iOL2 clusters, Figure 1—figure supplement 2g and h). We obtained similar results analyzing a human fetal midterm cerebellum (GW9-GW22) dataset (Aldinger et al., 2021), with high levels of *TNS3* in iOLs similar to other suggested iOL markers such as *ITPR2*, *ENPP6,* and *BCAS1,* indicating a conserved expression pattern of Tns3/TNS3 between mouse and human oligodendrogenesis (Figure 1—figure supplement 3).

### Tns3 protein is enriched in the cytoplasm and processes of immature oligodendrocytes

**Figure 2.**
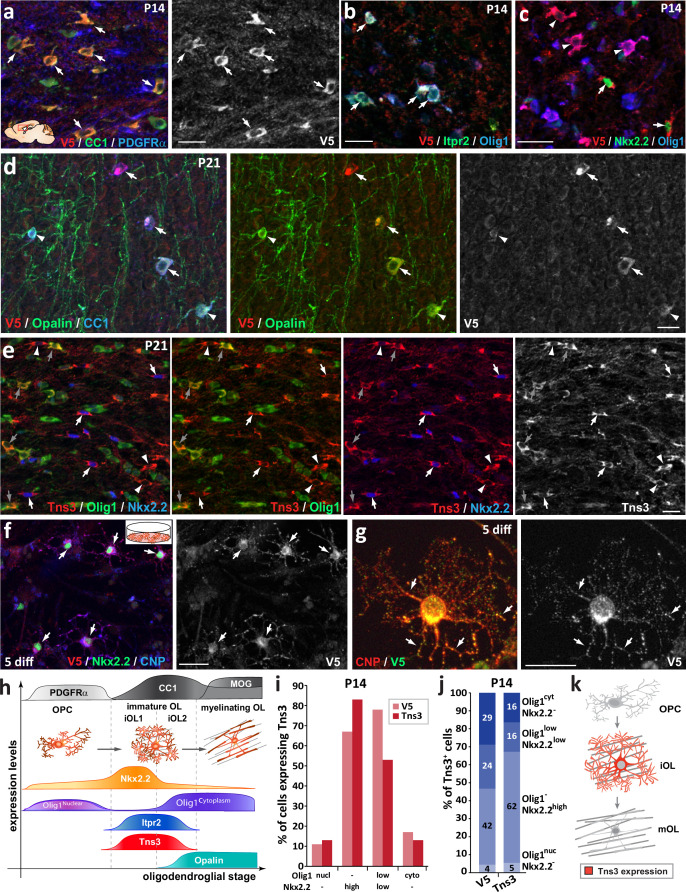
Tns3 protein is detected at high levels in the cytoplasm and main cell processes of immature oligodendrocytes (iOLs) in the postnatal brain. Immunofluorescence in sagittal sections of postnatal brain at the level of the corpus callosum at P14 (**a–c**) and P21 (**d-e**) using V5 and Tns3 antibodies. (**a**) Tns3-V5 is detected at high levels in CC1^+^ OLs (arrows) but not in PDGFRα^+^ oligodendrocyte precursor cells (OPCs). (**b**) Tns3-V5 expression overlaps well with Itpr2 (arrows), with some of them being Olig1^high-cytoplamic^ cells. (**c**) Tns3-V5 overlaps with high levels of Nkx2.2 expression (arrows) and also with Nkx2.2^-^/Olig1^high-cytoplamic^ cells (arrowheads). (**d**) Tns3-V5 expression overlaps with Opalin in iOLs (arrows, CC1^+^ cells with large cytoplasm) but is downregulated in Opalin^+^ myelinating oligodendrocytes (mOLs) (arrowheads, CC1^+^ cells with small cytoplasm and myelin segments). (**e**) Tns3 (Sigma-Ct antibody) is detected at high levels in Nkx2.2^+^/Olig1^-^ early iOL1s (white arrows), in late Nkx2.2^-^/Olig1^-^ iOL1s (white arrowheads), and in Nkx2.2^-^/Olig1^high-cytoplamic^ iOL2s (gray arrows). (**f**) Tns3-V5 expression neural stem cell (NSC) cultures after 5 days in differentiation. Note the Tns3 expression in Nkx2.2^+^/CNP^+^ OLs (arrows). (**g**) Subcellular localization of Tns3 expression in CNP^+^ OLs present in the cytoplasm and in dots distributed along the cell processes, overlapping with CNP signal (arrows). (**h**) Schematic representation of Tns3 expression together with key markers of different oligodendroglial stages summarizing data shown in (**a–e**). (**i**) Histograms representing the percentage of Nkx2.2^-^/Olig1^high-nuclear^, Nkx2.2^high^/Olig1^-^, Nkx2.2^low^/Olig1^low-cytoplamic^ and Nkx2.2^-^/Olig1^high-cytoplamic^ cells expressing Tns3-V5 and Tns3 at P14. (**j**) Histograms representing the percentage of Tns3-V5^+^ and Tns3^+^ cells at P14 that are Nkx2.2^-^/Olig1^high-nuclear^, Nkx2.2^high^/Olig1^-^, Nkx2.2^low^/Olig1^low-cytoplamic^ and Nkx2.2^-^/Olig1^high-cytoplamic^. (**k**) Schematic representation of Tns3 expression and subcellular localization in oligodendroglia. Scale bars: (**a–f**) 20 μm; (**g**) 10 μm.

Given the high expression level of *Tns3* transcripts in iOLs, we characterized the Tns3 protein expression pattern in the postnatal brain using commercial and homemade Tns3-recognizing antibodies. Optimization of immunofluorescence protocols demonstrated signal in CC1^+^ OLs in the postnatal brain with four different antibodies (P24, Figure 2—figure supplement 1). To our surprise, while all antibodies showed signal localized in the cytoplasm and main processes of CC1+ OLs (Figure 2—figure supplement 1a–d), one Tns3-recognizing antibody (Millipore) also presented a strong nuclear signal (Figure 2—figure supplement 1d) never reported for Tns3 localization in other tissues (such as lung, liver, and intestine) (Katz et al., 2007; Nishino et al., 2012; Cao et al., 2015). To better characterize Tns3 protein expression pattern and its subcellular localization, we generated a knock-in mice tagging the Tns3 C-terminal side with a V5-tag (*Tns3^Tns3^*^-V5^ mice) by microinjecting mouse zygotes with a single-strand oligodeoxynucleotide (ssODN) containing V5 sequence together with Cas9 protein and a gRNA targeting the stop codon region of *Tns3* (‘Materials and methods’; Figure 2—figure supplement 2a–c). We first verified by immunofluorescence that Tns3-V5 protein in *Tns3^Tns3^*^-V5^ mice presented the expression pattern reported for Tns3 in the lung and the kidney (Figure 2—figure supplement 2d and e). We then characterized Tns3 protein expression in oligodendroglia using V5 antibodies, finding that Tns3 protein can be detected at high levels in the cytoplasm and main processes of CC1^+^ iOLs but not in their nuclei (Figure 2a). Using an antibody recognizing Itpr2, a suggested iOL marker (Marques et al., 2016), we saw that Tns3 largely overlapped with Itpr2 (Figure 2b). Using Nkx2.2 and Olig1^cytoplamic^ expression distinguishing iOL1 and iOL2, respectively, we found high levels of Tns3 in iOL1s (Nkx2.2^+^/Olig1^-^ cells) and a fraction of iOL2s (Nkx2.2^-^/Olig1^cytoplamic^ cells; Figure 2c, i and j), suggesting that Tns3 protein expression peaks in early iOLs. Comparison with Opalin protein localized in the cell body, processes, and myelin segments of OLs showed that Tns3 levels decreased with increasing levels of Opalin, with Tns3-V5 levels undetectable in myelinating OLs (i.e., Opalin^+^/CC1^+^ cells presenting myelinated segments; Figure 2d, arrowheads). We then performed Western blot analysis with anti-V5 antibodies in purified O4^+^ cells from P7, P14, and P21 *Tns3^Tns3^*^-V5^ mouse brains to assess their specificity to recognize Tns3-V5, knowing that two Tns3 isoforms can be detected at the transcript level in the human brain (Figure 2—figure supplement 2f, GTEX project, gtexportal.org/home/gene/TNS3). Indeed, we could detect both the full-length (1450 aa, 155 kDa) and the Tns3 short (C-term, 61 kDa) isoforms in O4^+^ cells from brains at P7 and P14 stages having many iOLs, but not at P21 having mainly mOLs (Figure 2—figure supplement 2g and h), thus validating the specificity of the anti-V5 antibodies in recognizing Tns3 protein. We eventually found a Tns3 antibody also recognizing the C-terminal of Tns3 protein (Sigma Ct) that upon optimized immunofluorescence labeling confirmed the Tns3 expression pattern seen with the V5 antibodies. In combination with Nkx2.2 and Olig1 immunofluorescence, it showed that Tns3 is strongly detected in the cytoplasm and main cellular processes of all iOL1s, defined as Nkx2.2^high^/Olig1^-^ cells having a round nucleus and small cytoplasm (Figure 2e, i and j, white arrows), and it divided iOL2s, defined as Nkx2.2^-^/CC1^high^ cells, into three stages: (1) Tns3^high^/Nkx2.2^-^/Olig1^-^ (Figure 2e, i and j, arrowheads), (2) Tns3^high^/Nkx2.2^-^/Olig1^high-cytoplamic^ (Figure 2e, i and j, gray arrows), and (3) Tns3^-^/Nkx2.2^-^/ Olig1^high-cytoplamic^ (Figure 2e, i and j). A similar Tns3 expression pattern and localization was found in vitro using neonatal neural progenitors’ differentiating cultures, where Tns3 was detected together with CNP myelin protein in the cytoplasm and cell processes of Nkx2.2^high^/CNP^+^ differentiating OLs (Figure 2f and g). Altogether, these results indicate that high but transient levels of Tns3 protein characterize early iOLs (iOL1s and early iOL2s), being localized at their cytoplasm and cell processes (Figure 2h and k).

**Figure 2—figure supplement 1.**
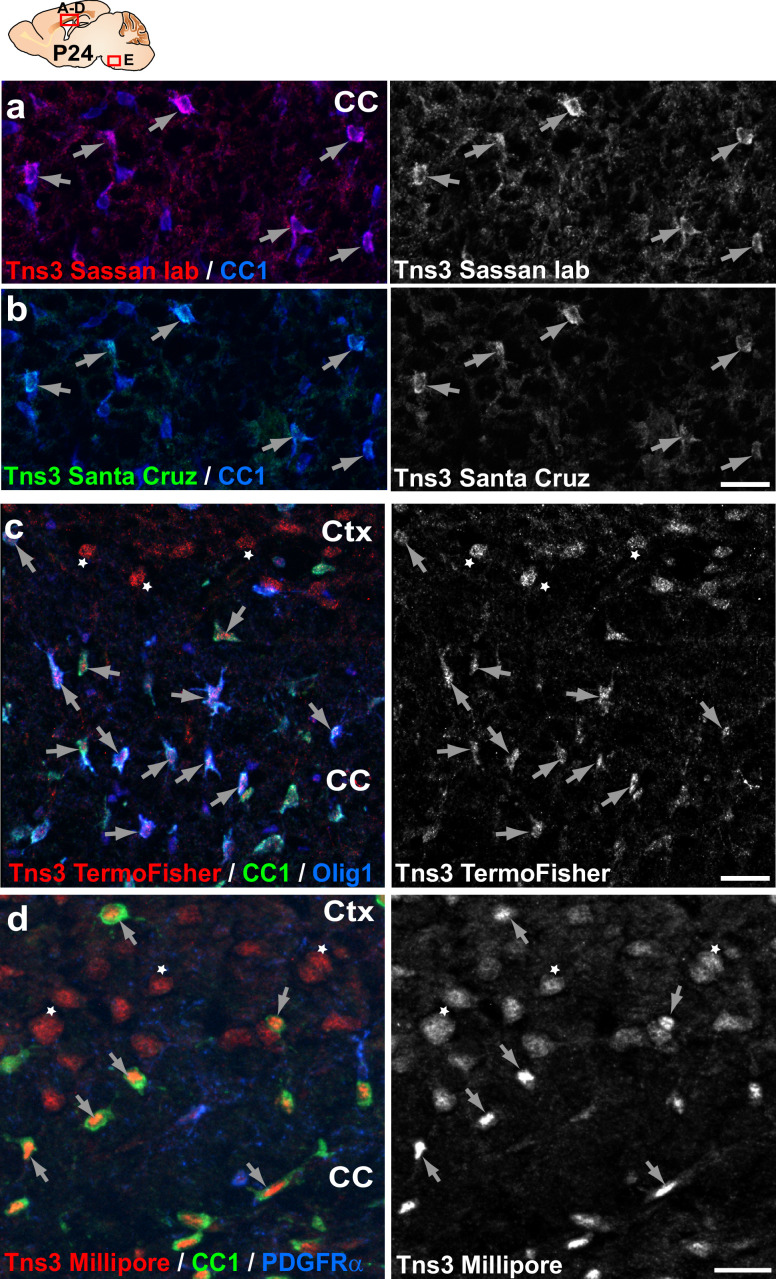
Immunodetection of Tns3 protein in immature oligodendrocytes (OLs) of the postnatal brain. (**a–d**) Tns3 expression in CC1^+^ OLs (arrows) at the level of the corpus callosum (CC) of P24 mice, using a homemade antibody from Sassan Hafizi lab (University of Portsmouth) (**a**), and commercial antibodies from Santa Cruz (**b**), Thermo Fisher (**c**), and Millipore (**d**). Note that Millipore antibody is the only one to show clear nuclear localization in OLs. Thermo Fisher and Millipore antibodies also recognize a nuclear signal in cortical neurons (**c, d**, stars), not found with other Tns3 antibodies. Scale bar, 20 μm.

**Figure 2—figure supplement 2.**
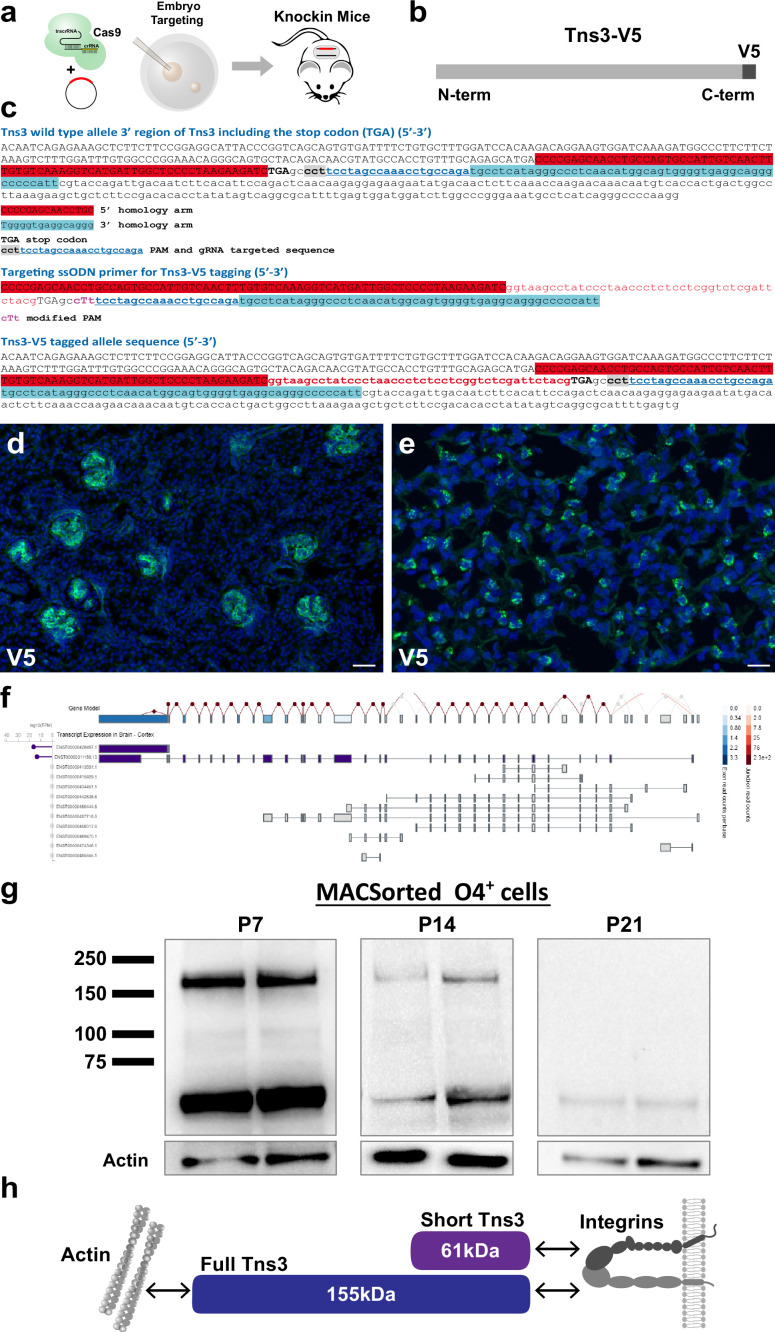
Generation of *Tns3^Tns3-V5^* knock-in mice. (**a**) Scheme of V5 tag knock-in strategy in mice. (**b**) Schematic of Tns3 protein with a V5 tag at the C-terminal. (**c**) Nucleotide sequence of a wild-type mice *Tns3* 3′ region (upper), of the targeting single-strand oligodeoxynucleotide (ssODN) vector (middle), and of the 3′ region of the first Tns3^Tns3-V5^ knock-in mice generated (lower). (**d, e**) Immunostaining using V5 antibody in kidney (**d**) and lung (**e**) of P14 *Tns3^Tns3-V5^* mice. Note the expression of Tns3 in kidney glomeruli and lung alveolar cells (**f**) Schematic of the exons present in the two main *TNS3* transcript isoforms expressed in the human brain (gtexportal.org/home/gene/TNS3). A dark color is associated with higher expression. (**g**) Western blots of V5 antibody in O4^+^ MACSorted cells from P7 oligodendrocyte precursor cells (OPCs)/immature oligodendrocytes (iOLs) (left), P14 iOLs (middle), and P21 OLs (right) of *Tns3^Tns3-V5^* mouse brains. Bottom: actin loading control. Note the presence of the full-length isoform only in OPC/iOLs. (**h**) Schematic of Tns3 protein brain isoforms and their interactions with actin and integrins. Scale bar, 20 μm. Figure 2—figure supplement 2—source data 1.Anti-V5 Western blot followed by anti-actinWestern blot at P7, P14 and P21.

**Figure 2—figure supplement 3.**
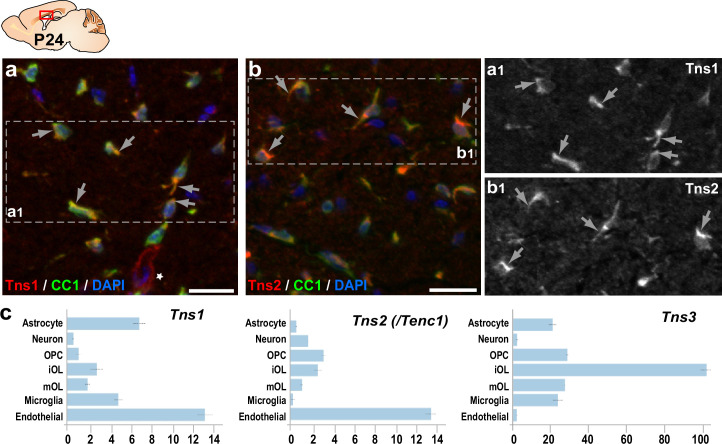
Tns1 and Tns2 proteins are detected at low levels in immature oligodendrocytes (OLs). Immunofluorescence in sagittal section of P24 mouse brain. Tns1 (**a**) and Tns2 (**b**) immunodetection in CC1^+^ OLs of the corpus callosum. Tns1 is detected in the cytoplasm and excluded from OL nucleus (**a_1_**), as is Tns2 (**b_1_**). (**c**) *Tns1*, *Tns2,* and *Tns3* mRNA expression in postnatal brain (brainrnaseq.org). Note that *Tns3* is the main Tensin expressed in oligodendroglia. Scale bar, 20 μm.

**Figure 2—figure supplement 4.**
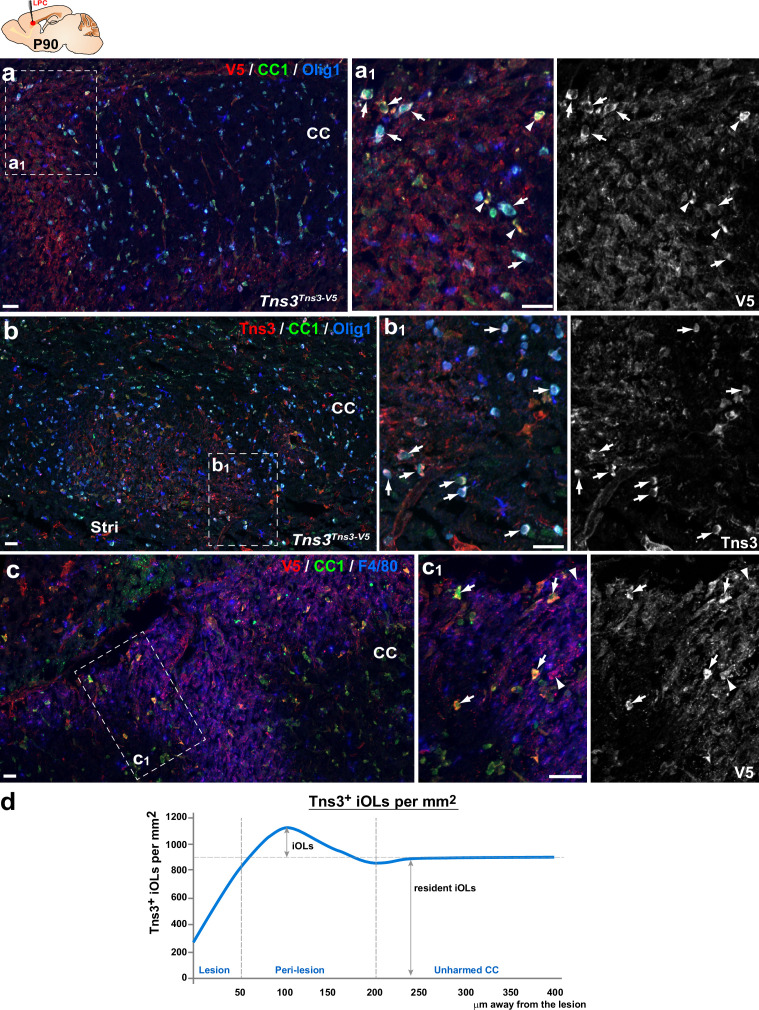
Tns3 is expressed in newly formed oligodendrocytes (OLs) during adult brain remyelination. (**a, b**) Tns3-expressing cells in the corpus callosum (CC) of adult (P90) mice, 7 days after chemically induced demyelination, showing strong Tns3 expression in CC1^+^/Olig1^-^ iOL1s (arrowheads) and CC1^+^/Olig1^cytoplasmic^ iOL2s (arrows) around the lesion using either anti-V5 antibody in *Tns3^Tns3^*^-V5^ mice (**a–a_1_**) or anti-Tns3 antibody in wild-type mice (**b–b_1_**). Note the absence of CC1^+^/Olig1^+^ OLs in the lesion. (**c–c_1_**) Tns3 expression in CC1^high^ iOLs (arrows) around the lesion and in some F4/80^+^ microglia (arrowheads) in the lesion area. (**a_1_), (b_1_**), and (**c_1_**) are higher magnification of corresponding insets (squares). (**d**) Quantification of the Tns3^+^ OLs density each 50 μm distance away from the lesion. Scale bar, 20 μm.

Finally, we investigated whether other Tensin family members were expressed in oligodendroglia, finding that Tns1 and Tns2 but not Tns4 were detectable at low levels in iOLs by immunofluorescence (Figure 2—figure supplement 3a and b), paralleling their low transcription levels compared to *Tns3* (Figure 2—figure supplement 3c; brainrnaseq.org). Therefore, Tns3 appears to be the main Tensin expressed during OL differentiation, suggesting that Tns3 function in iOLs is likely to be evolutionarily selected, and thus of biological importance in oligodendrogenesis.

### Tns3 expression is found in immature oligodendrocytes during remyelination

Given the strong Tns3 expression in iOLs during postnatal myelination, we hypothesized that Tns3 expression could be enriched during remyelination in newly formed OLs contributing to remyelination. To test this hypothesis, we performed lysolecithin (LPC) focal demyelinating lesions in the corpus callosum of adult (P90) *Tns3^Tns3^*^-V5^ and wild-type mice, and assessed for Tns3 expression at the peak of OL differentiation (8 days post-lesion) in this remyelinating model (Nait-Oumesmar et al., 1999). We found that while non-lesioned adult brain regions contained only sparse Tns3^+^ iOLs (CC1^high^/Olig1^cyto-high^ cells), remarkably many Tns3^+^ iOLs were detected in the remyelinating area using both V5 (Figure 2—figure supplement 4a and c, arrows) and Tns3 antibodies (Figure 2—figure supplement 4b, arrows). Quantification of Tns3^+^ cells showed a clear increase in Tns3^+^ iOLs around the lesion borders compared to the corpus callosum far from the lesion area (Figure 2—figure supplement 4d), suggesting that Tns3 expression may be a useful marker of ongoing remyelination and lesion repair. Of note, we could also detect Tns3 expression in some microglia/macrophages in the lesion area using a combination of F4/80 antibodies (Figure 2—figure supplement 4c, arrowheads). Altogether, all these data indicate that Tns3 expression peaks at the onset of OL differentiation, labeling iOLs during both myelination and remyelination.

### In vivo CRISPR-mediated *Tns3* loss of function in neonatal neural stem cells impairs oligodendrocyte differentiation

To explore the role of Tns3 in OL differentiation, we first utilized a *Tns3* gene trap mouse line (*Tns3^βgeo^*; Chiang et al., 2005) and two CRISPR-mediated indel mutation mice presumptively leading to Tns3 constitutive knockout. Analyses of these three mouse lines (Figure 3—figure supplement 1; see ‘Materials and methods’ for details) showed both developmental lethality (in line with loss-of-function variants of TNS3 causing ~80% developmental mortality in the human population; https://gnomad.broadinstitute.org; Figure 3—figure supplement 2; ‘Materials and methods’) and possible genetic compensation in Tns3 expression, making them inappropriate tools to study Tns3 function in oligodendrogenesis.

**Figure 3.**
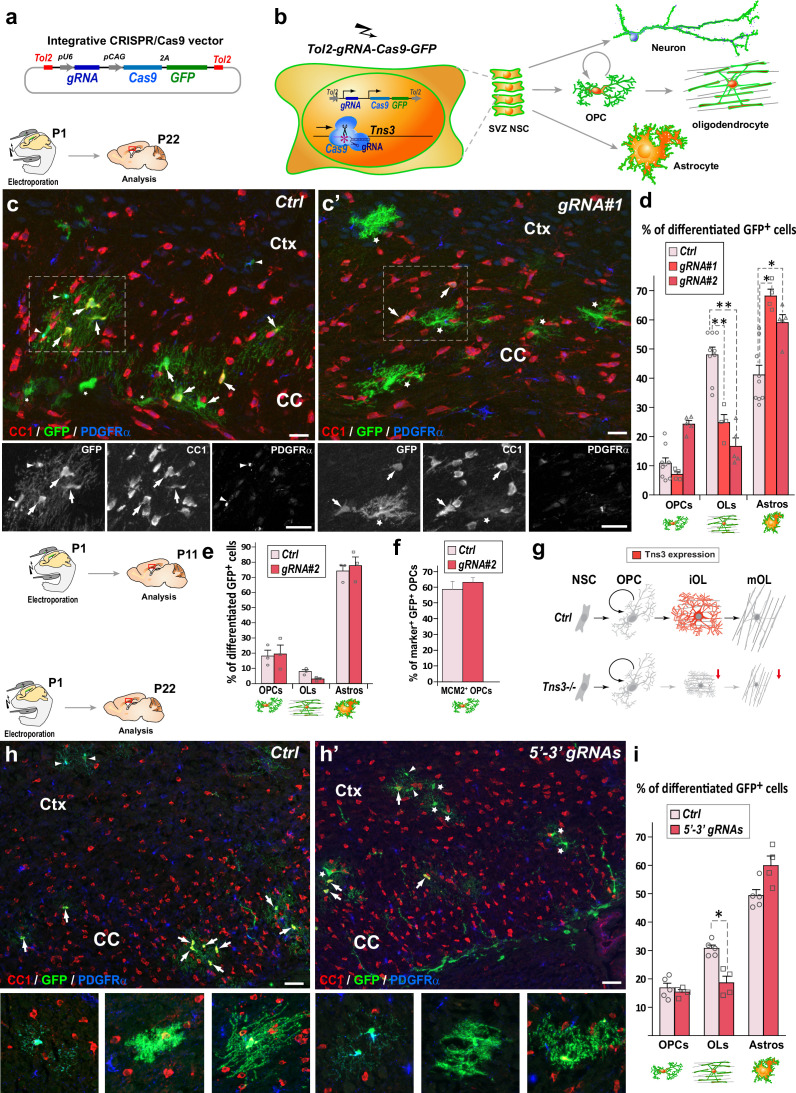
CRISPR-mediated *Tns3* mutation in neural stem cells (NSCs) reduces oligodendrocyte (OL) differentiation in the postnatal brain. (**a**) Schematic of the CRISPR/Cas9 expression vector allowing Tol2-DNA integration driving Cas9 and GFP expression (from polycistronic 2A-mediated cleaved) from CAG promoter and sgRNA expression from U6 promoter. (**b**) Schematic of the dorsal subventricular zone (SVZ) NSC electroporation of CRISPR-plasmid at postnatal day 1 (P1) and traced neural cell-subtype progeny. (**c, c’**) Immunofluorescence of representative P22 sagittal sections of the dorsal telencephalon showing GFP^+^ cells being either PDGFRα^+^ oligodendrocyte precursor cells (OPCs) (arrowheads), CC1^high^ OLs (arrows), or CC1^low^ astrocytes (asterisks) progeny of P1 NSCs electroporated either with *Ctrl* plasmid (**c**) or *Tns3-gRNA#1* plasmid (**c’**). (**d**) Histograms showing the percentage of GFP^+^ glial cell types found in *Ctrl*, *gRNA#1* or *gRNA#2* electroporated brains being PDGFRα^+^-OPCs, CC1^high^-OLs and CC1^low^-astrocytes. Note the twofold reduction of CC1^+^ OLs in *Tns3-gRNA*-transfected brains, as illustrated in (**c’**) compared to (**c**). (**e**) Histograms representing the percentage of GFP^+^ differentiated cells at P11. Note the lack of changes in OPCs, and the incipient reduction in OLs. (**f**) Histograms quantifying the proportion of proliferative (MCM2^+^ cells) GFP^+^ OPCs in electroporated P11 mice brain. (**g**) Schematic of Tns3 expression in mice (upper) and of the effects of *Tns3* CRISPR-mediated deletion (lower). (**h, h’**) Representative P22 sagittal sections of the dorsal telencephalon showing GFP^+^ cells being either PDGFRα^+^ OPCs (arrowheads), CC1^high^ OLs (arrows), or CC1^low^ astrocytes (asterisks) progeny of P1 NSCs electroporated either with *Ctrl* plasmid (**h**) or *Tns3-5′–3′* targeting plasmid (labeled as *5′–3′ gRNAs*) (**h’**). (**i**) Histograms showing the percentage of GFP^+^ glial cell types found in *Ctrl* or 3′–5′ *gRNA* electroporated brains being PDGFRα^+^-OPCs, CC1^high^ OLs, and CC1^low^ astrocytes in the corpus callosum (CC) and cortex (Ctx). Note the twofold reduction of CC1^+^ OLs in *Tns3-5′–3′ gRNA*-transfected brains. Scale bar, 20 μm. Figure 3—source data 1.Subcloning strategy of to generate the Tol2-pCAG-Cas9-2A-GFP plasmid.

Given the tendency of cells to escape the *Tns3* loss of function upon constitutive knockout mutations, we decided to assess *Tns3* requirement during postnatal oligodendrogenesis by inducing in vivo acute *Tns3* deletion in few neural stem cells (NSCs) of the neonatal brain and tracing their cell progeny with a GFP reporter. For this, we combined the postnatal electroporation technique with CRISPR/Cas9 technology. First, we used our previously validated gRNAs targeting *Tns3* at the first coding ATG (exon 6; Figure 3—figure supplement 3; ‘Materials and methods’) inserting them in an integrative CRISPR/Cas9 plasmid also expressing the GFP reporter (Figure 3a), to transfect neonatal NSCs of the dorsal subventricular zone (SVZ), which generate a large number of oligodendroglial cells during the first postnatal weeks (Kessaris et al., 2006; Nakatani et al., 2013), and focused our study on glial cells by quantifying the GFP^+^ progeny of targeted NSCs, outside the SVZ and located in the dorsal telencephalon 3 weeks later (P22, Figure 3b). The fate of GFP^+^ cells was determined by immunodetection of GFP and glial subtype markers (CC1^high^ for OLs, PDGFRα for OPCs, and CC1^low^ and their unique branched morphology for astrocytes). Remarkably, brains electroporated with the CRISPR plasmids targeting *Tns3* had a twofold reduction in GFP^+^ OLs compared to brains electroporated with control plasmids, while GFP^+^ OPCs were found in similar proportions (Figure 3c, c’ and d). The proportion of GFP^+^ astrocytes was increased by 1.5-fold, likely as a result of the large reduction in OLs, as the number of GFP^+^ astrocytes was not changed (61.3 ± 10.9 in experimental versus 57.2 ± 11.8 in controls; Figure 3c, c’ and d). To assess whether the reduction in OLs from *Tns3*-deleted NSCs was the consequence of a reduction in OPCs generated, we assessed for possible changes in numbers, proliferation, and survival of OPC at P11, when most cortical OPCs have not yet started differentiation. We found no differences in the proportion of GFP cells being OPCs (Figure 3e), nor the proliferative status of GFP^+^ OPCs (MCM2^+^/PDGFRα^+^ cells; Figure 3f) between experimental and control brains, while the reduction of OLs was already marked (Figure 3e), indicating that loss of *Tns3* only affected the process of OPC differentiation into OLs.

**Figure 3—figure supplement 1.**
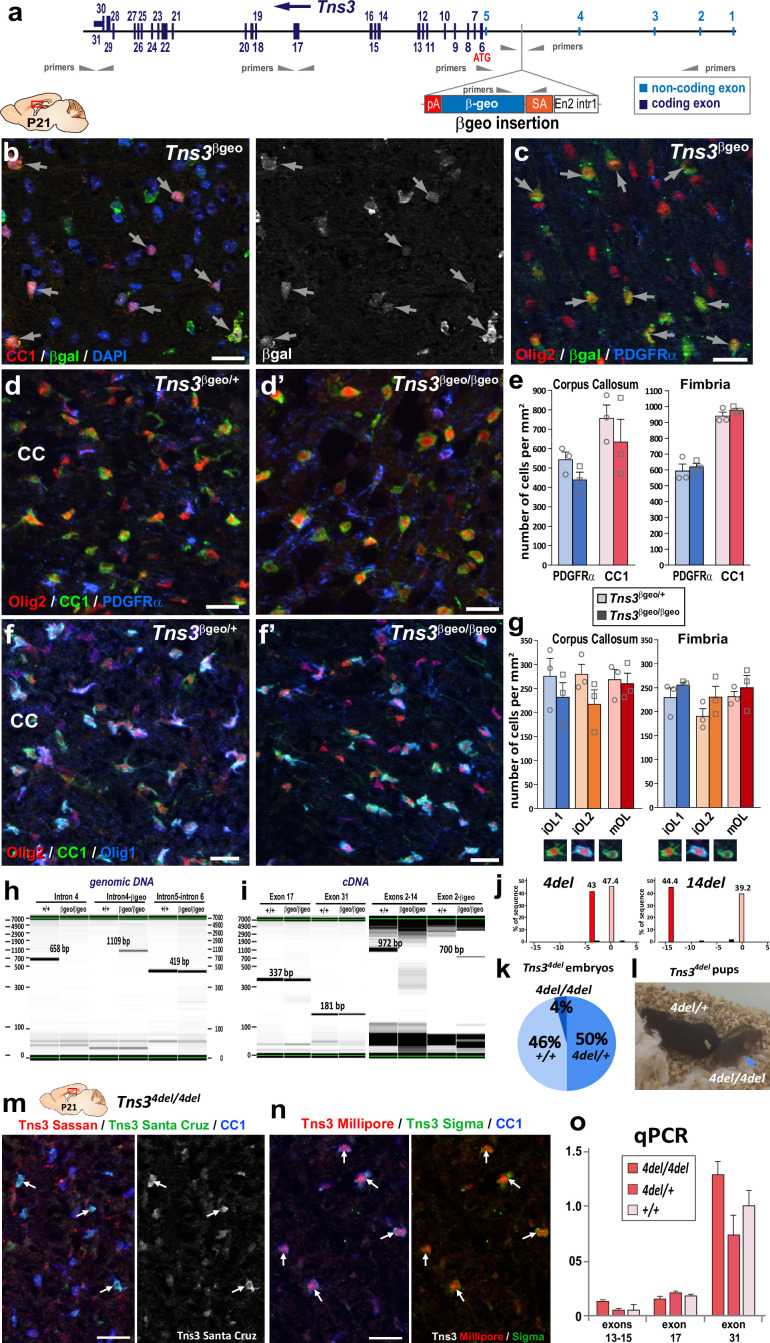
Oligodendrogenesis is normal in *Tns3* constitutive mutant mice, which still express *Tns3* full-length transcripts in the brain. (**a**) Schematic of *Tns3^βgeo^* allele, showing localization of primers used for PCR amplification. (**b, c**) β-Galactosidase immunofluorescence in P21 sagittal brain sections, corresponding to CC1^+^ oligodendrocytes (OLs) (arrows, **b**) or Olig2^high^/PDGFRα− OLs (arrows, **c**) immature OLs. (**d, d’**) Immunofluorescence for PDGFRα^+^ oligodendrocyte precursor cells (OPCs) and CC1^+^ OLs showing similar cell numbers in the corpus callosum (CC) of *Tns3^βgeo/+^* (**d**) and *Tns3^βgeo/βgeo^* (**d’**) brains. (**e**) Histograms representing density of PDGFRα^+^ OPCs and CC1^+^ OLs in the CC and the fimbria. (**f, f’**) Immunofluorescence for Olig2, CC1, and Olig1 allowing to identify three OL stages: CC1^high^/Olig1^-^ cells (iOL1), CC1^high^/Olig1^high-cytoplasmic^ cells (iOL2), and CC1^low^/Olig1^cytoplasmic^ (mOL), showing similar numbers of each OL stage in the CC of Tns*3^βgeo/+^* (**f**) and *Tns3^βgeo/βgeo^* (**f’**) brains. (**g**) Histograms representing the quantification of the density of iOL1s, iOL2s, and mOLs, in the CC and the fimbria. (**h**) PCR from genomic DNA showing the presence of the native intron 4 only in wild-type (+/+) mice, of the intron 4-βgeo only in *Tns3^βgeo/βgeo^* mice, and of intron 5/intron 6 in both mice. (**i**) Caliper visualization of PCR from cDNA showing the presence of exon 17 and exon 31 both in *Tns3^βgeo/βgeo^* and wild-type mice despite the amplification with primers for exons 2–14 only in wild-type mice, and with primers for Exon2-βgeo only in *Tns3^βgeo/βgeo^* mice. (**j**) Genotyping by TIDE analysis of *Tns3^4del^* and *Tns3^14del^* heterozygous mice showing wild-type Tns3 allele and the 4 nucleotides deletion in *Tns3^4del^* allele and the 14 nucleotides deletion in *Tns3^14del^* allele. (**k**) Pie charts representing the genotypes of E14.5 embryos obtained from *Tns3^4del^* heterozygous inter-crosses. Note the sublethal phenotype indicated by the reduced number of *Tns3^4de/4del^* embryos compared to Mendelian ratios. (**l**) *Tns3^4del^* mice presenting growth defects in homozygous pups (arrow) at P14 compared to their heterozygous littermates. (**m, n**) Immunofluorescence showing that Tns3 is still detected in CC1^+^ OLs of *Tns3^4de/4del^* P21 mice, with four different Tns3 recognizing antibodies: (**m**) Sassan Hafizi lab and Santa Cruz, (**n**) Millipore, and Sigma. (**o**) Histogram representing qPCR on cDNA from P21 brains showing no differences in the amplification of *Tns3* exons 13–15, exon 17, and exon 31 between *Tns3^4del/4del^*, *Tns3^4del/+^* or WT mice, indicating the presence of a long *Tns3* transcript containing these exons in *Tns3^4del/4del^* brains. Scale bar, 20 μm.

**Figure 3—figure supplement 2.**
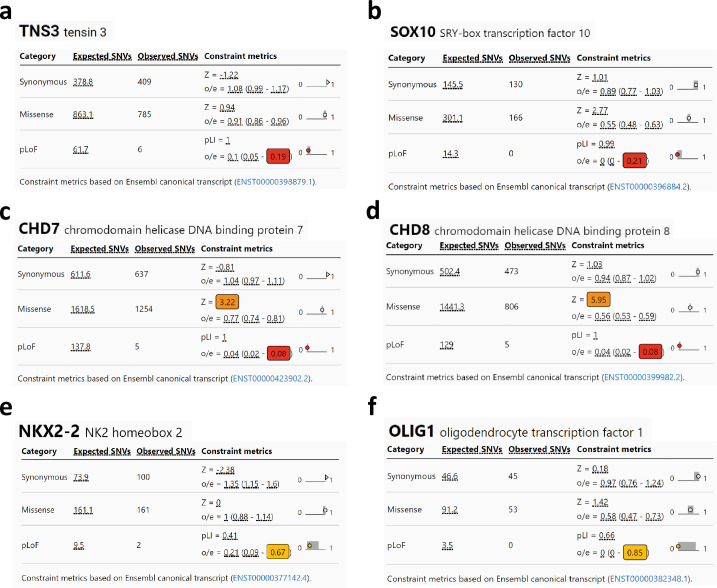
Intolerance for *TNS3* loss-of-function variants in the human population. (**a–f**) GnomAD data for *TNS3* and key regulators of oligodendrogenesis (*SOX10, CHD7, CHD8, NKX2-1*, and *OLIG1*) showing frequency and scores of different genetic variants in the human population: synonymous, missense, and predicted loss-of-function (pLOF) (nonsense, splice acceptor, and splice donor variants). The numbers highlighted colored squares correspond to the LOEF (loss-of-function observed/expected upper bound fraction), that it is a conservative estimate of the observed/expected ratio. Low LOEUF scores indicate strong selection against pLoF variation in a given gene, while high LOEUF scores suggest a relatively higher tolerance to inactivation. Note that *TNS3*, like *SOX10*, *CHD7*, and *CHD8,* has very low LOEUF scores indicating high intolerance for its inactivation, contrary to *NKX2-1* and *OLIG1,* which show more tolerance to their inactivation.

**Figure 3—figure supplement 3.**
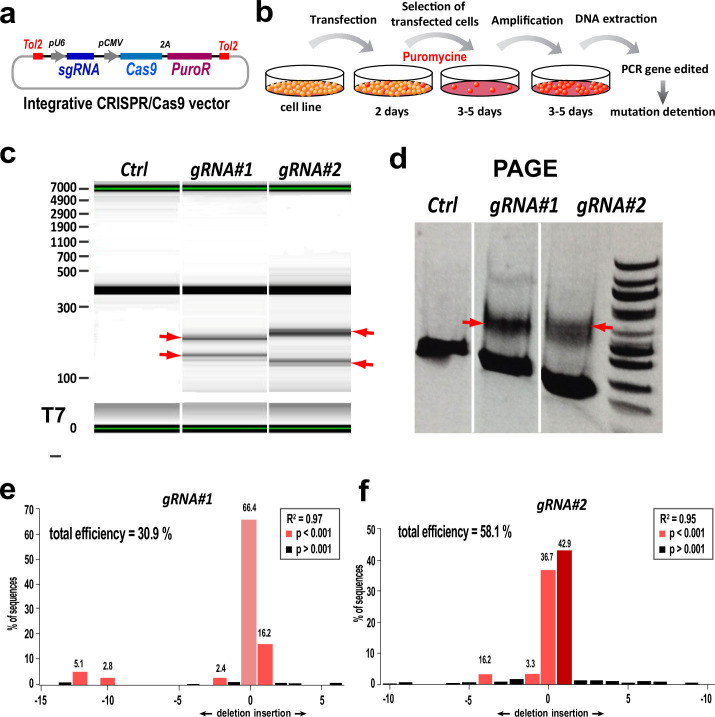
Generation and validation of *Tns3*-targeting CRISPR/Cas9 tools. (**a**) Schematic of the *Tol2-PX459* CRISPR/Cas9 expression vector allowing Tol2-DNA integration driving Cas9 and puromycin resistance expression (from polycistronic 2A-mediated cleaved) by CMV promoter and sgRNA expression from U6 promoter. (**b**) Scheme of C17.1 neuroblastoma cell line lipofection followed by puromycin selection of transfected cells used to amplify by PCR the *Tns3*-targeted region and assess for indel mutations. (**c**) Caliper visualization of PCR products obtained after T7-mediated cleavage of DNA heteroduplex due to the indel mutations, showing smaller products (arrows) generated by Cas9 cutting with gRNA#1 or gRNA#2. (**d**). Visualization of Tns3-targeted region amplified by PCR run in a PAGE gel. Note the extra bands in gRNA#1 or gRNA#2-transfected cells corresponding to Cas9 cut products, contrary to *Tol2-PX459* empty plasmid (control). (**e, f**) TIDE analysis representing the percentage of sequences presenting a given indel mutation in cells transfected with gRNA#1 (**e**) or gRNA#2 (**f**). Figure 3—figure supplement 3—source data 1.Polyacrylamine gel (PAGE) showing efficient cutting of Tns3 gRNAs #1 and #2 as extrabands formed by hybrid DNA pairing of indels and wild-type PCR products of Tns3 targeted region. Figure 3—figure supplement 3—source data 2.gRNA selection strategy, targeting either the ATG region of Tns3 locus (5') gRNA#1 and gRNA#2, and the stop codon (3') region of Tns3, gRNA#3.

Given the expression of two Tns3 isoforms in the brain (Figure 2—figure supplement 2f and g), we asked whether a deletion of both isoforms would have a greater impact in OL differentiation. We thus used two gRNAs efficiently cutting the beginning and the end of *Tns3* coding sequence (5′–3′gRNAs, ‘Materials and methods’) to delete the whole *Tns3* locus. We found a similar reduction of OLs in the loss of the two *Tns3* isoforms than in mutations affecting only full-length Tns3 (Figure 3h, h’ and i), suggesting that the small Tns3 isoform does not play an additional role with full-length Tns3 in OL formation. Altogether, these results indicate that *Tns3* loss-of-function mutations in neonatal SVZ-NSCs impair OPC differentiation without apparent changes in OPC generation and proliferation, thus suggesting that Tns3 is largely required for OPC differentiation into OLs in the postnatal brain (Figure 3g).

### OPC-specific *Tns3* deletion impairs oligodendrocyte differentiation in the postnatal brain

**Figure 4.**
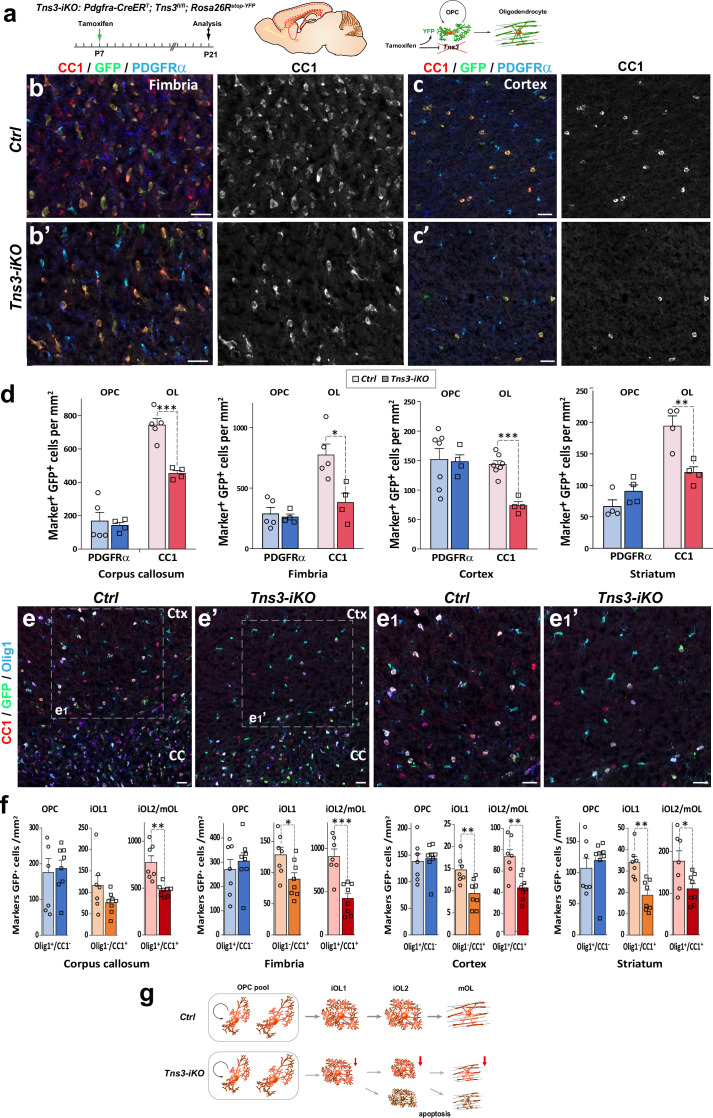
Oligodendrocyte precursor cell (OPC)-specific *Tns3* deletion reduces the number of differentiating oligodendrocytes (OLs) in the postnatal brain. (**a**) Scheme of tamoxifen administration to *Tns3-iKO* and control (*Cre^+^; Tns3^+/+^*) mice, Cre-mediated genetic changes, and timing of experimental analysis. (**b, b’, c, c’**) Immunofluorescence in P21 sagittal brain sections for CC1, GFP, and PDGFRα illustrating similar density of OPCs (PDGFRα^+^) and twofold reduction in OL (CC1^+^) density in *Tns3-iKO* (**b’, c’**) compared to control (**b, c**) in the fimbria (**b**) and the cortex (Ctx) (**c**). (**d**) Histograms showing OPC and OL density in P21 *Tns3-iKO* and control (*Ctrl*) mice, in the corpus callosum (CC), fimbria, Ctx, and striatum. Note the systematic OL decrease of 40–50% in each region. (**e–e_1_’**) Immunofluorescence in P21 sagittal brain sections for Olig1, GFP, and CC1 to distinguish three stages of oligodendrogenesis: OPCs (Olig1^+^/CC1^-^), iOL1s (CC1^+^/Olig1^-^), and iOL2s/mOLs (CC1^+^/Olig1^+^) in *Ctrl* (**e**) or *Tns3-iKO* mice (**e’**). (**e_1_**) and (**e_1_’**) are higher magnification of the squared area in (**e**) and (**e**’). (**f**) Histograms showing the OPCs, the iOL1s, and the iOL2s/mOLs density in P21 *Tns3-iKO* and control mice, in the CC, fimbria, Ctx, and striatum. Note the decrease of iOL1s and iOL2s over 40% in each area quantified (except for iOL1 density in the CC). (**g**) Schematic representing defects in oligodendrogenesis found in *Tns3-iKO* compared to control. Scale bar, 20 μm.

Given the heterogeneity of CRISPR/Cas9-mediated indels and the difficulties to assess in vivo the penetrance of their *Tns3* loss of function, to address in more detail the consequences of *Tns3* loss of function, we designed a *Tns3* conditional knockout allele by flanking with LoxP sites the exon 9 (Figure 4—figure supplement 1). In order to specifically delete *Tns3* in postnatal OPCs, we administered tamoxifen at P7 to *Pdgfra-CreER^T^; Tns3^fl/fl^; Rosa26^stop^*^-*YFP*^ (hereafter called *Tns3-iKO* mice) and control pups (*Pdgfra-CreER^T^; Tns3^+/+^; Rosa26^stop^*^-*YFP*^ littermates) and analyzed its effects on oligodendrogenesis at P14 and P21 (Figure 4a) both in white matter (corpus callosum and fimbria) and gray matter regions (cortex and striatum). We first assessed for the efficiency of *Tns3* deletion in Nkx2.2^+^/GFP^+^ iOLs from different regions by immunofluorescence using a Tns3 antibody (Sigma Ct), finding that the strong Tns3 signal present in Nkx2.2^+^/GFP^+^ iOLs of control brains was almost completely eliminated in *Tns3-iKO* iOLs without affecting Tns3 expression in vessels (Figure 4—figure supplement 2b,b',c, arrows and arrowheads versus asterisks). We then assessed for changes in oligodendrogenesis. Remarkably, the number of OLs (CC1^+^/GFP^+^ cells) was reduced by half in all quantified regions (reduction of 38.95% in the CC, 48.60% in cortex, 50.88% in the fimbria, 38% in the striatum; Figure 4b–d, Figure 4—figure supplement 2e–f', Figure 4—figure supplement 3b and b') in *Tns3-iKO* compared to control, while OPC (PDGFRα^+^/GFP^+^ cells) density was unchanged (Figure 4b–d). Using markers distinguishing different stages of OL differentiation (iOL1 and iOL2/mOL), we found that the density of iOL1s (Nkx2.2^high^ cells), which express the highest levels of Tns3 protein in control brains, was unchanged (Figure 4—figure supplement 2b,b',d), while the density of early iOL2s (CC1^+^/Olig1^-^ cells) and later OL stages (iOL2/mOLs CC1^+^/Olig1^+^ cells) was reduced by 30 and 50%, respectively, in *Tns3-iKO* compared to controls (Figure 4e–f), suggesting that Tns3 is required for normal OL differentiation. Finally, we assessed possible changes in OPC proliferation by immunodetection of Mcm2, finding no significant changes in the proliferation of *Tns3-iKO* OPCs compared to control OPCs (Figure 4—figure supplement 3c,c',d).

**Figure 4—figure supplement 1.**
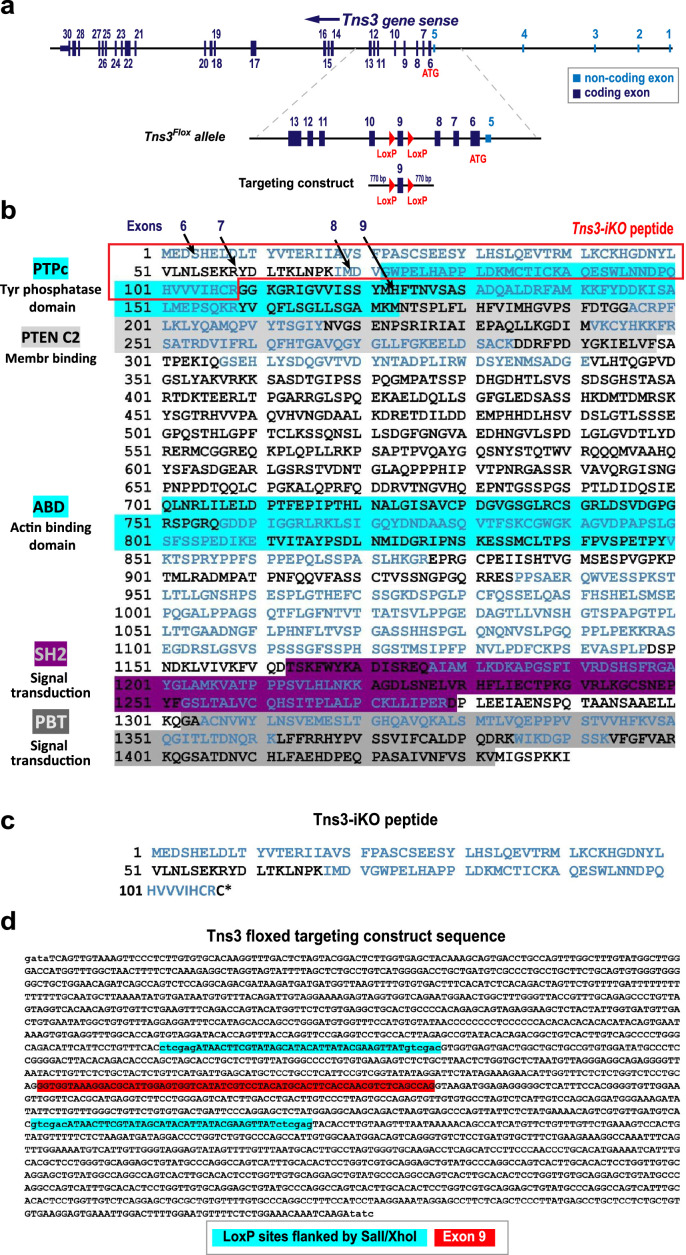
Generation of *Tns3* floxed allele. (**a**) Schematic of *Tns3* locus, *Tns3^Flox^* allele, and targeting vector with LoxP sites flanking exon 9 used to induce homologous recombination in mouse ESCs. (**b**) Full-length Tns3 amino acid sequence indicating its protein domains. Squared in red highlights the 109 amino acid sequence of the Tns3 peptide produced upon Cre-mediated recombination in *Tns3^Flox^*-expressing cells. (**c**) Peptide sequence of protein product predicted for *Tns3-iKO*-expressing cells. (**d**) *Tns3^Flox^* targeting vector sequence used for homologous recombination in mouse ESCs and generation of *Tns3^Flox^* mice, with LoxP sites highlighted in blue and exon 9 highlighted in red.

**Figure 4—figure supplement 2.**
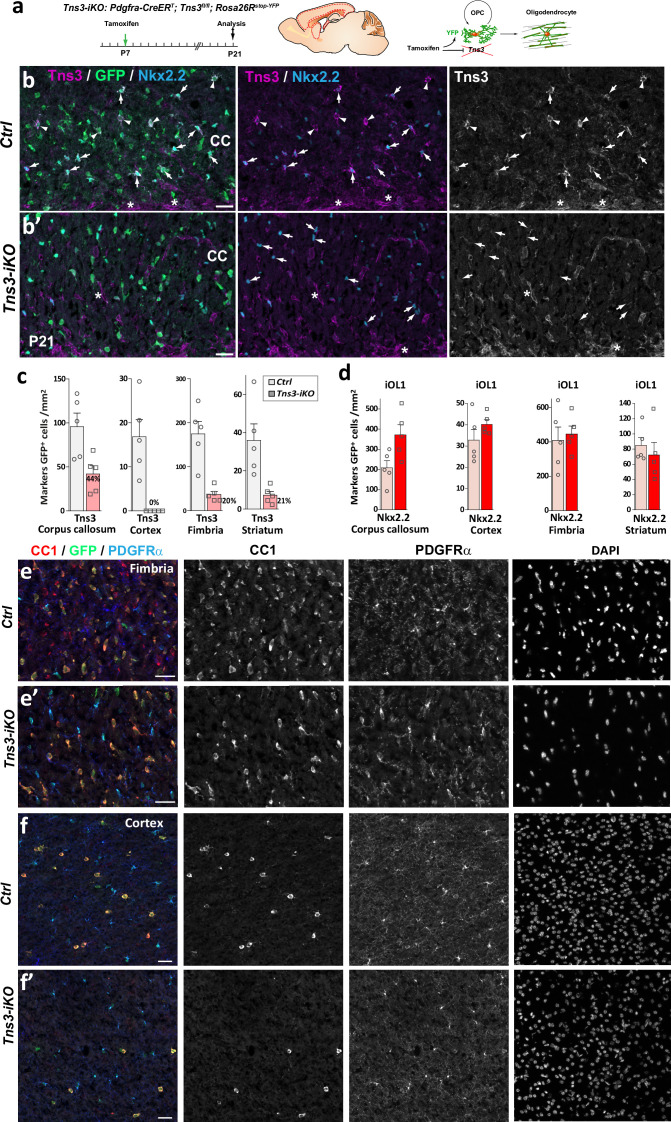
Efficient oligodendrocyte precursor cell (OPC)-specific *Tns3* deletion in the postnatal brain and reduced oligodendrocyte generation from OPCs. (**a**) Scheme of tamoxifen administration to *Tns3-iKO* (*Cre^+^; Tns3^fl/fl^*) and control (*Cre^+^; Tns3^+/+^*) mice, Cre-mediated genetic changes, and timing of experimental analysis. (**b, b’**) Immunofluorescence for Tns3, GFP, and Nkx2.2 in P21 mice sagittal brain sections at the level of the corpus callosum (CC), illustrating the loss of Tns3 signal in Nkx2.2^high^ iOL1s (arrows) but not in vessels (asterisk) of *Tns3-iKO* (**b’**) compared to control (**b**). Note that Nkx2.2^high^ iOL1s do not change in density in *Tns3-iKO* compared to control. (**c**) Histograms showing the Tns3+ cells density in the CC, fimbria, cortex, and striatum, in *Tns3-iKO* and control. (**d**) Histograms showing the Nkx2.2^high^ iOL1s density in the CC, fimbria, cortex, and striatum, in *Tns3-iKO* and control. (**e–f’**) Immunostaining corresponding to Figure 4b–c’ showing individual channels for CC1, PDGFRα, and DAPI in the fimbria (**e, e’**) and cortex (**f, f’**) of control mice (**e, f**) and *Tns3-iKO* mice (**e’, e’**). Scale bar, 20 μm.

**Figure 4—figure supplement 3.**
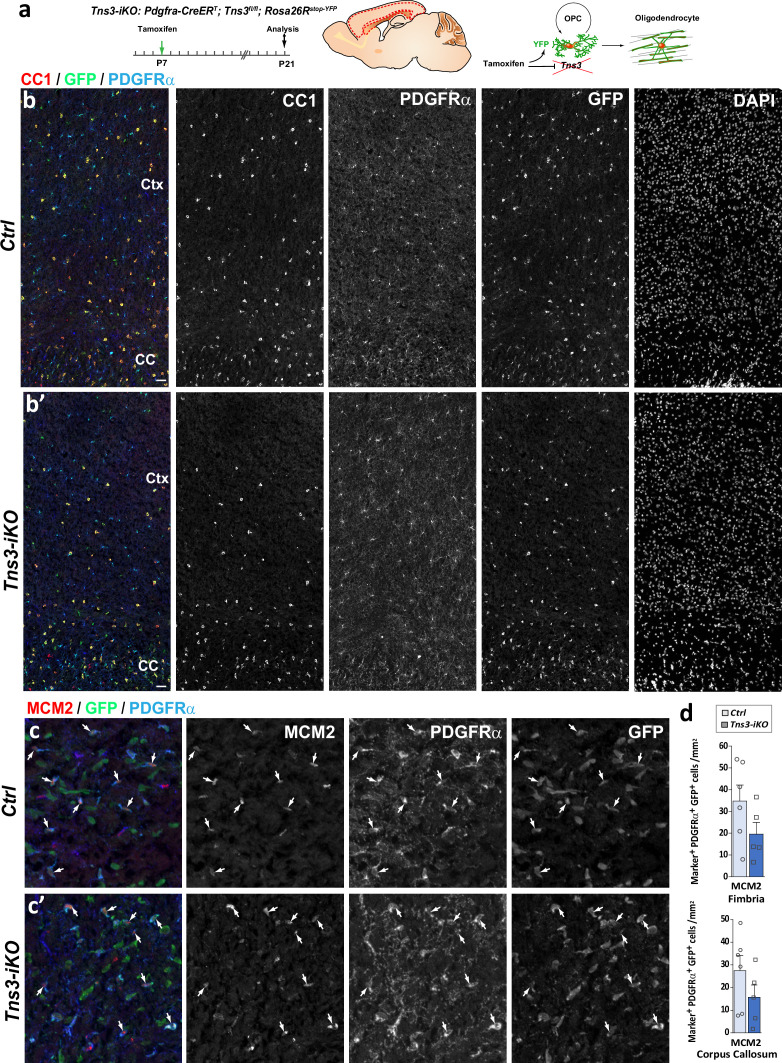
Oligodendrocyte precursor cell (OPC)-specific *Tns3* deletion reduces oligodendrocyte (OL) generation without changing OPC proliferation in the postnatal brain. (**a**) Scheme of tamoxifen administration to *Tns3-iKO* (*Cre^+^; Tns3^fl/fl^*) and control (*Cre^+^; Tns3^+/+^*) mice, Cre-mediated genetic changes, and timing of experimental analysis. (**b, b’**) Immunostaining illustrating at low magnification the reduction in number of CC1^+^ OLs but not PDGFRα^+^ OPCs in the cortex (Ctx) and corpus callosum (CC) of *Tns3-iKO* mice (**b’**) compared to control mice (**b**), showing each individual channel and DAPI. (**c, c’**) Immunofluorescence for MCM2 proliferative marker, GFP, and PDGFRα illustrating similar proportion of proliferative OPCs in control (**c**) and *Tns3-iKO* mice (**c’**). (**d**) Histograms quantifying the proliferative fraction of GFP^+^ OPCs in the fimbria (upper) and CC (lower). Note the slight reduction (nonsignificant) in proliferation in *Tns3-iKO* OPCs compared to control OPCs. Scale bar, 20 μm.

Altogether, these results indicate that acute deletion of *Tns3* in OPCs reduces by twofold generation of OLs in the postnatal brain, without major changes in OPC numbers and proliferation (Figure 4g).

### *Tns3-iKO* oligodendroglia undergo apoptosis

**Figure 5.**
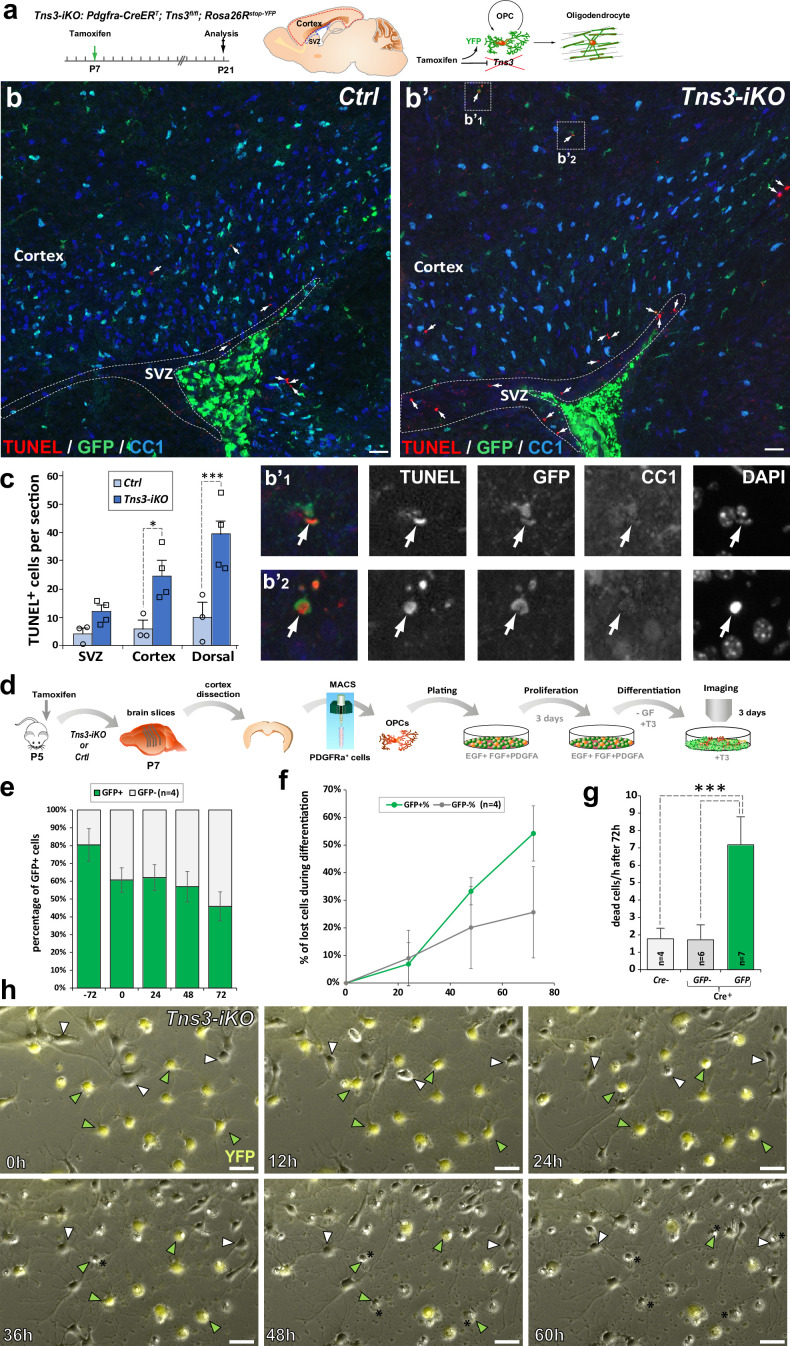
Increased cell death of *Tns3-iKO* oligodendroglia. (**a**) Scheme of tamoxifen administration to *Tns3-iKO* (*Cre^+^; Tns3^fl/fl^*) and control (*Cre^+^; Tns3^+/+^*) mice, Cre-mediated genetic changes, and timing of experimental analysis. (**a–b’**) Immunodetection of death cells by TUNEL together with recombined cells (GFP) and oligodendrocytes (OLs) (CC1^+^ cells) showing increased number of TUNEL^+^ cells in the cortex and corpus callosum of *Tns3-iKO* mice (**b’**) compared to control mice (**b**), including some GFP^+^/CC1^−^ oligodendrocyte precursor cells (OPCs) (insets **b’_1_** and **b’_2_**). (**c**) Histograms quantifying the number of TUNEL^+^ cells in the subventricular zone (SVZ), cortex, and both together (dorsal) per section. (**d**) Scheme of the video microscopy protocol in MACSorted OPCs purified from *Tns3-iKO* and control P7 mice. Cells were imaged every 10 min during 72 hr in differentiation medium. (**e**) Histograms showing the reduction of the GFP^+^ cells proportion from the plating (–72 hr) to the end of the experiment (72 hr after differentiation onset). (**f**) Time curve quantifying the loss of GFP^+^ OLs compared to GFP^-^ OLs during the 72 hr of differentiation. (**g**) Histograms representing the quantification of cells lost per hour during the 72 hr differentiation period, showing a fivefold increase in loss of GFP^+^ *Tns3-iKO* cells compared to GFP^-^ cells (nonrecombined cells from *Tns3-iKO* mice, internal negative control) or cells coming from *Cre^−^* littermates (*Cre^−^*, external negative control). (**h**) Time-lapse frames showing cells every 12 hr illustrating both GFP^+^ (green arrowheads, recombined *Tns3-iKO* cells) and GFP^-^ (white arrowheads, nonrecombined *Tns3-iKO* cells) that die over the time of video microscopy. Note the larger number of GFP^+^ OLs (cells with multibranched OL morphology) dying compared to GFP^-^ OLs. Scale bar, 20 μm.

Tensins are known to mediate integrin stabilization and activation in other cell types (Liao and Lo, 2021), with Tns3 been shown to bind integrin-β1 through its phosphotyrosine-binding domain and FAK through its SH2 domain in fibroblasts (Cui et al., 2004; Liao et al., 2007; Georgiadou et al., 2017). In oligodendroglia, integrin α6β1 association with Fyn kinase is required to amplify PDGF survival signaling and promote myelin membrane formation by switching neuregulin signaling from a PI3K to a MAPK pathway (Colognato et al., 2004). Moreover, by conditional ablation of integrin-β1 in vivo, it was demonstrated that integrin-β1 signaling is involved in survival of differentiating oligodendroglia, but not required for axon ensheathment and myelination per se (Benninger et al., 2006). We, therefore, investigated the expression of genes involved in integrin signaling in the transcriptome of oligodendroglial cells. Indeed, *Tns3* expression pattern in iOLs was closely matching that of *Itgb1* (integrin-b1), *Fyn*, *Bcar1*/*p130Cas*, and *Ptk2*/*Fak* both in mouse and human oligodendroglia (Figure 5—figure supplement 1a and b). Furthermore, using neural progenitor differentiation cultures, we observed co-expression of integrin-β1 and Tns3 in CNP^+^ OLs by immunofluorescence (Figure 5—figure supplement 1c), suggesting that Tns3 could relay integrin-β1-mediated survival signal in differentiating oligodendroglia. Therefore, we assessed for signs of cell death in *Tns3-iKO* oligodendroglia by performing the TUNEL technique together with GFP and CC1 immunodetection. Interestingly, we found a fivefold increase in TUNEL^+^ cells in the dorsal telencephalon of *Tns3-iKO* brain, compared to control, without significant changes in non-oligodendroglial cells present in the SVZ (Figure 5a–c). To gain more insight into the cellular alterations and cell death of *Tns3-*deleted oligodendroglia, we investigated their cellular morphology and behavior by video microscopy during their differentiation in culture. To this end, we MACS-purified OPCs from *Tns3-iKO* and control (*Tns3^flox/flox^; Rosa26^stop^*^-YFP^ littermates) mice at P7, 2 days after administration of tamoxifen, plated them in proliferating medium for 3 days, and recorded their behavior during 3 days in the presence of differentiation medium (Figure 5d). Using the expression of the YFP as a readout of Cre-mediated recombination, we compared the behavior of YFP^+^ cells (*Tns3-iKO*) with neighboring YFP^-^ cells (internal control) in the same cultures. In parallel, we used MACSorted cells from control mice as external control. Quantification of the proportion of YFP^+^ cells over time showed a 20% reduction of YFP^+^ cells (from 80% to 60%) during the 3 days in proliferation medium followed by a reduction to 50% by day 3 in differentiation medium (Figure 5e), suggesting possible cell death of *Tns3*-mutant cells. Live-imaging monitoring of cell behavior showed that once YFP^+^ cells had developed multiple branched morphology, characteristic of differentiating OLs, they showed a fourfold increase in their probability to die compared to YFP^−^ cells of the same culture (Figure 5f–h, yellow and white arrows, respectively) or to cells from control cultures, with more pronounced cell death by the third day of culture (Figure 5f and g). Together, these results indicate that *Tns3-iKO* oligodendroglia present increased cell death both in vivo and in primary cultures at the stage when Tns3 is upregulated and cells start to develop their branched morphology, suggesting that Tns3 likely mediates β1-integrin signaling required for their survival.

**Figure 5—figure supplement 1.**
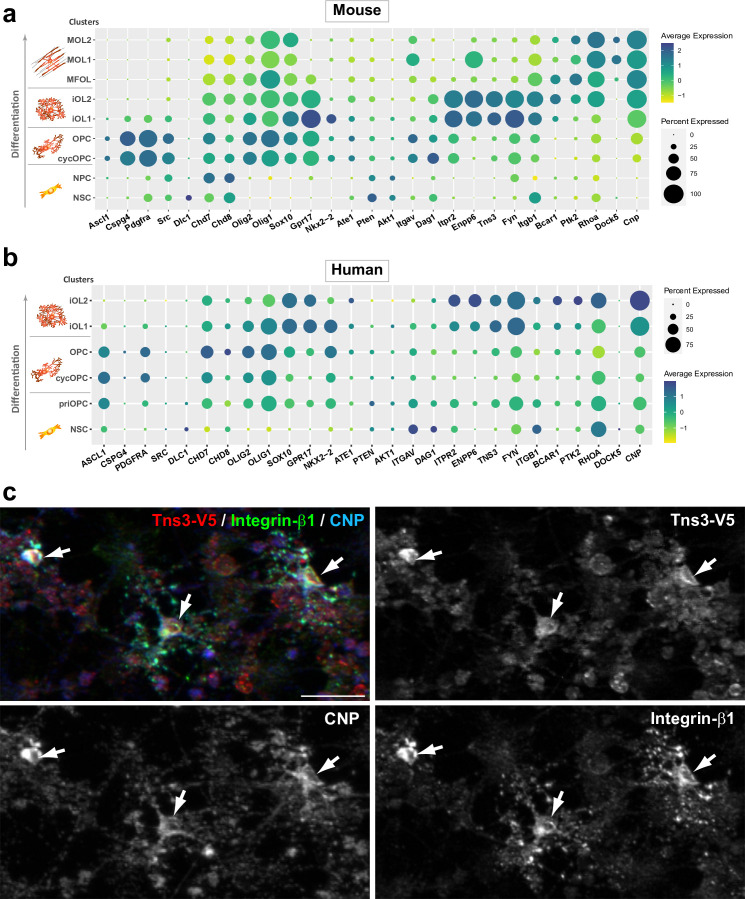
*Tns3* is co-expressed with integrin signaling genes in immature oligodendrocytes (iOLs). (**a, b**) Dot plots showing *Tns3/TNS3* expression pattern in mouse (**a**) and human (**b**) single cells at different stages of oligodendrogenesis together with key markers of each cluster and genes involved in integrin signaling pathway. Note the high levels of Tns3/TNS3 in iOLs (iOL1 and iOL2) together with known genes of integrin pathway previously implicated in oligodendrogenesis (*Itgb1, Fyn, FAK/Bcar1, p130Cas/Ptk2*) both in mouse and human cells. (**c**) Immunofluorescence in neurosphere-derived neural progenitor cultures generated from *Tns3^Tns3-V5^* neonatal brain after 3 days of differentiation showing co-expression of Tns3-V5 (V5 antibody) and Integrin-β1 in CNP^+^ differentiating OLs (arrows). Scale bar, 20 μm.

### Apoptosis of *Tns3-iKO* oligodendroglia is mediated by p53 upregulation

**Figure 6.**
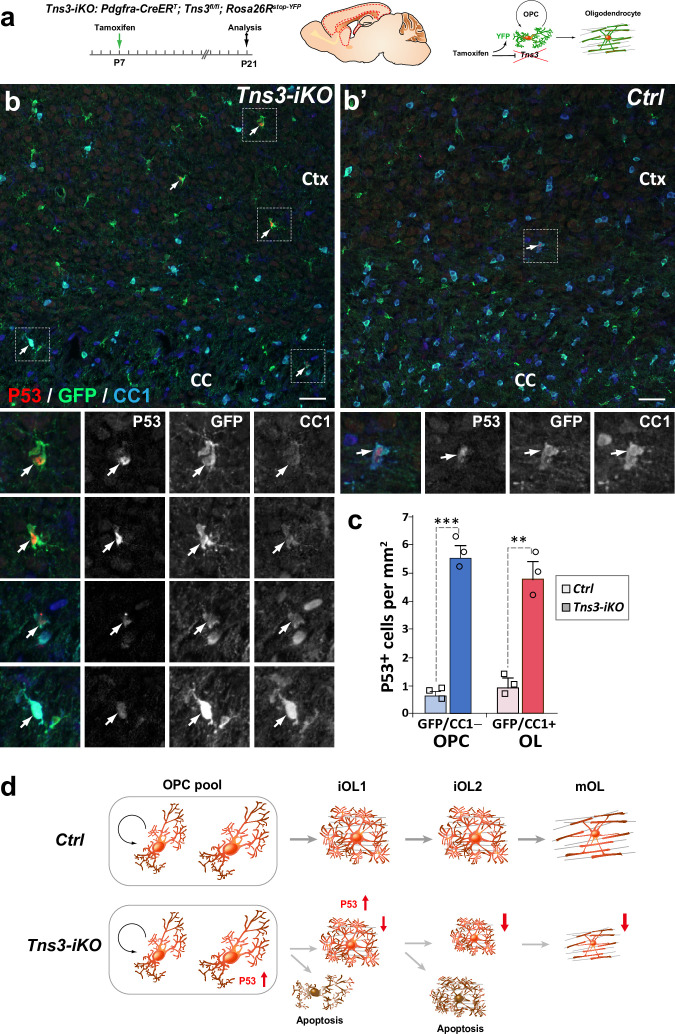
p53-mediated cell death of *Tns3-iKO* oligodendroglia. (**a**) Scheme of tamoxifen administration to *Tns3-iKO* (*Cre^+^; Tns3^fl/fl^*) and control (*Cre^+^; Tns3^+/+^*) mice, Cre-mediated genetic changes, and timing of experimental analysis. (**b, b’**) Immunodetection of p53 together with GFP to label recombined cells and CC1 to label oligodendrocytes (OLs) showing a strong increased number of p53^+^/GFP^+^/CC1^−^ oligodendrocyte precursor cells (OPCs) and p53^+^/GFP^+^/CC1^+^ OLs in the cortex (Ctx) and corpus callosum (CC) of *Tns3-iKO* mice (**b**) compared to control mice (**b’**). Dotted squares highlight some cases of p53^+^ cells, shown at higher magnification below. (**c**) Histograms quantifying the number of p53^+^ cells per area (mm^2^) in the dorsal telencephalon. Scale bar, 20 μm. (**d**) Schematics representing *Tns3*-deleted phenotypes in oligodendroglia. Scale bar, 20 μm.

To study the molecular mechanisms of Tns3 function in oligodendroglia, we first looked at p53 expression, the master transcriptional regulator of the cellular genotoxic stress response (Kastenhuber and Lowe, 2017; Aubrey et al., 2018). Interestingly, we found a tenfold increase in p53^+^ OPCs (GFP^+^/CC1^-^ cells) and fourfold increase in p53^+^ iOLs (GFP^+^/CC1^+^ cells) in *Tns3-iKO* compared to control (Figure 6a–c), suggesting that the loss of Tns3 leads to an upregulation of p53, which, together with the loss of integrin-β1 survival signal, mediates the cell death of *Tns3-iKO* differentiating oligodendroglia (Figure 6d).

### *Tns3-iKO* oligodendroglia shows transcriptional dysregulation of genes involved in OPC differentiation, apoptosis, integrin signaling, and cell cycle regulation

**Figure 7.**
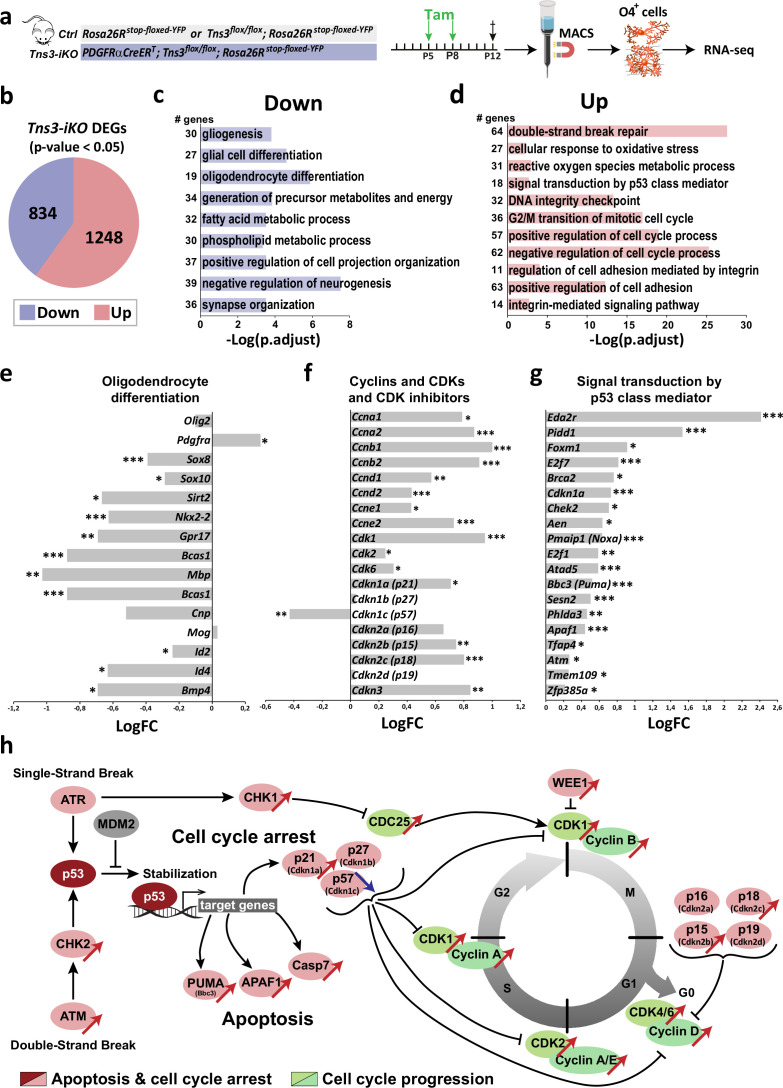
Mechanisms involved in *Tns3-iKO* oligodendroglial defects. (**a**) Diagram representing tamoxifen (Tam) injection in P5 and P8 *Ctrl* and *Tns3-iKO* pups followed by MACSorting of O4^+^ cells that were used to do RNA-seq. (**b**) Pie chart showing the amount of differentially expressed genes (DEGs; p-value<0.05) in *Tns3-iKO* O4^+^ cells compared to *Ctrl*. (**c, d**) Gene Ontology (GO) analysis of biological processes downregulated (**c**) and upregulated (**d**) in *Tns3-iKO* O4^+^ cells compared to *Ctrl*. Numbers on left represent the number of deregulated genes in each GO process. (**e–g**) Graphs representing the logarithmic fold change (LogFC) for an example of genes involved in oligodendrocyte differentiation (**e**), cell cycle (**f**), and p53 pathway (**g**). (**h**) Summary schematics of the transcriptional dysregulation of *Tns3-iKO* O4^+^ cells, representing the upregulation of the apoptosis pathway and the conflicting signals on cell cycle arrest/progression. Red and blue arrows represent gene upregulation and downregulation, respectively, in *Tns3-iKO* O4^+^ cells compared to *Ctrl*.

To further unravel the defects of *Tns3*-deleted oligodendroglia, we purified oligodendroglia (O4^+^ cells) from P12 *Tns3-iKO* and control cortices by MACS (Figure 7a). Upon validation of Tns3 deletion at the transcript and protein levels (Figure 7—figure supplement 1a–c) and that similar proportion of oligodendroglia were present in each genotype (Figure 7—figure supplement 1d–f), we compared their transcriptomes obtained by bulk RNA sequencing (Figure 7a). Principal component analysis of Tns3-iKO and control samples shows clear separation between the groups (Figure 7—figure supplement 1g). Statistical analyses using edgeR (Chen et al., 2016) showed 2082 differentially expressed genes (DEGs, p-value<0.05) between *Tns3-iKO* and control, with 834 downregulated and 1248 upregulated genes (Figure 7b). Gene Ontology (GO) analysis of biological processes indicated that main downregulated processes were involved in terms related OL differentiation (including gliogenesis, glial cell differentiation, OL differentiation, lipid metabolism, and positive regulation of cell projection organization; Figure 7c and e, Supplementary file 1), while the upregulated biological processes related to terms such as cellular stress and p53 pathway (including double-strand break repair, cellular response to oxidative stress, and signal transduction of p53 class mediator), opposite processes involved in cell cycle regulation (including DNA integrity check point, G2/M transition of mitotic cell cycle, and positive- and negative regulation of cell cycle process) (Figure 7d, f and g, Supplementary file 1). Interestingly, GO processes related to integrin signaling and cell adhesion were upregulated (including positive regulation of cell adhesion, regulation of cell adhesion mediated by integrin, integrin-mediated signaling pathway, and positive regulation of cell adhesion; Figure 7d, Supplementary file 1), with several integrin transcripts upregulated (*Itgam, Itga8, Itgb2, Itga4, Itgb3, Itgb5, Itga6, Itgb8,* which are normally not expressed in OPCs/iOLs; Supplementary file 1), while *Fyn,* Src family kinase that associates with α6β1 and is required to amplify PDGF survival signaling (Colognato et al., 2004) was downregulated (1.4-fold, p-value=0.03; Supplementary file 1), suggesting that Tns3 deletion impairs normal integrin signaling, and as a consequence *Tns3*-deleted cells try to compensate this impairment upregulating of other integrin family members. These results, confirming and expanding those obtained by immunofluorescence analyses, led us to propose a model suggesting that *Tns3*-deleted oligodendroglia present signs of cellular stress accompanied by double-strand break signaling upregulation (including ATM and CHK2 regulators), p53 stabilization and upregulation of p53 target genes involved in apoptosis (including PUMA, APAF1, and Caspase 7; Figure 7h), and conflicting signals related to cell cycle (upregulation of p21, promoting cell cycle arrest, and upregulation of CDK/cyclin complexes promoting cell cycle progression; Figure 7h).

**Figure 7—figure supplement 1.**
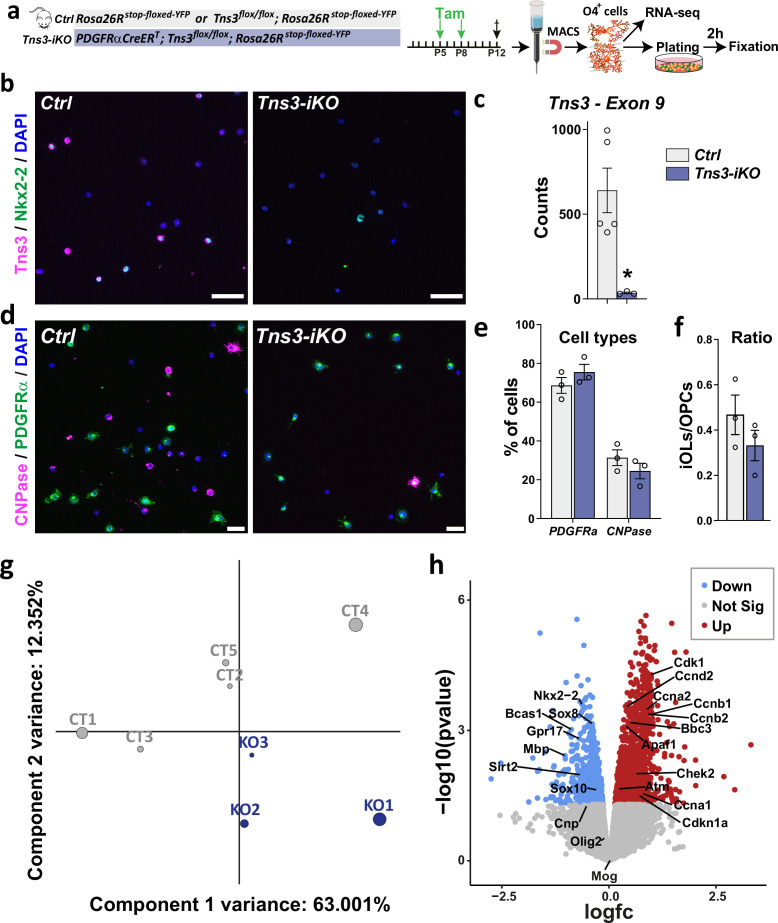
Transcriptomic analysis of acutely Tns3-deleted oligodendroglia. (**a**) Diagram representing tamoxifen (Tam) injection at P5 and P8 Ctrl and *Tns3-iKO* pups followed by the MACSorting of O4^+^ cells used for RNA-seq and plating. Cells were fixed 2 hr after and used to do immunostaining. (**b**) Immunostaining validating the loss of Tns3 protein in *Tns3-iKO* O4^+^ cells compared to Ctrl. (**c**) Graph representing the count of exon 9 of Tns3 in the RNA-seq data. Note the reduction of floxed exon 9 in the *Tns3-iKO* O4^+^ cells compared to control (*Ctrl*). (**d**) Immunostaining showing the composition in oligodendrocyte precursor cells (OPCs) (PDGFRα^+^ cells) and immature oligodendrocytes (iOLs) (CNPase^+^ cells) among *Ctrl* and *Tns3-iKO* O4^+^ cells after MACS. (**e**) Graph indicating that the same amount of OPCs (PDGFRα^+^ cells) and iOLs (CNPase^+^ cells) was found in *Ctrl* and *Tns3-iKO* O4^+^ cells after MACS. (**f**) Graph showing that the ratio of iOLs (CNPase^+^ cells)/OPCs (PDGFRα^+^ cells) was not altered in *Tns3-iKO* O4^+^ cells compared to Ctrl. (**g**) Principal component analysis (PCA) of the RNA-seq data transcripts of *Ctrl* (CT) and *Tns3-iKO* (KO) O4^+^ cells. (**h**) Volcano plot showing the upregulated (red) and downregulated (blue) in *Tns3-iKO* O4^+^ cells compared to *Ctrl*. Some example of genes can be found. Scale bars: (**b**) 50 μm; (**d**) 10 μm.

## Discussion

The tight balance of OPCs between proliferation, survival, and differentiation ensures their capacity to respond to the myelination needs of the CNS by generating new OLs on demand, whilst avoiding the generation of brain gliomas through uncontrolled OPC proliferation. The observation that OPCs are present within demyelinating MS lesions but fail to efficiently differentiate into myelinating cells with age and disease progression (Chang et al., 2002; Neumann et al., 2019), together with the strong sensitivity of iOLs to survival/apoptotic signals (Hughes and Stockton, 2021), suggests that efforts to foster OPC differentiation and survival of iOLs are a critical events for healthy aging and successful remyelination in MS patients. In this study, we combined the genome-wide binding profile of key regulators of OL differentiation, Olig2, Chd7, and Chd8 (Lu et al., 2000; Zhou et al., 2000; Lu et al., 2002; Zhou and Anderson, 2002; He et al., 2016; Küspert and Wegner, 2016; Marie et al., 2018; Zhao et al., 2018), to identify their common gene targets, and focused our analysis on Tensin3 (Tns3), whose expression matched the onset of OL differentiation. To study Tns3 expression and function, we generated several genetic tools, including CRISPR/Cas9 vectors, to induce *Tns3* mutations both in vivo and in vitro, a *Tns3^Tns3^*^-V5^ knock-in mouse, two constitutive *Tns3* knockout mice, and finally an inducible knockout (*Tns3^Flox^*) mouse. Using these tools, we provide several lines of evidence showing that Tns3 is upregulated in iOLs and required for normal OL differentiation. First, we show that Tns3 expression is strongly induced at the onset of OL differentiation, localized to the cytoplasm and main cell processes of iOLs, and downregulated in mature OLs both at the transcript and protein levels, thus constituting a novel marker for iOLs, for which we provide an optimal immunofluorescence protocol with a commercial antibody (Sigma, Ct). Second, we show that during remyelination Tns3 is also expressed in newly formed OLs and thus could be used as a hallmark for ongoing remyelination. Third, analyzing both *Tns3^βgeo^* gene trap mice and two *Tns3^KO^* mice, we show that constitutive *Tns3* deletion is detrimental for normal development and that the predicted loss of Tns3 full-length transcript and protein is bypassed in the oligodendroglia of surviving homozygous animals, paralleling the intolerance for *TNS3* loss-of-function variants found in the human population. Fourth, in vivo CRISPR-mediated *Tns3* deletion in neonatal NSCs from the SVZs leads to a twofold reduction of OLs without changes in OPC generation, proliferation, and numbers. Fifth, in vivo Tns3-induced knockout (*Tns3-iKO*) in postnatal OPCs leads to a twofold reduction of differentiating OLs without reducing the overall OPC population, both in gray and white matter brain regions. Finally, we provide evidence, by immunodetection in vivo and video microscopy of primary OPC differentiation cultures, that *Tns3-iKO* differentiating oligodendroglia upregulate p53, key sensor of cell stress, and present a four- to fivefold increase in apoptosis compared to control oligodendroglia, suggesting that mechanistically Tns3 function is likely required for normal OL differentiation at least in part by mediating integrin-β1 survival signaling in differentiating oligodendroglia.

### Tns3 is a novel marker for immature oligodendrocytes

Recent studies have started to uncover genes enriched in iOLs, such as *Itpr2* (Zeisel et al., 2015; Marques et al., 2016), *Enpp6* (Xiao et al., 2016), and *Bcas1* (Fard et al., 2017), that could be used as markers for these transient cell populations, particularly interesting to label areas of active (re)myelination in the context of OL and myelin pathology, such as preterm brain injury and MS. Here, we report for the first time that Tns3 is a hallmark of iOLs (Figure 2). Tns3 is expressed at high levels in iOLs and downregulated as OLs mature into myelinating cells, showing a complete overlap with Itpr2 transcript and protein. We found that a commercial Tns3 antibody (Millipore) also recognizes another nuclear protein that, like Tns3 in the cytoplasm, also labels at high levels iOLs, paralleling the case of CC1 antibody, which recognizes both APC and Quaking-7 proteins in OLs (Lang et al., 2013; Bin et al., 2016). Upon testing several antibodies, we found one (Sigma Ct) optimally labeling iOLs by immunofluorescence in brain sections and oligodendroglial cell cultures, whereas the Itpr2 commercial antibody we tried did not match this high-quality iOLs immunolabeling. An optimized protocol for immunodetection using Bcas1-recognizing antibodies has been shown to label iOLs (Fard et al., 2017). Finally, Enpp6 is very specific for iOLs at the transcript level (Xiao et al., 2016), but to our knowledge, no Enpp6-recognizing antibodies producing good quality immunodetection are yet available. Therefore, Tns3 protein expression in the CNS is a hallmark of iOLs, and the Tns3 Sigma Ct antibody is an optimal reagent to label iOLs during both myelination and remyelination.

### Tns3 is required for oligodendroglial differentiation

OL differentiation involves substantial generation of new membrane and cell processes composing the 40–60 myelin segments formed by mature OLs (Hughes et al., 2018). Actin cytoskeleton remodeling is an important driver of the OL morphological changes undergone during their differentiation (Nawaz et al., 2015; Zuchero et al., 2015). Tensin proteins, linking the extracellular signals received by transmembrane integrins with the actin cytoskeleton in different cell types (Liao and Lo, 2021), are well placed to play an important role in these morphological changes. At the molecular level, it has been shown that the phosphotyrosine-binding domains of Tensins interact with the NPXY motifs present in the cytoplasmic tails of integrin-β1 in a pTyr-insensitive fashion (Calderwood et al., 2003; Katz et al., 2007; McCleverty et al., 2007), allowing Tensins to bring actin filaments, through their actin binding domain, to focal adhesion sites (Liao and Lo, 2021). Given that the extension of OL cell processes’ growth cone is guided by the sequential activation of Fyn, FAK, and RhoGAP (Thomason et al., 2020), and that high levels of Tns3 protein are detected in the cell body and processes of iOLs coinciding with their large enlargement, Tns3 is thus well placed to mediate integrin signaling to the actin cytoskeleton and play an active role in this large cellular remodeling. Moreover, integrin-β1, FAK/Ptk2, Fyn, p130Cas/Bcar1, and Tns3 are all highly expressed in iOLs (Figure 5—figure supplement 1). Here, using three independent approaches, we show that loss of Tns3 in iOLs reduces by half the numbers of OLs in the postnatal brain. It is therefore very likely that Tns3 act as a mediator of integrin α6β1 signaling to promote OL survival and differentiation by mediating actin cytoskeletal remodeling. Finally, through its additional ability to bind to EGFR (Cui et al., 2004), whose activation is another driver of oligodendroglial differentiation, Tns3 could also be required for mediation of signaling downstream of growth factor receptor activation in early iOLs; this could also explain the increased death in OLs lacking Tns3.

### Tns3’s role in immature oligodendrocyte survival

Programmed cell death regulates developmental oligodendrogenesis, with a large proportion of iOLs degenerating before the fourth week of postnatal life in mice (Barres et al., 1992; Trapp et al., 1997). Also in the adult mouse brain, differentiating OPCs remain in the iOL stage for roughly 2 days with many of them undergoing programmed cell death (Hughes et al., 2018), indicating that this immature stage is very dependent on survival signals. Apoptotic pathways involving BCL-2 family members have been shown to regulate this oligodendroglial programmed cell death (reviewed in Hughes and Stockton, 2021). One study has shown that the transcription factor TFEB, involved in autophagy and lysosomal biogenesis, ensures the spatial and temporal specificity of developmental myelination by promoting the expression of ER stress genes and PUMA, a pro-apoptotic factor inducing Bax-Bak-dependent programmed cell death in differentiating oligodendroglia (Sun et al., 2018). Another recent study showed that during both during homeostasis and remyelination the activity of the primary sensor of cellular stress, nuclear factor (erythroid-derived 2)-like 2 (Nrf2), induces the expression of Gsta4, a scavenger of lipid peroxidation, which in turn controls apoptosis of iOLs via the mitochondria-associated Fas-Casp8-Bid-axis (Carlström et al., 2020).

Several studies have shown that integrin-β1 signaling is required for iOL survival. Neuronal-derived signals, including neuregulin and laminin-2, are received by iOLs through integrin-β1 signaling that would enhance the function of neuroligin as a survival factor by inducing a survival-dependence switch from the phosphatidylinositol 3-kinase-Akt pathway to the mitogen-activated protein kinase (MAPK) pathway, with enhanced MAPK signaling inactivating the pro-apoptotic molecule BAD (Colognato et al., 2002; Benninger et al., 2006). Also, PDGF survival signaling in OPCs and myelin formation have been shown to depend on integrin α6β1 binding to Fyn (Colognato et al., 2004). Tensins typically reside at focal adhesions, which connect the extracellular matrix (ECM) to the cytoskeletal networks through integrins and their associated protein complexes (Kumar, 1998; Liao and Lo, 2021), with focal adhesions mediating both outside-in and inside-out signaling pathways that regulate cellular events, such as cell attachment, migration, proliferation, apoptosis, and differentiation (Liao and Lo, 2021). In this study, we show that Tns3 expression timing during oligodendrogenesis parallels that of integrin-β1, and we provided evidence of Tns3 and integrin-β1 co-localization in dotted structures resembling nascent and focal adhesion in the cytoplasm and processes of iOLs. Moreover, Tns3-deleted oligodendroglia have a 1.4-fold downregulation of *Fyn* transcripts, Src family kinase that associates with α6β1 and is required to amplify PDGF survival signaling (Colognato et al., 2004), accompanied by the upregulation of transcripts of other integrin family members normally not expressed in OPCs and iOLs (Supplementary file 1), suggesting cellular changes to compensate the integrin signaling impairment of *Tns3*-deleted cells. Remarkably, knockout mice for *integrin-α6* present a 50% reduction in brainstem MBP^+^ OLs at E18.5, just before they die at birth, accompanied by an increase in TUNEL^+^ dying OLs (Colognato et al., 2002), while conditional deletion of *integrin-β1* in iOLs by *Cnp-Cre* also leads to a 50% reduction in cerebellar OLs at P5, with a parallel increase in TUNEL^+^ dying OLs (Benninger et al., 2006). Therefore, given that *Tns3*-induced deletion in postnatal OPCs also leads to a 40–50% reduction in OLs in both gray and white matter regions of the postnatal telencephalon (this study), paralleled by a similar increase in TUNEL^+^ apoptotic oligodendroglia, we suggest that Tns3 is required for integrin-β1-mediated survival signal in iOLs. Moreover, we suggest that this would lead to cellular stress of *Tns3*-deleted differentiating oligodendroglia and to the upregulation of p53, master regulator of cellular stress and apoptosis, which has been previously been shown to be involved in the apoptosis of human OLs in the context of MS (Ladiwala et al., 1999; Wosik et al., 2003) and in the cuprizone demyelination mouse model (Li et al., 2008; Luo et al., 2021).

In summary, here we have generated powerful genetic tools allowing to assess for the first time the role of Tns3 in the CNS, showing that Tns3 protein is found at high levels in the cytoplasm and main processes of iOLs, thus constituting a new marker of this oligodendroglial stage, and demonstrated by different genetic approaches that Tns3 deletion leads to a twofold reduction in differentiating OLs, explained at least in part by their increased apoptosis due to p53 upregulation and likely the loss of integrin-β1-mediated survival signaling. Follow-up studies using these tools should unravel with more detail the molecular mechanisms mediated by Tns3 not only in iOLs during developmental myelination but also in pathological contexts such as preterm birth dysmyelination, adult demyelination in MS and glioblastoma, this last one recently associated with reduced levels of Tns3 (Chen et al., 2017).

## Materials and methods

**Key resources table:** 

Reagent type (species) or resource	Designation	Source or reference	Identifiers	Additional information
Strain, strain background (*Mus musculus*)	*Tns3* ^β*geo*^	Su Hao Lo lab (UC Davis, USA)		
Strain, strain background (*M. musculus*)	*Tn3^Flox^*	Our lab		Available as collaboration
Strain, strain background (*M. musculus*)	*Tns3^Tns3-V5^*	Our lab		Available as collaboration
Strain, strain background (*M. musculus*)	*Tns3^KO^*	Our lab		Lost in the Covid-19
Chemical compound, drug	Tamoxifen	Sigma	T5648	
Chemical compound, drug	32% PFA solution	Electron Microscopy Sciences	50-980-495	
Commercial assay or kit	Neural tissue dissociation kit (P)	Miltenyi Biotec	130-093-231	
Commercial assay or kit	Debris removal kit	Miltenyi Biotec	130-090-101	
Commercial assay or kit	Anti-PDGFRα coupled-beads	Miltenyi Biotec	130-094-543	
Commercial assay or kit	Anti-O4 coupled-beads	Miltenyi Biotec	130-096-670	
Chemical compound, drug	Normal goat serum	Eurobio	CAECHVOO-OU	
Chemical compound, drug	DAPI	Sigma-Aldrich	D9542	
Chemical compound, drug	Fluoromount-G	SouthernBiotech	15586276	
Commercial assay or kit	In Situ Cell Death detection kit	Roche	12156792910	
Chemical compound, drug	RIPA buffer	Thermo Fisher	89901	
Chemical compound, drug	Halt Protease Inhibitor Cocktail	Thermo Fisher	87786	
Commercial assay or kit	Pierce Detergent Compatible Bradford Assay Kit	Thermo Fisher	23246	
Chemical compound, drug	Bolt LDS Sample Buffer	Thermo Fisher	B0007	
Chemical compound, drug	4–12% polyacrylamide gradient gels	Thermo Fisher	NW04122BOX	
Chemical compound, drug	Bolt MOPS SDS Running Buffer	Thermo Fisher	B0001	
Chemical compound, drug	Mini Gel Tank and Blot Module Set	Thermo Fisher	NW2000	
Chemical compound, drug	Precision Plus Protein All Blue protein standards	Bio-Rad	1610373EDU	
Chemical compound, drug	Amersham Protran 0.2 μm nitrocellulose membrane	Dutscher	10600001	
Chemical compound, drug	NuPAGE Transfer Buffer	Thermo Fisher	NP0006-1	
Chemical compound, drug	Pierce ECL Western Blotting Substrate	Thermo Fisher	32109	
Chemical compound, drug	Poly-l-ornithine	Sigma	P4957	
Chemical compound, drug	DMEM/F12	Life Technologies	31331028	
Chemical compound, drug	HEPES buffer	Life Technologies	15630056	
Chemical compound, drug	Glucose	Sigma	G8769	
Chemical compound, drug	Penicillin/streptomycin	Life Technologies	15140122	
Chemical compound, drug	N2 supplement	Life Technologies	17502048	
Chemical compound, drug	B27 supplement	Life Technologies	17504044	
Chemical compound, drug	EGF	PeproTech	AF-100-15	
Chemical compound, drug	FGF-basic	PeproTech	100-18B	
Chemical compound, drug	PDGF-AA	PeproTech	100-13A	
Chemical compound, drug	Insulin	Sigma	I6634	
Chemical compound, drug	iDeal ChIPseq kit for Transcription Factors	Diagenode	C01010055	
Antibody	Anti-Tensin1 (rabbit)	Su Hao Lo, UC Davis		1:100
Antibody	Anti-Tensin2 (rabbit)	Su Hao Lo, UC Davis		1:100
Antibody	Anti-Tensin3 (rabbit)	Sassan Hafizi, University of Portsmouth		1:1000
Antibody	Anti-Tensin3 (rabbit)	Millipore	AB229	1:500
Antibody	Anti-Tensin3 (rabbit)	Thermo Fisher	PA5-116022	1:1000
Antibody	Anti-Tensin3 (mouse monoclonal)	Santa Cruz Biotech	sc-376367	1:500
Antibody	Anti-Tns3 (rabbit) (C-terminal)	Sigma-Aldrich	SAB4200205	1:200
Antibody	Anti-Tns3 (rabbit) (TN-17)	Sigma-Aldrich	SAB4200416	1:400
Antibody	Anti-V5 tag (rabbit)	Millipore	AB3792	1:2000
Antibody	Anti-V5 tag (mouse monoclonal)	Invitrogen	R960-25	1:1000
Antibody	Anti-PDGFRα (rat)	BD Biosciences	558774	1:250
Antibody	Anti-Olig1 (mouse monoclonal)	NeuroMab	75-180	1:1000
Antibody	Anti-Olig2 (mouse monoclonal)	Millipore	MABN50	1:500
Antibody	Anti-CNPase (mouse monoclonal)	Millipore	MAB326R	1:250
Antibody	Anti-IP3 receptor 2 (rabbit) (Itpr2)	Millipore	AB3000	1:40
Antibody	Anti-Nkx2.2 (mouse polyclonal)	Developmental Studies Hybridoma Bank		1:4
Antibody	Anti-CC1 (mouse monoclonal) (Quaking 7)	Calbiochem	OP80	1:100
Antibody	Anti-MOG (mouse monoclonal)	ICM, Paris Hybridoma	AA3	1:20
Antibody	Anti-Opalin (mouse monoclonal)	Santa Cruz Biotech	sc-374490	1:500
Antibody	Anti-GFP (chicken polyclonal)	Aves Labs	GFP-1020	1:1000
Antibody	Anti-GFP (rabbit)	Life Technologies	A6455	1:1000
Antibody	Anti-MCM2 (mouse)(BM28)	BD Biosciences	610701	1:500
Antibody	Anti-p53 (rabbit)	Leica	P53-CM5P-L	1:500
Antibody	Anti-F4/80 (rat)	Abd Serotec	MCA497	1:100
Antibody	Anti-beta-galactosidase (mouse monoclonal)	Promega	Z3783	1:1000
Antibody	HRP-conjugated anti-rabbit	Bio-Rad	1706515	1:5000
Antibody	HRP-conjugated anti-mouse	Sigma-Aldrich	NA931-1ML	1:5000
Antibody	HRP-conjugated anti-rat	Thermo Fisher Scientific	A10549	1:5000
Antibody	Anti-H3K4me3 (rabbit)	Active Motif	39060	
Antibody	Anti-H3K27Ac (rabbit)	Active Motif	39034	
Antibody	Anti-H3K27me3 (mouse)	Abcam	ab6002	
Antibody	Anti-H3K4me1 (rabbit)	Ozyme	5326T	
Plasmid	*gRNA-pCMV-Cas9-2A-GFP*	Addgene		
Plasmid	*gRNA-pCMV-Cas9-2A-Puro*	Addgene		
Plasmid	*Tol2-gRNA-pCMV-Cas9-2A-GFP*	Our lab		
Plasmid	*Tol2-gRNA-pCMV-Cas9-2A-Puro*	Our lab		
Plasmid	*Tol2-gRNA-pCAG-Cas9-2A-GFP*	Our lab		
Plasmid	*Tol2-gRNA-pCAG-Cas9-2A-Puro*	Our lab		
Plasmid	*Tol2-Tns3gRNA2-pCAG-Cas9-2A-GFP*	Our lab		

### Animals

All animal procedures were performed according to the guidelines and regulations of the Inserm ethical committees (authorization #A75-13-19) and animal experimentation license A75-17-72 (CP). Both males and females were included in the study. Mice were maintained in standard conditions with food and water ad libitum in the ICM animal facilities. *Tensin3* gene trap mouse line (*Tns3^βgeo^*) was from Su Hao Lo lab (UC Davis, USA). Mice used for ChIP-seq analysis were wild-type Swiss obtained from Janvier Labs. *Tns3^flox^* were crossed with *Pdgfra-CreER^T^* (Kang et al., 2010) and *Rosa26^stop-floxed-YFP^* mice to generate *Tns3^flox^; Pdgfra-CreER^T^; Rosa26^stop-floxed-YFP^* mice line. *Pdgfra-CreER^T^; Rosa26^stop-floxed-YFP^* mice were used as controls.

### Generation of *Tns3^Tns3-V5^* knock-in mice

*Tns3^Tns3-V5^* mice were generated at the Curie Institute mouse facility. Briefly, the Cas9 protein, the crRNA, the tracrRNA, and an ssODN targeting vector for the *Tns3* gene had been microinjected into a mouse egg cell, which was transplanted into a C57BL/6J-BALB/cJ female surrogate. Pups presenting HDR insertion of the V5 tag were selected after genotyping.

### Generation of *Tns3^4del^* and *Tns3^14del^* knockout mice

*Tns3^KO^* mice were generated at the ICM mice facility. Briefly, the Cas9 protein, the crRNA, the tracrRNA, and a targeting vector for the *Tns3* gene had been microinjected into a mouse egg cell transplanted into a C57BL/6J female surrogate. Pups with NHEJ mutations inducing a gene frameshift were selected after genotyping and Sanger sequencing verification. Finally, only two lines containing indels of 4 and 14 nucleotide deletions were maintained and studied.

### Generation of *Tns3^Flox^* mice and tamoxifen administration

We designed a *Tns3* conditional knockout allele by flanking with LoxP sites exon 9 (LoxP-Exon9-LoxP; Figure 4—figure supplement 1a). In this *Tns3*-floxed allele (*Tns3^flox^*), Cre-mediated recombination induces a transcription frame shift translated into an early stop codon, leading to a putative small peptide of 109 aa instead of the full-length Tns3 protein (1442 aa; Figure 4—figure supplement 1b and c). *Tns3^flox^* mice were generated at the Transgenic Core Facility of the University of Copenhagen. The repair template contained homology arms of 771 bp and 759 bp length and *loxP* sequences flanking exon 9, was synthesized by Invitrogen, and verified by Sanger sequencing. Two gRNAs were designed at the Transgenic Core Facility that target a DNA sequence in the proximity of each *loxP* site. The gRNAs were designed in a fashion where the insertion of the *loxP* disrupts the targeting site, thus preventing retargeting of the repaired DNA. Mouse embryonic stem cell (mESC) method was used for the generation of this mouse model by transfecting ESCs with the repair construct (dsDNA) together with two plasmids – each containing each gRNA. Identification of the positive mESC clones was done via a combination of a PCR genotyping and Sanger sequencing confirmation. Mouse ESCs were transfected with a plasmid expressing *Cas9, GFP*, and gRNAs flanking *Tns3* exon 9, and a *Tns3*-floxed targeting vector (Figure 4—figure supplement 1d), in order to induce CRISPR/Cas9-mediated homologous recombination. After verifying the presence of *Tns3*-floxed allele in *Tns3* locus by Sanger sequencing, positive ESC clones were injected into blastocysts to generate *Tns3*-floxed (*Tns3^flox^*) mice.

Tamoxifen (Sigma, T5648) was dissolved in corn oil (Sigma, C-8267) and injected subcutaneously at 20 mg/ml concentration at P7 (30 µl) in *Ctrl* and *Tns3-iKO* animals. Brains were then collected at P21.

### Postnatal electroporation

Postnatal brain electroporation (Boutin et al., 2008) was adapted to target the dorsal SVZ. Briefly, postnatal day 1 (P1) pups were cryoanesthetized for 2 min on ice and 1.5 μl of plasmid was injected into their left ventricle using a glass capillary. Plasmids were injected at a concentration of 2–2.5 μg/μl. Electrodes (Nepagene CUY650P10) coated with highly conductive gel (Signagel, signa250) were positioned in the dorsoventral axis with the positive pole dorsal. Five electric pulses of 100 V, 50 ms pulse ON, 850 ms pulse OFF were applied using a Nepagene CUY21-SC electroporator. Pups were immediately warmed up in a heating chamber and brought to their cages at the end of the experiment.

### Demyelinating lesions

Before surgery, adult (2–3 months) WT mice were weighed, and an analgesic (buprenorphine, 30 mg/g) was administered to prevent postsurgical pain. The mice were anesthetized by induction of isoflurane (ISO-VET). Ocrygel (Tvm) was put on their eye to prevent dryness and lidocaine in cream (Anesderm 5%) was put on the ear bars to prevent pain. After cutting of the skin, a few drops of liquid lidocaine were put to prevent pain. Focal demyelinating lesions were induced by stereotaxic injection of 1 µl of lysolecithin solution (LPC, Sigma, 1% in 0.9% NaCl) into the corpus callosum (CC; at coordinates: 1 mm lateral, 1.3 mm rostral to bregma, 1.7 mm deep) using a glass-capillary connected to a 10 µl Hamilton syringe. Animals were left to recover in a warm chamber before being returned into their housing cages.

### Tissue processing

Postnatal mice were transcardially perfused with 15 ml (P14) or 25 ml (>P21) of 2% PFA freshly prepared from 32% PFA solution (Electron Microscopy Sciences, 50-980-495). Perfused brains were dissected out, dehydrated in 10% sucrose, followed by 20% sucrose overnight, and embedded in OCT (BDH) before freezing and sectioning (16 µm thickness) in a sagittal plane with a cryostat microtome (Leica).

### Magnetic-assisted cell sorting (MACS)

Dissociation of cortex and corpus callosum from mice brain was done using neural tissue dissociation kit (P) (Miltenyi Biotec; ref 130-093-231). Briefly, cortices were dissected from P7, P12, P14, or P21 mice and dissociated using a MACS dissociator (Miltenyi Biotec; ref 130-096-427) followed by filtration through a 70 μm cell strainer (Smartstainer; Miltenyi Biotec; ref 130-098-462). Myelin residues were eliminated from P12, P14, and P21 mice cortices during an additional step using the debris removal kit (Miltenyi Biotec; ref 130-090-101). Cells were suspended in a 0.5% NGS solution, then incubated with anti-PDGFRα or anti-O4 coupled-beads (Miltenyi Biotec; ref 130-094-543 and 130-096-670). Unbound bead-coupled antibodies were washed away by centrifugation, leaving bound cells that were sorted using MultiMACS Cell24 Separator Plus (Miltenyi Biotec; ref 130-098-637). Sorted cells were either plated in culture plates for in vitro cell study or centrifuged at 1200 rpm and used for Western blot analysis or ChIP-seq.

### Immunofluorescence staining and microscopy

Postnatal mouse brain cryosections were dried for 20 min at room temperature (RT), before adding the blocking solution (10% normal goat serum [NGS, Eurobio, CAECHVOO-OU] and 0.1% Triton X-100 in PBS) for 1 hr at RT. Primary antibodies were diluted (dilutions indicated in the Key ressource table) in the same blocking solution and incubated on the slices overnight at 4°C. After washing with 0.05% Triton X-100 in PBS, sections were incubated with secondary antibodies conjugated to Alexa Fluor-488, Alexa Fluor-594, and Alexa Fluor-647 (Thermo, 1:1000). Finally, cell nuclei were labeled with DAPI (1/10,000, Sigma-Aldrich, D9542-10MG), and slices mounted in Fluoromount-G (SouthernBiotech, Inc 15586276).

In Situ Cell Death detection kit (Roche, 12156792910) was used to do TUNEL experiment on P21 mouse brains. Briefly, tissues were processed as mentioned above with anti-GFP and anti-CC1 and fixed in fixation solution for 20 min at RT. After washing, slices were permeabilized for 2 min in permeabilization solution (0.1% Triton X-100; 0.1% sodium citrate) and TUNEL reaction mixture was put on samples for 1 hr at 37°C. Tissues were then mounted in Fluoromount-G.

Fixed coverslips were blocked in blocking solution (10% NGS [Eurobio, CAECHVOO-OU] and 0.1% Triton X-100 in PBS) for 30 min at RT, incubated in the primary antibodies for 45 min at RT, and washed three times in 1× PBS. Secondary antibodies were applied for 45 min at RT and washed three times in 1× PBS. Coverslips were then incubated with DAPI solution for 5 min at RT. A final washing was done before mounting the coverslips on slides to be visualized under the microscope.

Immunofluorescence was visualized with Zeiss Axio Imager.M2 microscope with Zeiss Apotome system. Pictures were taken as stacks of 5–10 µm with 0.5 µm between sections. Image acquisition and processing were achieved by ZEN Microscopy and Imaging Software. Z-projections and orthogonal projections were done in ImageJ and processed with Adobe Photoshop. Figures were created using Adobe Illustrator.

### Western blot

Proteins from MACsorted cells were extracted during 30 min at 4°C in RIPA buffer (Thermo Fisher; 50 µl per million cells, 89901) supplemented with Halt Protease Inhibitor Cocktail (100×; Thermo Fisher, 87786). Protein concentration in the supernatant was estimated using the Pierce Detergent Compatible Bradford Assay Kit (Thermo Fisher, 23246). For each Western blot, we used 50 µg of proteins denaturated for 10 min at 95°C with added β-mercaptoethanol (from 24× stock) and Bolt LDS Sample Buffer (4×) (Thermo Fisher, B0007). Sodium dodecyl sulfate-polyacrylamide gel electrophoresis (SDS-PAGE) was performed using precast 4–12% polyacrylamide gradient gels (Thermo Fisher, NW04122BOX), submerged at 4°C in Bolt MOPS SDS Running Buffer (Thermo Fisher, B0001) using Mini Gel Tank and Blot Module Set (Thermo Fisher, NW2000). Precision Plus Protein All Blue protein standards (Bio-Rad, 1610373EDU) were run alongside the samples as a protein migration control. Proteins were separated for 90 min at 90 V, after which gels were transferred onto Amersham Protran 0.2 µm nitrocellulose membrane (Dutscher, 10600001) immersed at 4°C in NuPAGE Transfer Buffer (Thermo Fisher, NP0006-1) for 90 min at 60 V. Following transfer, membranes were incubated for 1 hr in TBS-T, 10% dry milk to aid blocking of nonspecific binding by the antibodies. Primary antibodies diluted in TBS-T were incubated with the membrane overnight at 4°C with shaking. After three washes in TBS-T, membranes were incubated with HRP-conjugated secondary antibodies diluted in TBS-T for 1 hr at 4°C with shaking, then developed using Pierce ECL Western Blotting Substrate (Thermo Fisher, 32109) and imaged with the ChemiDoc Touch Imaging System (Bio-Rad, 1708370). Western blot detection of actin was performed as loading control.

### Videomicroscopy

Tamoxifen was administered to P5 *Tns3^flox^; Pdgfrα-CreER^T^; Rosa26^stop-floxed-YFP^* and *Tns3^flox^; Pdgfrα-WT; Rosa26^stop-floxed-YFP^* littermates. Brains were dissected out at P7 in order to MACSort OPCs using an anti-PDGFRα antibody coupled to magnetic beads. OPCs were plated in poly-l-ornithine (Sigma, P4957)-coated µ-Slide 8 Well Glass Bottom slide (ibidi, 80827) at 40,000 cells/mm^2^ in OPC proliferative medium: DMEM/F12 (Life Technologies, 31331028), 5 mM HEPES buffer (Life Technologies, 15630056), 0.6% glucose (Sigma, G8769), 1× penicillin/streptomycin (Life Technologies, 15140122), N2 supplement (Life Technologies, 17502048), B27 supplement (Life Technologies, 17504044), 20 ng/µl EGF (PeproTech, AF-100-15), 10 ng/µl FGF-basic (PeproTech, 100-18B), 10 ng/µl PDGF-AA (PeproTech, 100-13A), and 20 μg/ml insulin (Sigma, I6634). After 3 days of proliferation, medium was replaced by growth factor-depleted medium. Cell differentiation was tracked for 3 days using time-lapse video recording. Cells were put in to a videomicroscope (Zeiss AxioObserver 7, provided by ICM-quant and CELIS facilities) with a humidified incubator at 37°C with a constant 5% CO_2_ supply. Images for both FITC and bright field were acquired every 10 min.

### Chromatin immunoprecipitation (ChIP)

ChIP-seq assays were performed as described previously (Marie et al., 2018) using iDeal ChIP-seq kit for Transcription Factors (Diagenode, C01010055). Briefly, O4^+^ MACSorted cells were fixed in 1% formaldehyde (EMS, 15714) for 10 min at RT and the reaction was quenched with 125 mM glycine for 5 min at RT. Lysates were sonicated with a Bioruptor Pico sonicator (Diagenode, total time 8 min) and 4 μg of antibodies were added to sheared chromatin (from 4 million cells for Olig2 and from 1 million cells for histone marks) and incubated at 4°C overnight on 10 rpm rotation. Antibodies used were mouse anti-Olig2 antibody (Millipore, MABN50), rabbit anti-H3K4me3 antibody (Active Motif, 39060), rabbit anti-H3K27Ac antibody (Active Motif, 39034), rabbit anti-H3K4me1 antibody (Ozyme, 5326T), and mouse anti-H3K27me3 antibody (Abcam, ab6002). Mock (rabbit IgG) was used as negative control. Chromatin-protein complexes were immunoprecipitated with protein A/G magnetic beads and washed sequentially according to the manufacturer (Diagenode, C01010055). DNA fragments were then purified using IPure beads v2 (Diagenode, C01010055). Input (non-immunoprecipitated chromatin) was used as control in each individual experiment. The ChIP-seq libraries were prepared using Illumina TruSeq ChIP preparation kit and sequenced with Illumina NextSeq 500 platform.

### ChIP-seq analysis

All ChIP-seq analyses were done using the Galaxy Project (https://usegalaxy.org/). Reads were trimmed using Cutadapt (--max-n 4) and Trimmomatic (TRAILING 1; SLIDINGWINDOW 4 and cutoff 20; LEADING 20; MINLEN 50) and mapped using Bowtie2 onto mm10 mouse reference genome (-X 600; -k 2; --sensitive). PCR-derived duplicates were removed using PICARD MarkDuplicates. Bigwig files were generated with bamCoverage (binsize = 1). Peak calling was performed using MACS2 callpeak with Input as control and with options: --qvalue 0.05; --nomodel; --keep-dup 1; --broad (only for histone marks). Blacklisted regions were then removed using bedtools Intersect intervals.

Visualization of coverage and peaks was done using IGV (Robinson et al., 2011; http://software.broadinstitute.org/software/igv/home). Intersection and analysis of bound genes were done using Genomatix (https://www.genomatix.de/). Chd7, Chd8, and Mock ChIP-seq datasets are from Marie et al., 2018. Heatmap was done using R (4.0) and pheatmap package. GO analysis was done using Enrichr GO Biological Process 2021.

Two replicates were done for Olig2, with one of them of better quality (53,960 peaks for replicate 1 and 14,242 peaks for replicate 2). Only the peaks found in both replicates (6781) and the peaks from replicate 1 found in regulatory elements (13,948) were considered (16,578 in total). Three replicates were done for H3K4me3, two replicates were done for H3K27me3 and one replicate was done for H3K27Ac and H3K4me1. Intersection of these datasets was done using bedtools Intersect intervals.

Peaks overlapping with regions between 1000 bp upstream of transcription start site (TSS) and 10 bp downstream of TSS were identified as ‘promoters’ (Genomatix). ‘Active promoters’ were marked by peaks for H3K4me3 and H3K27Ac; ‘Repressed promoters’ by peaks for H3K27me3 and no active marks; ‘Poised promoters’ by peaks for H3K4me1 and no active or repressed marks. Regions outside promoters containing histone marks were considered as ‘enhancers.’ ‘Active enhancers’ were marked by peaks for H3K27Ac; ‘Repressed enhancers’ by peaks for H3K27me3 and no active marks; ‘Poised enhancers’ by peaks for H3K4me1 and no active or repressed marks. Genes were considered associated if the peaks were present in the promoter or within a range of 100 kb from the middle of the promoter and the gene expression was medium to high (‘active’), low (‘poised’), or not (‘repressed’) expressed (based on control RNA-seq dataset in GSE116601).

### RNA-seq analysis

Raw data were downloaded from GEO datasets GSE107919 and GSE116601 and processed through the Galaxy Project (https://usegalaxy.org/) using RNAstar for alignment on mm10 reference genome and featureCounts to obtain counts. Count per million (CPM), FPKM, and statistical analysis were done with R (4.0) using edgeR quasi-likelihood pipeline. Using control RNA-seq dataset in GSE116601, genes were classified based on their expression as not (below first quartile), low (between first quartile and mean), medium (between mean and third quartile), and high (above third quartile).

### scRNA-seq analysis

For mouse oligodendroglial cell analyses, counts per gene were downloaded from GEO datasets GSE75330 and GSE95194, and processed in R (4.0) using the following packages using de R-scripts deposited in https://github.com/ParrasLab/Tns3_paper_eLife_2022; ParrasLab, 2022 and summarized here: *Seurat* (3.0) for data processing, *sctransform* for normalization, and *ggplot2* for graphical plots. Seurat objects were first generated for each dataset independently using *CreateSeuratObject* function (min.cells = 5, min.features = 100). Cell neighbors and clusters were found using *FindNeighbors* (dims = 1:30) and *FindClusters* (resolution = 0.4) functions. Clusters were manually annotated based on the top 50 markers obtained by the *FindAllMarkers*, adopting mainly the nomenclature from Marques et al., 2016. Using the *subset* function, we selected only the clusters containing neural progenitors and oligodendroglia cells. Using the *merge* function, we combined both oligodendroglial datasets into a single Seurat object (OLgliaDevPost). The new object was subjected to *NormalizeData*, *FindVariableFeatures*, *ScaleData*, *RunPCA*, and *RunUMAP* functions with default parameters. Different OPC clusters were fused into a single one keeping apart the cycling OPC cluster. For DimPlots and dot plots, clusters were ordered by stages of oligodendrogenesis from NSCs to myelinating OLs.

### *Tns3^βgeo^* mice analysis

To explore the role of Tns3 in OL differentiation, we first analyzed a *Tns3* gene trap mouse line (*Tns3^βgeo^*) previously studied outside the CNS (Chiang et al., 2005), where the *βgeo* cassette is inserted after *Tns3* exon 4 (Figure 3—figure supplement 1a) driving *LacZ* transcription and by inserting a stop poly-A sequence, predicted to be a *Tns3* loss-of-function mutation. Despite the original report of postnatal growth retardation in *Tns3^βgeo/βgeo^* mice, these mice were kept in homozygosity for several generations in C57BL/6 genetic background (Su-Hao Lo, UC Davis). We thus analyzed the impact in oligodendrogenesis in the postnatal brain of *Tns3^βgeo^* animals. We first immunodetected βgalactosidase in OLs (Olig2^+^/CC1^+^ and Olig2^+^/PDGFRα− cells) of *Tns3^βgeo^* postnatal brains at P21 (Figure 3—figure supplement 1b and c), paralleling our characterization of Tns3 expression with V5 and Tns3 antibodies. We then quantified the density of PDGFRα^+^ OPCs or CC1^+^ OLs in *Tns3^βgeo/βgeo^* and *Tns3^βgeo/+^* littermates at P21, finding similar number of OPCs and OLs in two main white matter areas (corpus callosum and fimbria; Figure 3—figure supplement 1d, d', e). Moreover, quantification of three different stages of OL differentiation (iOL1, iOL2, and mOL) by Olig2/CC1/Olig1 immunofluorescence did not reveal changes in the rate of OL differentiation (proportion of each stage) in *Tns3^βgeo/βgeo^* mice compared to control littermates (Figure 3—figure supplement 1f, f', g). We verified the homozygosity of *Tns3^βgeo^* allele in *Tns3^βgeo/βgeo^* mice by PCR amplification from genomic DNA of P21 brains finding that primers recognizing intron 4 and βgeo only produced PCR amplicons in *Tns3^βgeo/βgeo^* mice but not when using intron 4 flanking primers that only produced PCR amplicons in wild-type mice (Figure 3—figure supplement 1h). We then checked for *Tns3* full-length transcripts using cDNA generated from P21 brains, and to our surprise, primers flanking exons 17 and 31 were similarly amplified from cDNA of *Tns3^βgeo/βgeo^* and wild-type brains (Figure 3—figure supplement 1i), suggesting that in the brain of *Tns3^βgeo/βgeo^* mice *Tns3* full-length transcripts coding for Tns3 protein are still produced. Altogether, these results suggested that, unlike in other tissues (Chiang et al., 2005), the *Tns3^βgeo^* allele does not lead to *Tns3* loss of function in the brain, likely through the generation of alternative spliced *Tns3* variants, and is thus not suitable for assessing *Tns3* function in the CNS.

### Analyses of *TNS3* alleles in the human population based in gnomAD project

To assess whether TNS3 is potentially required during human development, we explore for the presence of *TNS3* gene variants in the human population using the gnomAD database containing 125,748 exomes and 15,708 whole-genome sequences from unrelated individuals (Karczewski et al., 2020; Lek et al., 2016). Homozygous predicted loss-of-function (pLoF) alleles of *TNS3* were not found, and heterozygous pLoF were greatly below the expected frequency (0.1 observed/expected ratio, with 90% CI of 0.05–0.19; and LOEUF of 0.19; Figure 3—figure supplement 2a; https://gnomad.broadinstitute.org), meaning that heterozygous loss-of-function variants of *TNS3* cause ~80% developmental mortality, a rate similarly high to key neurodevelopmental genes such as *SOX10* (LOEUF = 0.21; Figure 3—figure supplement 2b), *CHD7* (LOEUF = 0.08; Figure 3—figure supplement 2c), and *CHD8* (LOEUF = 0.08; Figure 3—figure supplement 2d), contrary to less broadly required factors such as *NKX2-2* (LOEUF = 0.67; Figure 3—figure supplement 2e) and *OLIG1* (LOEUF = 1.08; Figure 3—figure supplement 2f). Therefore, *TNS3* loss-of-function variants are badly tolerated in both mouse and human development.

### CRISPR/Cas9 tools development

CRISPOR software (http://crispor.tefor.net/) was used to design gRNAs with predicted cutting efficiency and minimal off-target and PCR amplification primers. The validation of *Tns3*-targeting CRISPR/Cas9 system was performed in 3T3 cells by transfection with Lipofectamine 3000 of *PX459* plasmids containing four different sgRNA sequences. After 2 days incubation, puromycin was added to medium for 4 days allowing survival of cells containing the *PX459* plasmid. Three days after proliferation in fresh medium without puromycin, DNA was extracted using DNeasy blood & tissue kit (QIAGEN). The target DNA for 5′ *Tns3* region was amplified by PCR using primers with the following sequences: forward: 5′-AGG TGG CCT TCA GCT CAGT-3′, reverse: 5′-GCT ATC ATC CCC ACT CAC CA-3′; annealing temperature of 64°C, with the PCR product expected to be 326 bp. DNA from 3′ *Tns3* target region was amplified using primer with the following sequences: forward: 5′-CCA GTC AGT GGT GAC ATT GTTT-3′, reverse: 5′-ACT GTT CCC AGG TTG CTA TCAT-3′, annealing temperature of 58°C, with the PCR product expected to be 419 bp. Cutting efficiency of sgRNA was verified by T7 endonuclease I, following the beta protocol of IDTE synthetic biology for amplification of genomic DNA and detecting mutations (Figure 3—figure supplement 3c), and using PAGE (Figure 3—figure supplement 3d). In order to generate plasmids that will insert CRISPR tools into the genome of the transfected cells and lead to permanent expression of the targeting tools, the *PX458* (GFP) or *PX459* (Puromycin) plasmids were subcloned into a *Tol2*-containing sequence backbone (obtained from *Tol2-mCherry*-expressing plasmid kindly provided by Jean Livet, Institut de la Vision, Paris). Finally, to induce more robust expression of the Cas9 protein and reporter genes, we substitute the CMV promoter for the stronger CAG promoter.

### *Tns3^4del^* and *Tns3^14del^* mice generation by CRISPR and analyses

To generate new *Tns3* knockout mouse using CRISPR/Cas9 technology by introducing loss-of-function mutations (indels) at the beginning of *Tns3* full-length coding sequence. We generated CRISPR integrative plasmids (Figure 3—figure supplement 3a) driving Cas9 expression and gRNAs targeting *Tns3* exon 6 at the levels of the first coding ATG using as control plasmids without the *Tns3*-targeting sequence of the gRNA. Strong cutting efficiency of two gRNAs was validated by lipofection of neural progenitors (Figure 3—figure supplement 3b–f). We then used these optimized tools to induce CRISPR-mediated *Tns3* mutations in mouse zygotes, generating and characterizing two mouse lines having small deletions (4-deletion and 14-deletion) after the first coding ATG of *Tns3* (Figure 3—figure supplement 1j), expected to cause frame shifts leading to *Tns3* loss of function. Remarkably, homozygous animals were found in reduced numbers compared to Mendelian ratios with many of them dying during embryonic development (Figure 3—figure supplement 1k) with most homozygous animals showing major growth retardation by the second postnatal week compared with their littermates (Figure 3—figure supplement 1l), similar to the original report of *Tns3^βgeo^* mice (Chiang et al., 2005). Furthermore, we could still immunodetect Tns3 protein in CC1^+^ OLs of these homozygous mice at P21 with at least two different Tns3 antibodies (Figure 3—figure supplement 1m and n) and detect *Tns3* exons corresponding to *Tns3* full-length transcript by qPCR (Figure 3—figure supplement 1o). Further analysis of these mice was prevented by the Covid-19 lockdown, leading to the loss of these *Tns3* knockout mouse lines. Altogether, these results suggest that mice carrying constitutive *Tns3* loss-of-function mutations seems to escape the full *Tns3* loss of function in the brain by generating alternative spliced variants containing the main *Tns3* full-length exons, and thus we considered these animals not suitable to study *Tns3* function in oligodendrogenesis.

### RNA sequencing and analysis

Cortices from 3 to 4 animals for each group were dissected and frozen in liquid nitrogen for further processing. Total RNA was isolated with the TRIzol Reagent protocol (Thermo Fisher) and RNeasy Mini Kit (QIAGEN) according to the instructions of the provider. The RNA-seq libraries were prepared using either the NEBNext Ultra II Directional RNA Library Prep Kit (NEB) and sequenced with the NovaSeq 6000 platform (Illumina, 32 * 106 100-bp pair-end reads per sample). Quality of raw data was evaluated with FastQC. Poor quality sequences were trimmed or removed with fastp tool, with default parameters, to retain only good quality paired reads. Illumina DRAGEN bio-IT Plateform (v3.6.3) was used for mapping on mm10 reference genome and quantification with gencode vM25 annotation gtf file. Library orientation, library composition, and coverage along transcripts were checked with Picard tools. The following analysis was conducted with R software. Data were normalized with edgeR (v3.28.0) bioconductor packages prior to differential analysis with glm framework likelihood ratio test from edgeR package workflow. Multiple hypothesis-adjusted p-values were calculated with the Benjamini–Hochberg procedure to control FDR. For the differential expression analyses, low-expressed genes were filtered, sex was used as covariable (when relevant), and the cutoffs applied were FDR < 0.05. Finally, GO enrichment analysis of biological processes of the DEGs was conducted with clusterProfiler R package (v3.14.3).

### Statistical analysis

Experimental data is the result of optimization and analyses of several experiments done for each section of the study. Biological replicates (one sample comes from one animal) were used. Some quantifications concerning comparisons between *Tns3* loss-of-function genotypes and controls were done blindly.

Statistical parameters including the exact value of n, the definition of center, dispersion, and precision measures (mean ± SEM) and statistical significance are reported in the figures, figure legends, and Source data 1. ‘n’ represents the number of animals in histological studies and number of samples in RNA-seq, ChIP-seq, and ATAC-seq studies. Data distribution was assumed to be normal, but this was not formally tested. Statistical significance was determined using two-tailed Student’s *t*-tests. One-way ANOVA test was performed for multiple comparisons or pairwise comparisons following Turkey’s ranking tests when comparing multiple groups. Data are judged to be statistically significant when p<0.05. In figures, asterisks denote statistical significance as calculated by Student’s *t*-test (*p<0.05; **,p<0.01; ***p<0.001). No statistical methods were used to predetermine sample sizes, but our sample sizes are similar to those generally employed in the field to balance experimental robustness with the 3R rule for animal experimentation. Quantifications were performed from at least three independent experiments. No randomization was used to collect all the data, but they were quantified blindly. Statistical analysis was performed in Prism software.

### Data resources

Raw data files generated in this study have been deposited in the NCBI Gene Expression Omnibus under accession number GEO GSE203295 (https://www.ncbi.nlm.nih.gov/geo/query/acc.cgi?acc=GSE203293).

R-scripts used to treat RNA-seq data are deposited in GitHub (https://github.com/ParrasLab/Tns3_paper_eLife_2022, copy archived at swh:1:rev:fa63c93277571bd0fe113242e4929ea5a1957fad; ParrasLab, 2022).

### Contact for reagent and resource sharing

Further information and reasonable requests for reagents may be directed to and fulfilled by the corresponding author Carlos Parras (carlos.parras@icm-institute.org).

## Data Availability

Sequencing data have been deposited in GEO under accession code GSE203295. The following dataset was generated: MerourE MarieC ParrasC 2022Transient regulation of focal adhesion via Tensin3 is required for nascent oligodendrocyte differentiationNCBI Gene Expression OmnibusGSE20329510.7554/eLife.80273PMC959616336214451 The following previously published datasets were used: MarquesS Castello-BrancoG 2016scRNA-seq postnatal oligodendrogliaNCBI Gene Expression OmnibusGSE75330 MarquesS Castello-BrancoG 2018scRNA-seq developmental oligodendrogliaNCBI Gene Expression OmnibusGSE95194 MillerKJ 2021snRNA-seq human midgestation cerbellumdbGaPphs001908.v2.p1 ZackDJ ChamlingX 2021scRNA-seq from iPSC-derived Human Oligodendrocyte Progenitor CellsNCBI Gene Expression OmnibusGSE146373

## Additional files

MDAR checklist

Source data 1. Statistics summary table.

Supplementary file 1. Tables containing Olig2/Chd7/Chd8 ChIP-seq analyses and Tns3-iKO RNAseq analyses.
