# Supplementary material for: Transient regulation of focal adhesion via Tensin3 is required for nascent oligodendrocyte differentiation
Source: eLife. 2022 Oct 10;11:e80273. doi: 10.7554/eLife.80273 (PMC9596163; doi:10.7554/eLife.80273)

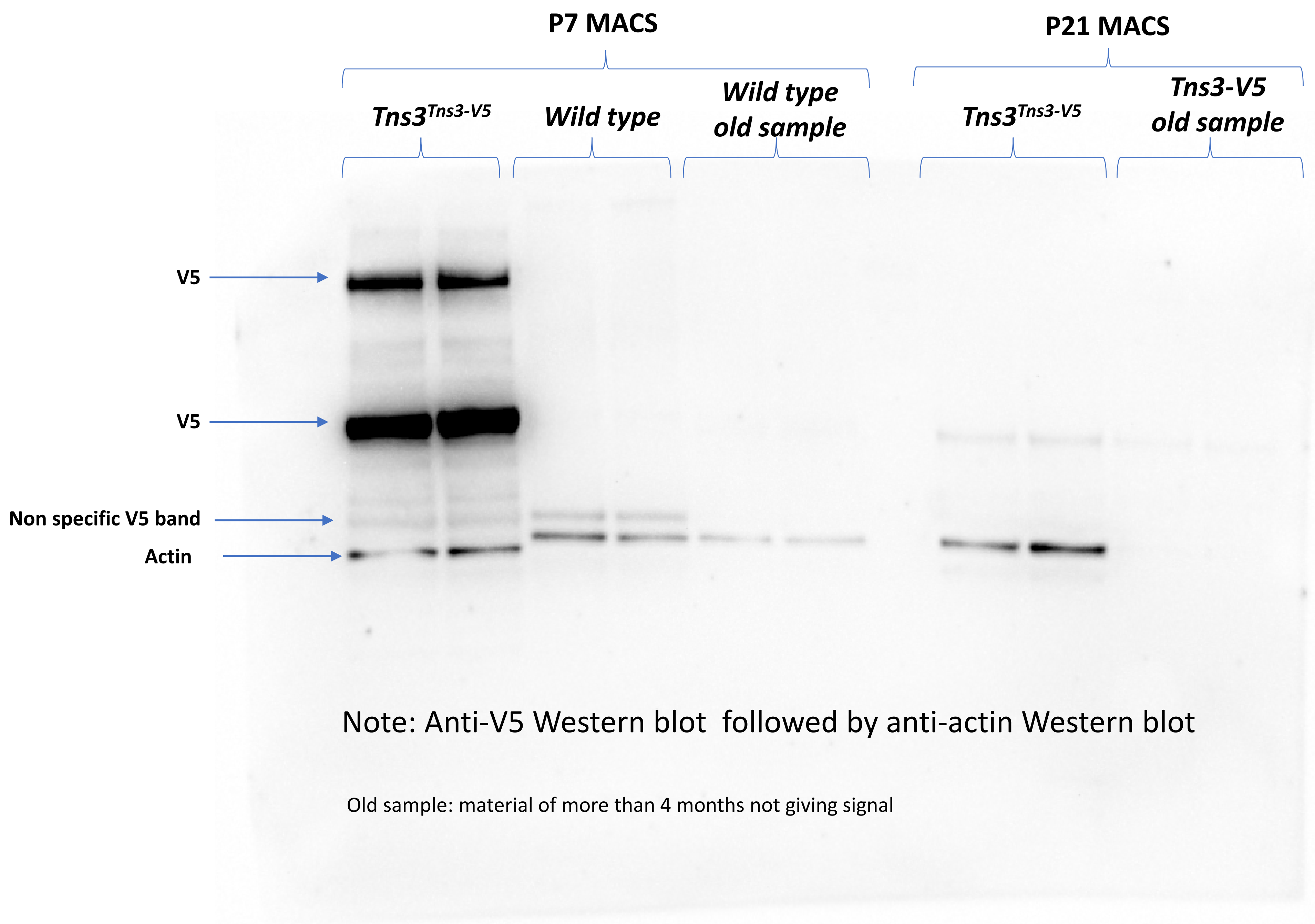
P7 MACS
P21 MACS
Tns3Tns3-V5
Wild type
Wild type old sample
Tns3Tns3-V5
Tns3-V5 old sample
V5
V5
Non specific V5 band
Actin
Note: Anti-V5 Western blot followed by anti-actin Western blot
Old sample: material of more than 4 months not giving signal

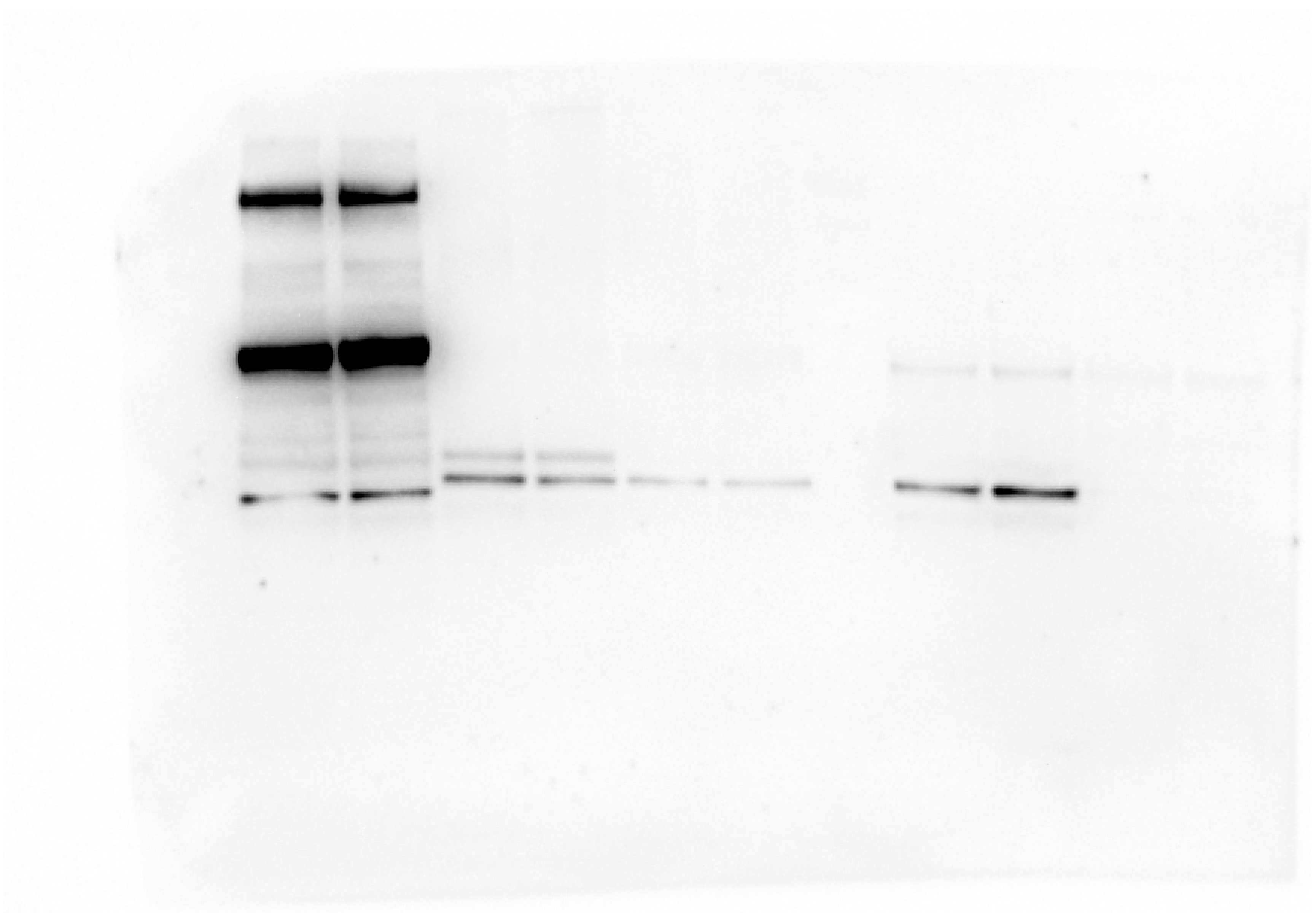

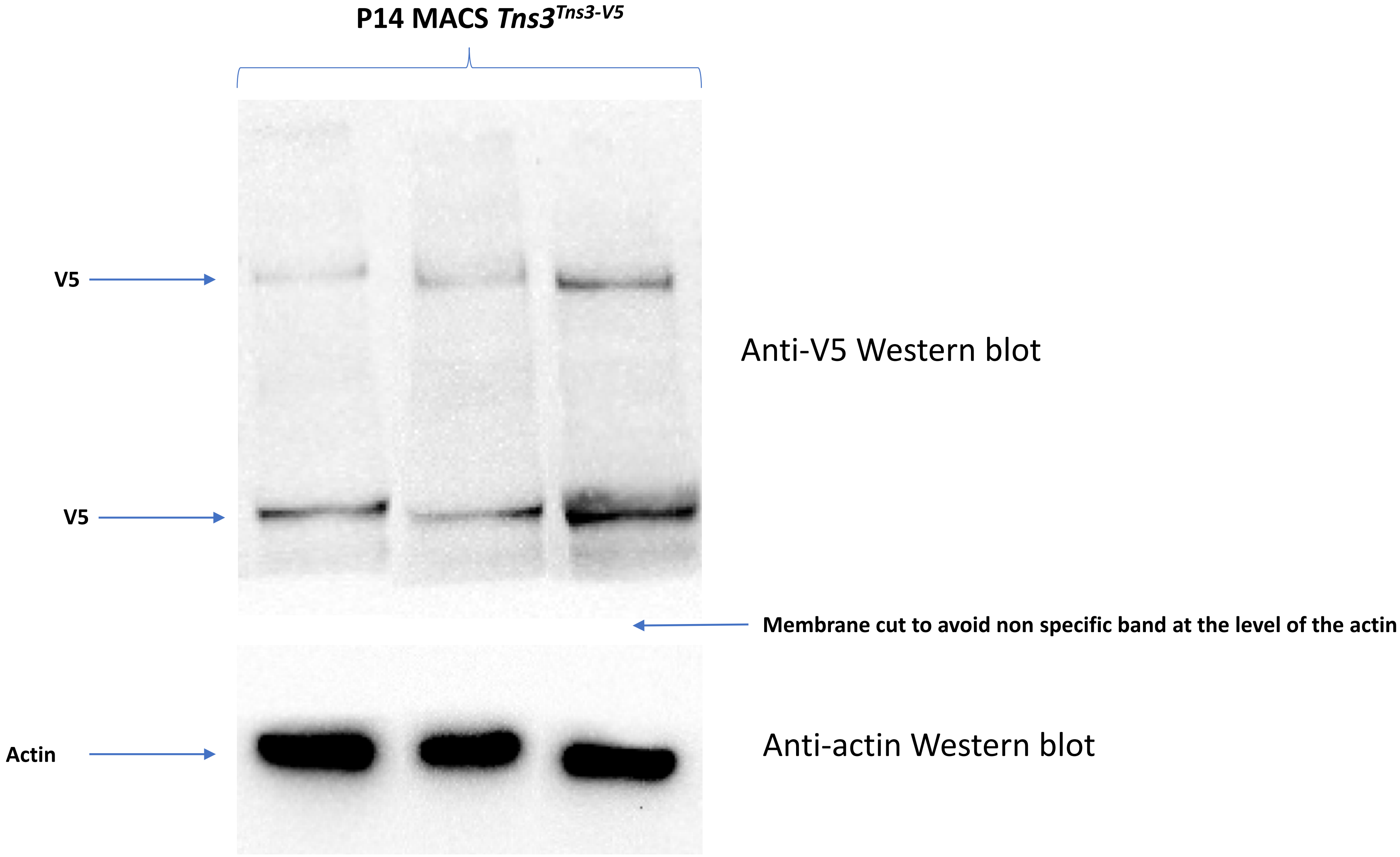
P14 MACS Tns3Tns3-V5
V5
V5
Actin
Anti-V5 Western blot
Membrane cut to avoid non specific band at the level of the actin
Anti-actin Western blot

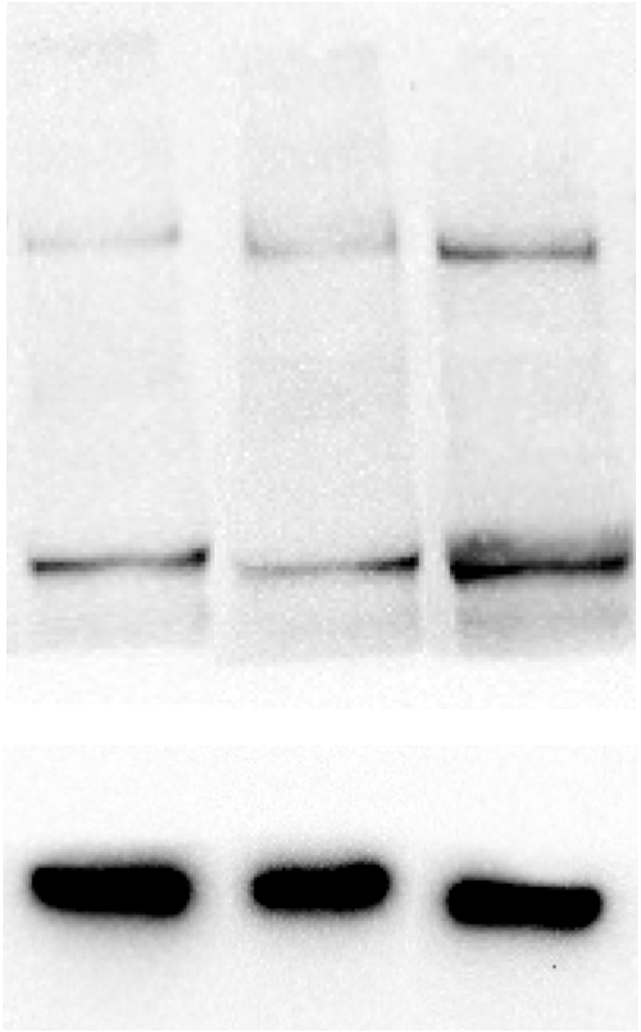

Supplement: Figure 2—figure supplement 2—source data 1. [file elife-80273-fig2-figsupp2-data1.zip › Figure 2ΓÇöfigure supplement 2ΓÇösource data 1/Figure_2-figure_supplement_2_source_data_1.pdf]
