# Supplementary figures and images for: Transient regulation of focal adhesion via Tensin3 is required for nascent oligodendrocyte differentiation
Source: eLife. 2022 Oct 10;11:e80273. doi: 10.7554/eLife.80273 (PMC9596163; doi:10.7554/eLife.80273)

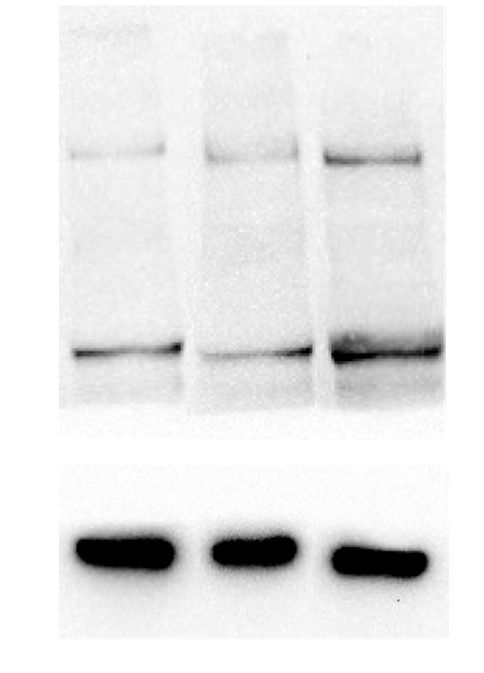

Supplement: Figure 2—figure supplement 2—source data 1. [file elife-80273-fig2-figsupp2-data1.zip › Figure 2ΓÇöfigure supplement 2ΓÇösource data 1/Western Blot_gel_P14.jpg]

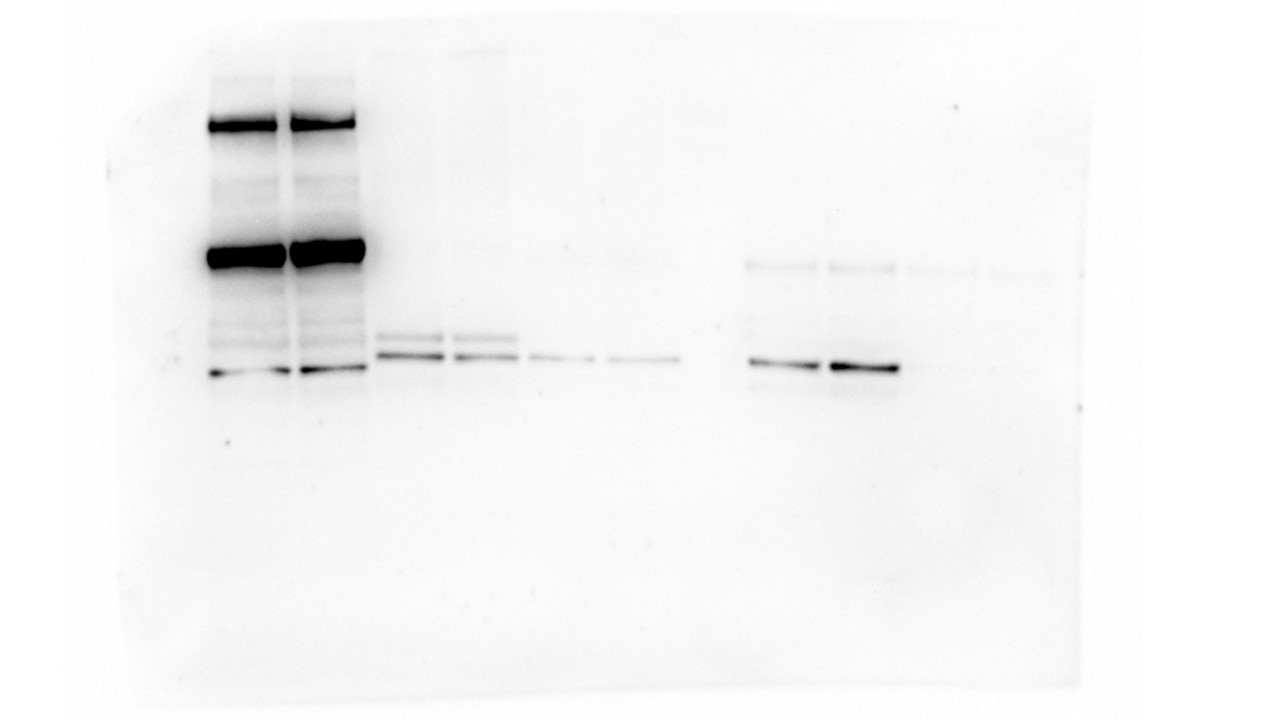

Supplement: Figure 2—figure supplement 2—source data 1. [file elife-80273-fig2-figsupp2-data1.zip › Figure 2ΓÇöfigure supplement 2ΓÇösource data 1/Western Blot_gel_P7_P21.jpg]

*Ctrl*   *Ctrl*            *gRNA#1*            *gRNA#2*   *ladder*

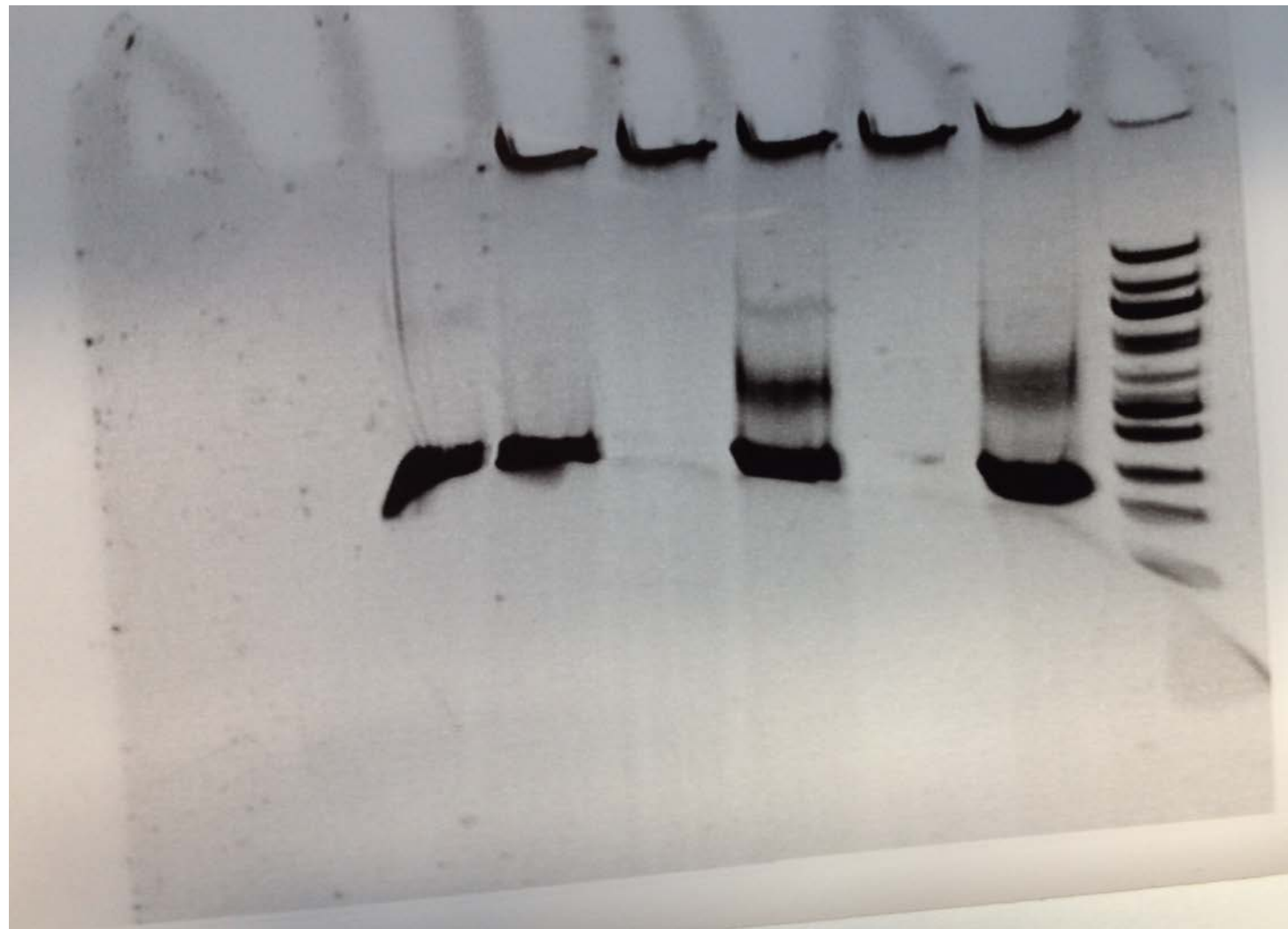

Supplement: Figure 3—figure supplement 3—source data 1. [file elife-80273-fig3-figsupp3-data1.zip › Figure_3-figure_supplement_3_source_data_1anotated.pdf]

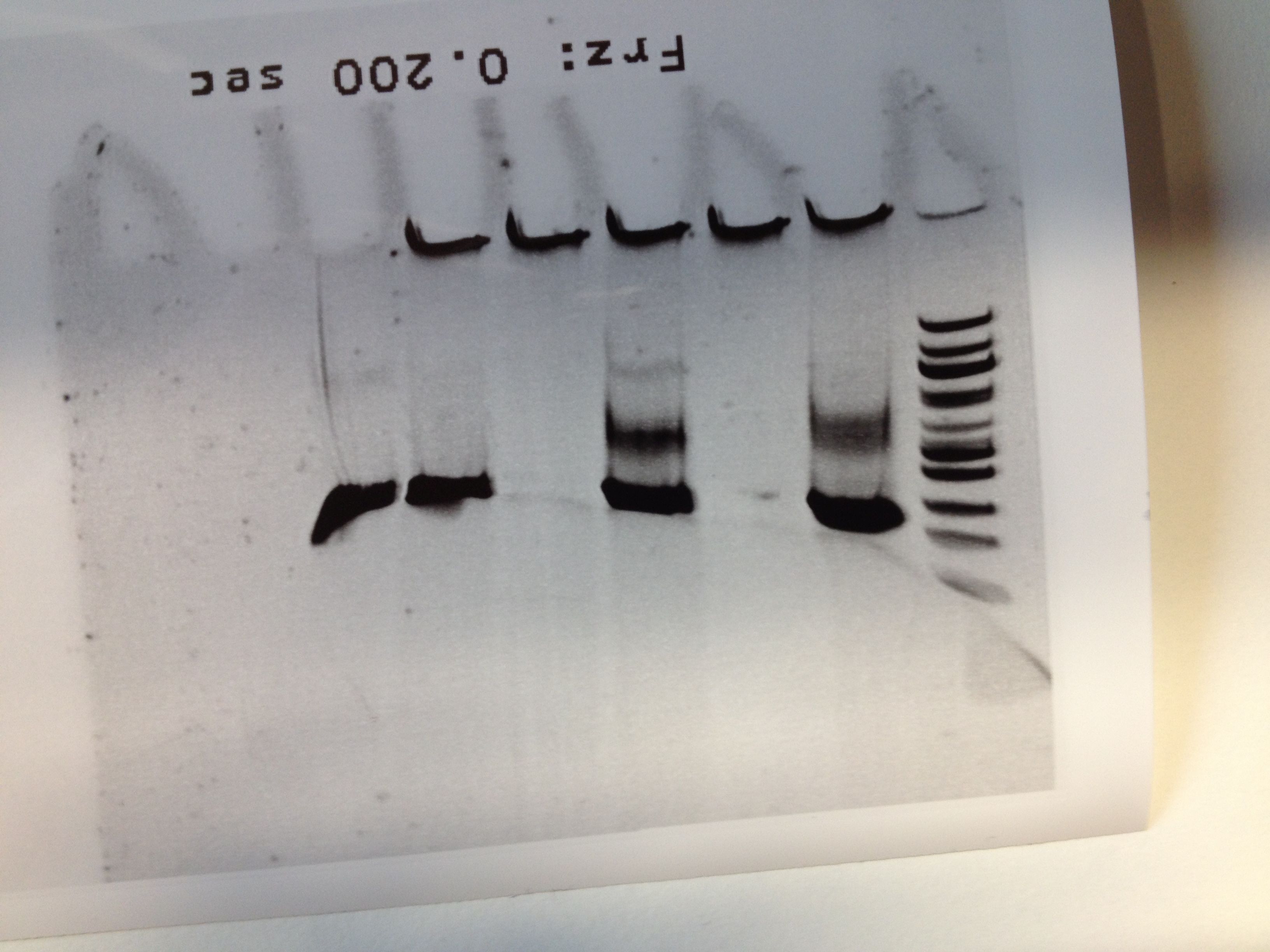

Supplement: Figure 3—figure supplement 3—source data 1. [file elife-80273-fig3-figsupp3-data1.zip › Figure_3-figure_supplement_3_source_data_1.JPG]
