# Supplementary material for: Transient regulation of focal adhesion via Tensin3 is required for nascent oligodendrocyte differentiation
Source: eLife. 2022 Oct 10;11:e80273. doi: 10.7554/eLife.80273 (PMC9596163; doi:10.7554/eLife.80273)
Supplement: Figure 3—source data 1. [file elife-80273-fig3-data1.pdf]

Subcloning of Tol2-pU6-gRNA pCAG-Cas9-2A-GFP integrative plasmid

Virtual cutting with Serial cloner> **pCAG is in the Tol2-mCherry plasmid**

Restriction analysis of **Tol2 mCherry**.xdna [Circular] Incubated with **AgeI + BsrGI**

1: **4 779 bp** - From BsrGI[5492] To AgeI[4779]

2: 713 bp - From AgeI[4779] To BsrGI[5492]

Restriction analysis of **PX458-Cas9-GFP**.xdna [Circular] Incubated with **AgeI + BsrGI + SspI**

1: **5 056 bp** - From AgeI[1239] To BsrGI[6295]

```
2:     2 939 bp   -   From SspI[7589]    To   AgeI[1239]

3:       908 bp   -   From BsrGI[6295]   To   SspI[7203]

4:       386 bp   -   From SspI[7203]    To   SspI[7589]
```

AgeI

5´...A▼CCGGT...3´
3´...TGGCC▲A...5´

Activity in NEBuffers
NEBuffer 1.1: 100%
NEBuffer 2.1: 75%
NEBuffer 3.1: 25%
CutSmart® Buffer: 75%

BsrGI

5´...T▼GTACA...3´
3´...ACATG▲T...5´

Activity in NEBuffers
NEBuffer 1.1: 25%
NEBuffer 2.1: 100%
NEBuffer 3.1: 100%
CutSmart® Buffer: 25%

SspI

5´...AAT▼ATT...3´
3´...TTA▲TAA...5´

Activity in NEBuffers

NEBuffer 1.1: 50%
NEBuffer 2.1: 100%
NEBuffer 3.1: 50%
CutSmart® Buffer: 50%

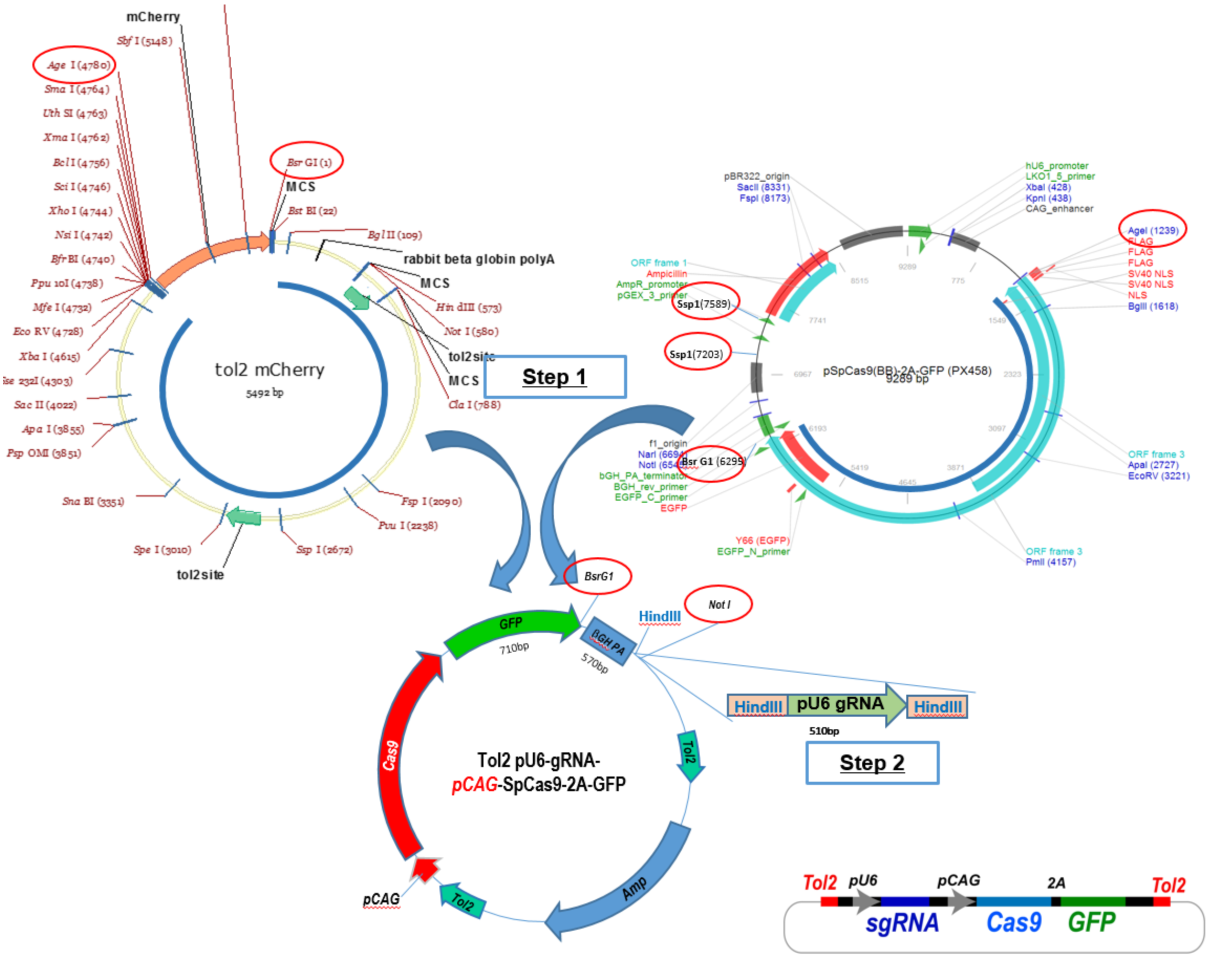

pU6 **BbsI/BbsI** gRNA

```
1     gagggcctat ttcccatgat tccttcatat ttgcatatac gatacaaggc 50
51    tgttagagag ataattggaa ttaatttgac tgtaaacaca aagatattag 100
101   tacaaaatac gtgacgtaga aagtaataat ttcttgggta gtttgcagtt 150
151   ttaaaattat gttttaaaat ggactatcat atgcttaccg taacttgaaa 200
201   gtatttcgat ttcttggctt tatatatctt GTGGAAAGGA CGAAACACCg 250
251   gGTCTTCgaG AAGACctgtt ttagagctaG AAAtagcaag ttaaaataag 300
301   gctagtccgt tatcaacttg aaaaagtggc accgagtcgg tgcTTTTTTg 350
351   ttttagagct agaaatagca agttaaaata aggctagtcc gtTTTTagcg 400
401   cgtgcgccaa ttctgcagac aaatggctct agaggtaccc gttacataac 450
451   ttacggtaaa tggcccgcct ggctgaccgc ccaacgaccc ccgcccattg 500
501   acgtcaatag
HindIIsite>A/AGCTT

            TTCGA/A
```

PCR product using the PX459 Cas9-gRNA plasmid

HindIII **PCR primers** **pU6** **BbsI/BbsI** gRNA

```
taataagcttgagggcctatttcccatgattccttcatatttgcatatacgatacaaggctgttagagagataat
tggaattaatttgactgtaaacacaaagatattagtacaaaatacgtgacgtagaaagtaataatttcttgggta
```

gtttgcagttttaaaattatgttttaaaatggactatcatatgcttaccgtaacttgaaagtatttcgatttctt
ggctttatatatcttGTGGAAAGGACGAAA**CACCggGTCTTCgaGAAGACct**gttttagagctaGAAAtagcaag
ttaaaataaggctagtccgttatcaacttgaaaaagtggcac**cgagtcggtgcTTTTTTgtt**aagcttatta

5’primer taataagctt**gagggcctatttcccatgat**

3’ primer taat**aagcttaacAAAAAAgcaccgactcg**

3’-5’ **cgagtcggtgcTTTTTTgtt**aagcttatta

PCR product **352** bp Tm- 57 C

Care: HindIII do not cut well with only 2 extra nt. Better 3-4.

Verifying the insertion of pU6-gRNA fragment

Cutting with **BstB1** (after GFP) and **Cla I** (after pU6-gRNA) > fragment of 1126 bp.

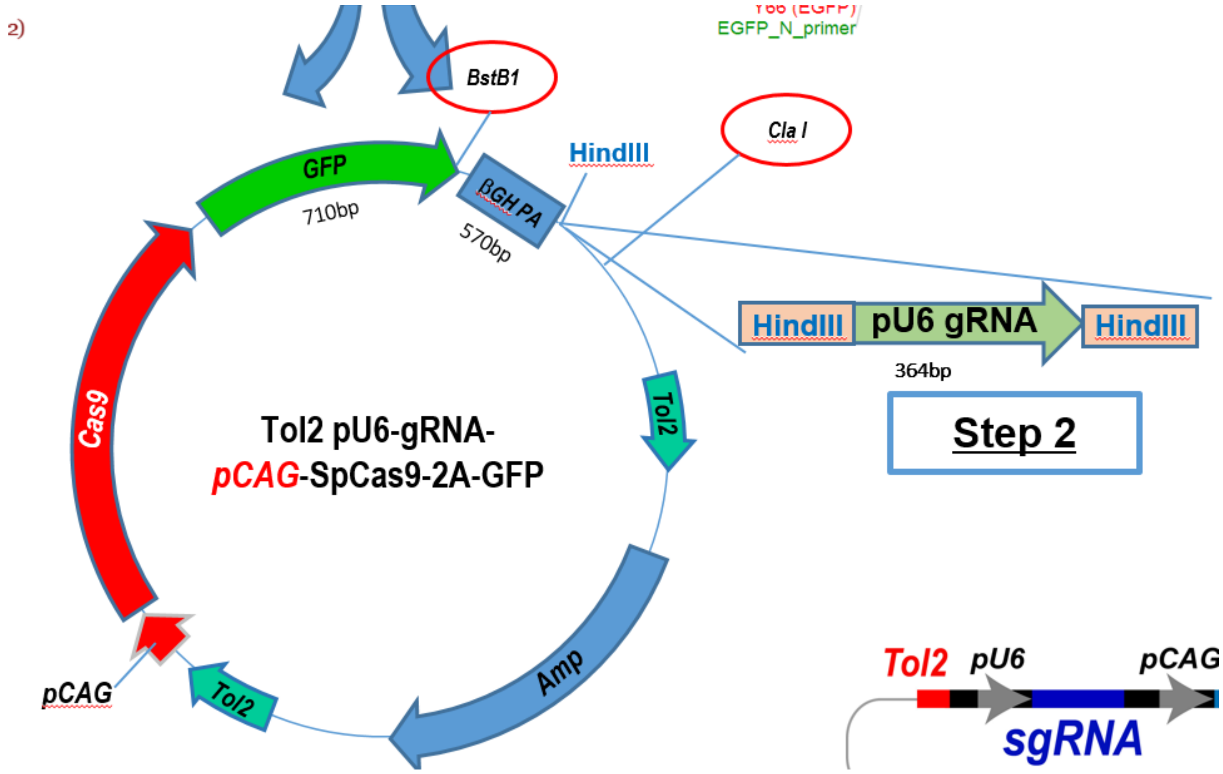

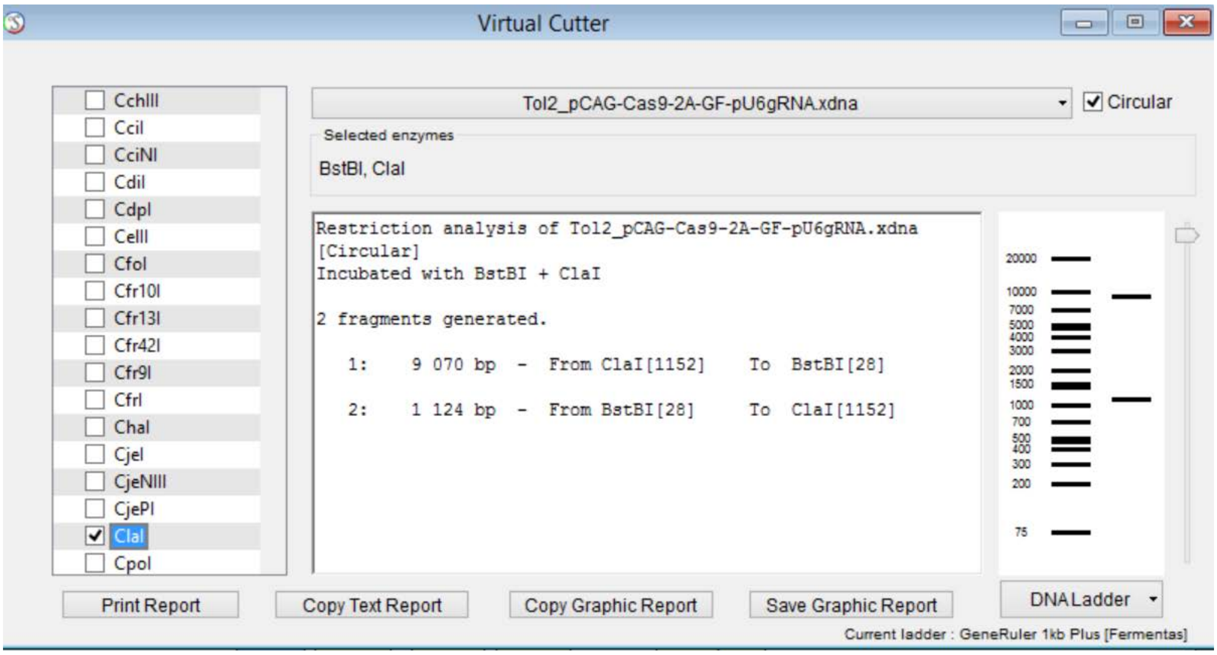

Cutting with **BsrG1** (end of GFP) and **NotI** (after pU6-gRNA) > fragment of 1 Kb.

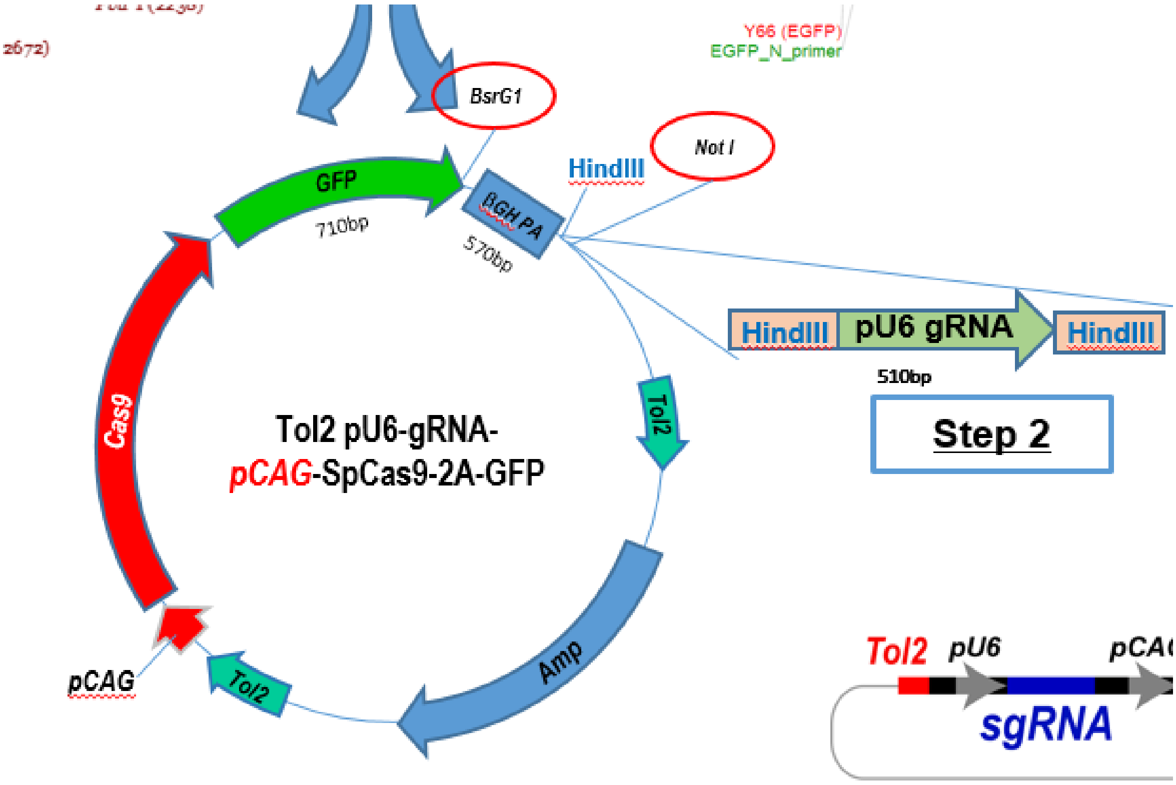

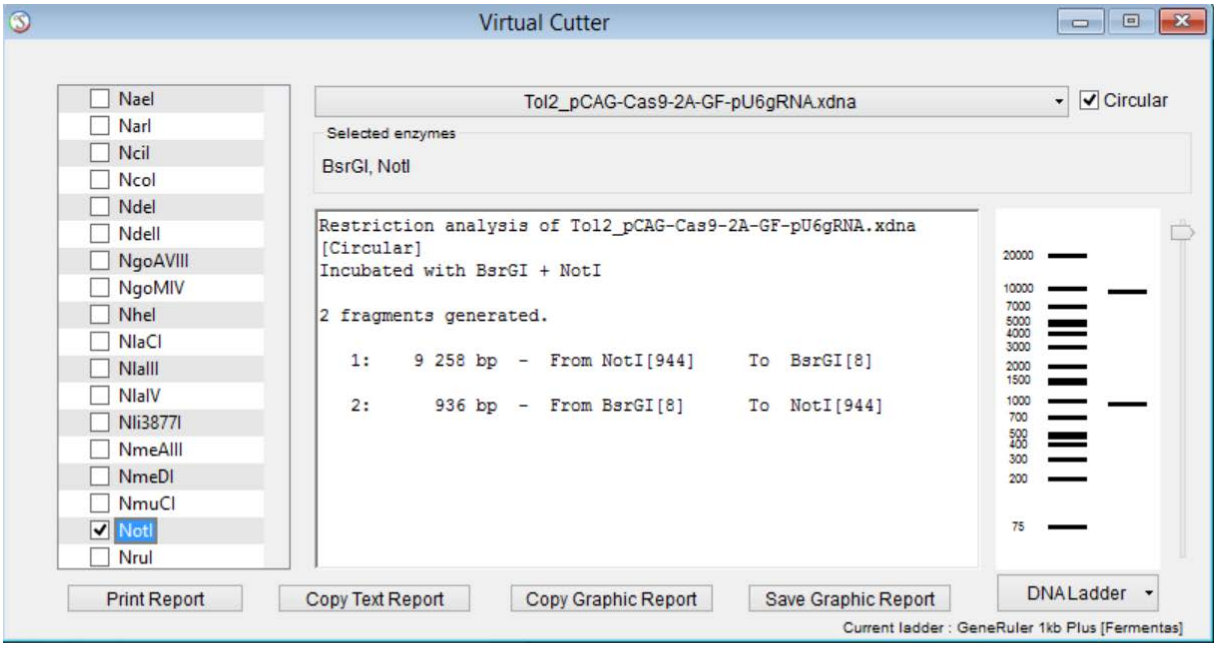
Virtual Cutter
NaeI
NarI
NciI
NcoI
NdeI
NdeII
NgoAVIII
NgoMIV
NheI
NlaCI
NlaIII
NlaIV
NIi3877I
NmeAIII
NmeDI
NmuCI
NotI
NruI
Tol2_pCAG-Cas9-2A-GF-pU6gRNA.xdna
Circular
Selected enzymes
BsrGI, NotI
Restriction analysis of Tol2_pCAG-Cas9-2A-GF-pU6gRNA.xdna [Circular]
Incubated with BsrGI + NotI
2 fragments generated.
1: 9 258 bp - From NotI[944] To BsrGI[8]
2: 936 bp - From BsrGI[8] To NotI[944]
20000
10000
7000
5000
4000
3000
2000
1500
1000
700
500
400
300
200
75
Print Report
Copy Text Report
Copy Graphic Report
Save Graphic Report
DNA Ladder
Current ladder : GeneRuler 1kb Plus [Fermentas]

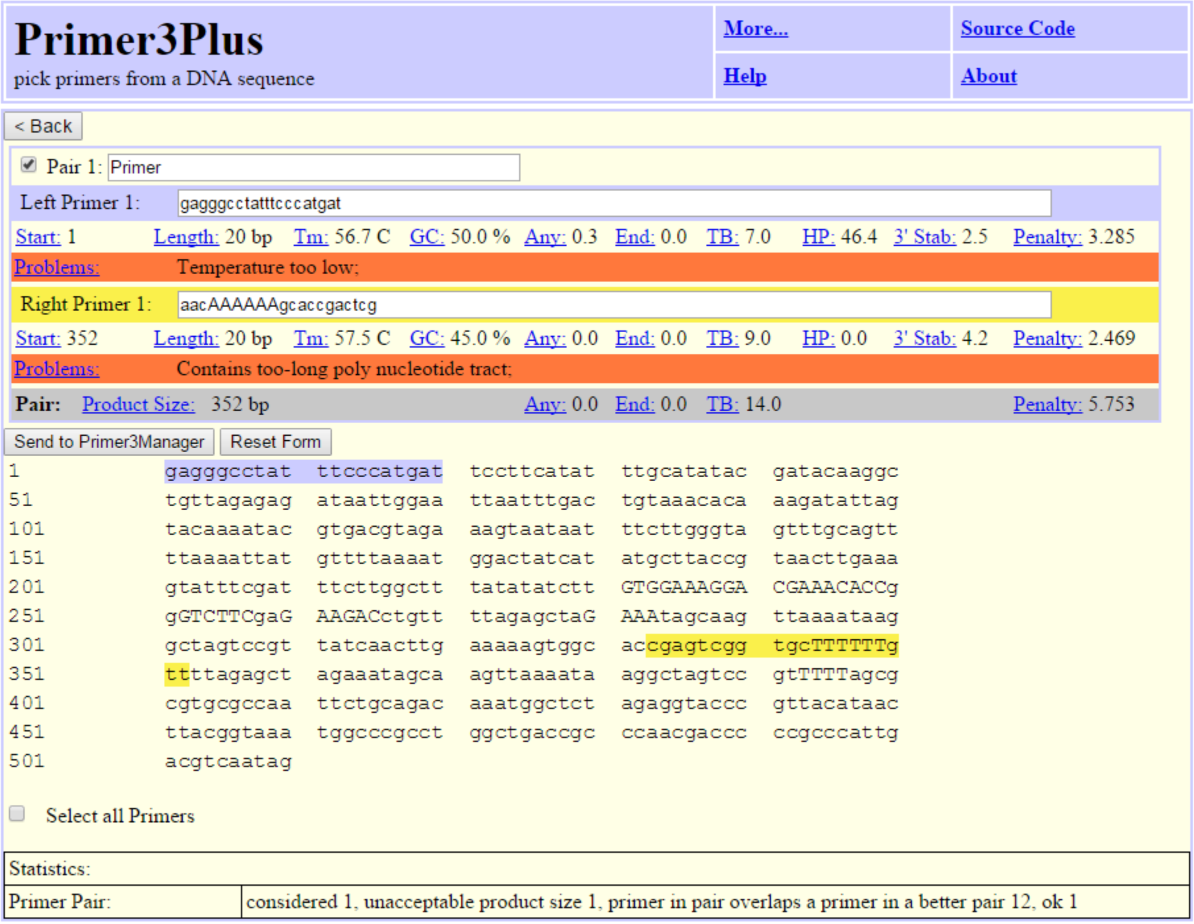
Primer3Plus
pick primers from a DNA sequence
More...
Source Code
Help
About
< Back
Pair 1: Primer
Left Primer 1: gagggcctatttcccatgat
Start: 1 Length: 20 bp Tm: 56.7 C GC: 50.0 % Any: 0.3 End: 0.0 TB: 7.0 HP: 46.4 3' Stab: 2.5 Penalty: 3.285
Problems: Temperature too low;
Right Primer 1: aacAAAAAAgcaccgactcg
Start: 352 Length: 20 bp Tm: 57.5 C GC: 45.0 % Any: 0.0 End: 0.0 TB: 9.0 HP: 0.0 3' Stab: 4.2 Penalty: 2.469
Problems: Contains too-long poly nucleotide tract;
Pair: Product Size: 352 bp Any: 0.0 End: 0.0 TB: 14.0 Penalty: 5.753
Send to Primer3Manager
Reset Form
1 gagggcctat ttcccatgat tccttcatat ttgcatatac gatacaaggc
51 tgttagagag ataattggaa ttaatttgac tgtaaacaca aagatattag
101 tacaaaatac gtgacgtaga aagtaataat ttcttgggta gtttgcagtt
151 ttaaaattat gttttaaaat ggactatcat atgcttaccg taacttgaaa
201 gtatttcgat ttcttggctt tatatatctt GTGGAAAGGA CGAAACACCg
251 gGTCTTCgaG AAGACCtgtt ttagagctaG AAAtagcaag ttaaaataag
301 gctagtccgt tatcaacttg aaaaagtggc accgagtcgg tgcTTTTTTg
351 ttttagagct agaaatagca agttaaaata aggctagtcc gtTTTTagcg
401 cgtgcgccaa ttctgcagac aaatggctct agaggtaccc gttacataac
451 ttacggtaaa tggcccgcct ggctgaccgc ccaacgaccc ccgcccattg
501 acgtcaatag
Select all Primers
Statistics:
Primer Pair: considered 1, unacceptable product size 1, primer in pair overlaps a primer in a better pair 12, ok 1
