# Supplementary material for: Transient regulation of focal adhesion via Tensin3 is required for nascent oligodendrocyte differentiation
Source: eLife. 2022 Oct 10;11:e80273. doi: 10.7554/eLife.80273 (PMC9596163; doi:10.7554/eLife.80273)
Supplement: Figure 3—figure supplement 3—source data 2. [file elife-80273-fig3-figsupp3-data2.pdf]

1. gRNAs targeting Tns3 ATG (5') with efficiently cutting and Indel generation

Mus musculus sequence found at chr11:8549019-8549117

Found 12 possible guide sequences in input (98 bp). Click on a PAM NGG match to show its guide sequence.
Shown below are the PAM site and the nucleotide at position -3 5' of it.
Colors green, yellow and red indicate high, medium and low specificity of the PAM's guide sequence in the genome.

```
0         10        20        30        40        50        60        70        80        90
ttcctcccttgcagGACCGTGGTTCTGAGTCCAGCCATGGAGGACAGCCACGAACTGGACCTTACGTATGTCACCGAGCGTATCATCGCAGTGTCCTT
  CCT..c        CCG..g      CCA..c           CCA..a      CCT..c        CCG..c
     CCC..g                     CCA..g           a..TGG
      CCT..c                    c..TGG
                                   t..AGG
```

	Position/ Strand	Guide Sequence + *PAM* Restriction Enzymes	Specificity Score	Efficacy Score	Prox. GC	Out-of-Frame Score	Off-targets for 0-1-2-3-4 mismatches + next to PAM	Genome Browser links to matches sort ☐ exons only ☐ chr11 only
gRNA#2	60 / rev	TACGCTCGGTGACATACGTA *AGG*<br>PCR primers<br>Restr. Enzymes: SnaBI	98	7	-	64 %	0 - 0 - 0 - 0 - 9<br>0 - 0 - 0 - 0 - 0<br>9 off-targets	4:intron:Dcaf17<br>4:intron:Ror2<br>4:intergenic:Ccdc13-Gm17163<br>show all...
gRNA#1	74 / rev	GGACACTGCGATGATACGCT *CGG*<br>PCR primers	97	68	-	69 %	0 - 0 - 0 - 2 - 34<br>0 - 0 - 0 - 0 - 0<br>36 off-targets	4:intergenic:Gm11381-Mylk4<br>4:intergenic:Gm4487-Gm24073<br>4:intron:Diap1<br>show all...
	48 / rev	CATACGTAAGGTCCAGTTCG *TGG*<br>PCR primers	95	19	-	68 %	0 - 0 - 0 - 1 - 24<br>0 - 0 - 0 - 0 - 0<br>25 off-targets	3:intergenic:Pik3r1-Gm25808<br>4:intergenic:Bche-Gm6098<br>4:intergenic:Gm22684-Anxa1<br>show all...
	56 / fw	CATGGAGGACAGCCACGAAC *TGG*<br>PCR primers	80	12	-	55 %	0 - 0 - 3 - 10 - 135<br>0 - 0 - 0 - 0 - 1<br>148 off-targets	2:intron:Colec10<br>2:intron:Zfp7<br>3:exon:Zfp217<br>show all...
	3 / rev	GAACCACGGTCCTGCAAGGG *AGG*<br>PCR primers	75	3	+	82 %	0 - 0 - 0 - 21 - 78<br>0 - 0 - 0 - 2 - 1<br>99 off-targets	3:intergenic:Dnm1-Ciz1<br>3:intergenic:Gm15336/Arhgap26-Gm15336<br>3:exon:Mmp25<br>show all...

gRNA #1

GGACACTGCGATGATACGCT RECOGNIZED SEQUENCE 2 (PAM on the - strand)
AGCGTATCATCGCAGTGTCC forward sequence corresponding (+ strand) **55% GC**

gRNA #2

TACGCTCGGTGACATACGTA RECOGNIZED SEQUENCE 1 (PAM on the - strand)
TACGTATGTCACCGAGCGTA forward sequence corresponding (+ strand) **50% GC**

gRNA #1 (25+25pb)

Forward 5'-3' CACCGGACACTGCGATGATACGCT

Reverse 3'-5' CCTGTGACGCTACTATGCGACAAA

Reverse 5'-3' AAACAGCGTATCATCGCAGTGTCC

gRNA #2 (25+25pb)

Forward 5'-3' CACCGTACGCTCGGTGACATACGTA

Reverse 3'-5' CATGCGAGCCACTGTATGCATCAAA

Reverse 5'-3' AAACTACGTATGTCACCGAGCGTAC

Genomic sequence and gRNA for Tns3

gacaggcgaggtggccttcagctcagtctacatgcagatcctttgggtggctgctgttcccagctttctccgaagccct
cattaggtgagtgttcatcctttttcttgttatccttttcttgacctcctcttcctcccttgcagGACCGTGGTTCTGA
GTCCAGCC**ATG**GAGGACAG*CCA*CGAACTGGACCTTACGTATGTCACCG**AGCGTATCATCGCAGTGTCC**TTCCCTGCCAG
TTGTTCAGAGGAGTCCTACTTGCACAGCCTGCAGGAAGTCACACGCATGCTCAAGTGTAAGCATGGAGACAACTACCTG
gtgagtggggatgatagccacggtgtggtggatgggctggaggcaggagtccctcagaggactcgtgtgagggccaggg
gtgtccctgtgccttgcttgcttacacctgaggaatggtttgggggcctggaatctgactcatgagtctaggggccatg
tcctctacccttctattgttagctttgctgttgttgtgtcacgggctgtgacagttgactgtaggggtggctgctgcct

---

New Primers for PCR
CCT PAM in reverse strand
CCCTxxxX gRNA#1
TCACCGXX gRNA#2

---

2. gRNAs targeting Tns3 TGA (3') with efficiently cutting and Indel generation

Tns3 last part of the cDNA region containing the stop codon.

```
4301 CCCTGAGTAT GACCTTGGTT CAAGAACCTC CTCCAGTGTC TACAGTGGTA
4351 CACTTCAAGG TGTCAGCCCA GGGCATCACC CTGACTGACA ATCAGAGAAA
4401 GCTCTTCTTC CGGAGGCATT ACCCGGTCAG CAGTGTGATT TTCTGTGCTT
4451 TGGATCCACA AGACAGGAAG TGGATCAAAG ATGGCCCTTC TTCTAAAGTC
4501 TTTGGATTTG TGGCCCGGAA ACAGGGCAGT GCTACAGACA ACGTATGCCA
4551 CCTGTTTGCA GAGCATGACC CCGAGCAACC TGCCAGTGCC ATTGTCAACT
4601 TTGTGTCAAA GGTCATGATT GGCTCCCCTA AGAAGATCTG Agcccttcct
4651 agccaaacct gccagatgcc tcatagggcc ctcaacatgg cagtggggtg
4701 aggcagggcc cccattcgta ccagattgac aatcttcaca ttccagactc
4751 aacaagagga gaagaatatg acaactcttc aaaccaagaa caaacaatgt
4801 caccactgac tggccttaaa gaagctgctc ttccgacaca cctatatagt
4851 caggcgcatt ttgagtggat ggatcttggc ccgggaaatg cctcatcagg
4901 gccccaagga caagggctgt acaaggaggg agcatggaag acaaggctgt
```

Query sequence for CRISPOR:

TTGTGTCAAAGGTCATGATTGGCTCCCCTAAGAAGATCTGAgcccttcctagccaaacctgccagatgcctcatagggccctcaacatggcagtggggtga

TCATGATTGGCTCCCCT sequence also present in chr12:100150650-100150682> avoid to use it in the gRNA

1. **Guide sequence: GGCCCTATGAGGCATCTGGC AGG**
2. **Guide sequence: TATGAGGCATCTGGCAGGTT TGG**
3. **Guide sequence: TCTGGCAGGTTTGGCTAGGA AGG > more efficiently cutting gRNA**

TTTGTGTCAAAGGTCATGATTGGCTCCCCTAAGAAGATCTGAgccct tcctagccaaacctgccagatgcctcatagggccctcaacatggcagtggggtgaggcag
ggcccccattcgtaccagattgacaatcttcacattccagactcaacaagaggagaagaatatgacaactcttcaaaccaagaacaaacaatgtcaccactgactgg
ccttaaagaagctgctcttccgacacacctatatagtcaggcgcattttgagtggatggatcttggcccgggaaatgcctcatcagggccccaaggacaagggctgt
acaaggagggagcatggaagacaaggctgt

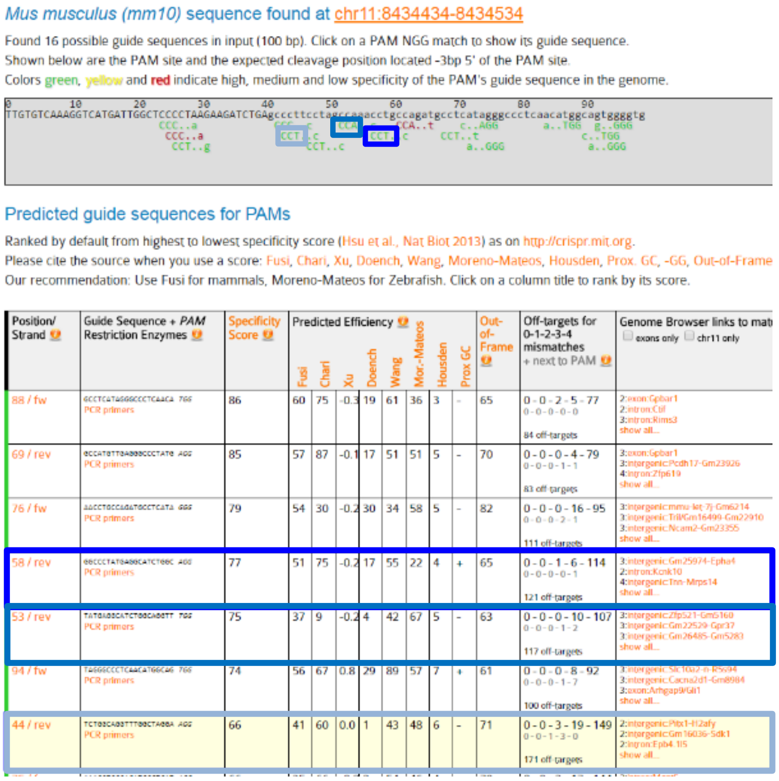

Mus musculus (mm10) sequence found at chr11:8434434-8434534

Found 16 possible guide sequences in input (100 bp). Click on a PAM NGG match to show its guide sequence.
Shown below are the PAM site and the expected cleavage position located -3bp 5' of the PAM site.
Colors green, yellow and red indicate high, medium and low specificity of the PAM's guide sequence in the genome.

```
0         10        20        30        40        50        60        70        80        90
TTGTGTCAAAGGTCATGATTGGCTCCCCTAAGAAGATCTGAgcccttcctagccaaacctgccagatgcctcatagggccctcaacatggcagtggggtg
```

Predicted guide sequences for PAMs

Ranked by default from highest to lowest specificity score (Hsu et al., Nat Biot 2013) as on http://crispr.mit.org.
Please cite the source when you use a score: Fusi, Chari, Xu, Doench, Wang, Moreno-Mateos, Housden, Prox. GC, -GG, Out-of-Frame
Our recommendation: Use Fusi for mammals, Moreno-Mateos for Zebrafish. Click on a column title to rank by its score.

Position/ Strand	Guide Sequence + *PAM* Restriction Enzymes	Specificity Score	Predicted Efficiency Fusi	Chari	Xu	Doench	Wang	Mor.-Mateos	Housden	Prox GC	Out-of-Frame	Off-targets for 0-1-2-3-4 mismatches + next to PAM	Genome Browser links to mat[illegible] exons only chr11 only
88 / fw	GCCTCATAGGGCCCTCAACA *TGG* PCR primers	86	60	75	-0.3	19	61	36	3	-	65	0 - 0 - 2 - 5 - 77<br>0 - 0 - 0 - 0 - 0<br>84 off-targets	2:exon:Gpbar1<br>2:intron:Ctif<br>3:intron:Rims3<br>show all...
69 / rev	GCCATGTTGAGGGCCCTATG *AGG* PCR primers	85	57	87	-0.1	17	51	51	5	-	70	0 - 0 - 0 - 4 - 79<br>0 - 0 - 0 - 1 - 1<br>83 off-targets	3:exon:Gpbar1<br>3:intergenic:Pcdh17-Gm23926<br>4:intron:Zfp619<br>show all...
76 / fw	AACCTGCCAGATGCCTCATA *GGG* PCR primers	79	54	30	-0.2	30	34	58	5	-	82	0 - 0 - 0 - 16 - 95<br>0 - 0 - 0 - 2 - 1<br>111 off-targets	3:intergenic:mmu-let-7j-Gm6214<br>3:intergenic:Trill/Gm16499-Gm22910<br>3:intergenic:Ncam2-Gm23355<br>show all...
58 / rev	GGCCCTATGAGGCATCTGGC *AGG* PCR primers	77	51	75	-0.2	17	55	22	4	+	65	0 - 0 - 1 - 6 - 114<br>0 - 0 - 0 - 0 - 1<br>121 off-targets	3:intergenic:Gm25974-Epha4<br>2:intron:Kcnk10<br>4:intergenic:Tnn-Mrps14<br>show all...
53 / rev	TATGAGGCATCTGGCAGGTT *TGG* PCR primers	75	37	9	-0.2	4	42	67	5	-	63	0 - 0 - 0 - 10 - 107<br>0 - 0 - 0 - 1 - 2<br>117 off-targets	3:intergenic:Zfp521-Gm5160<br>3:intergenic:Gm22529-Gpr37<br>3:intergenic:Gm26485-Gm5283<br>show all...
94 / fw	TAGGGCCCTCAACATGGCAG *TGG* PCR primers	74	56	67	0.8	29	89	57	7	+	61	0 - 0 - 0 - 8 - 92<br>0 - 0 - 0 - 1 - 7<br>100 off-targets	3:intergenic:Slc10a2-n-R5s94<br>3:intergenic:Cacna2d1-Gm8984<br>3:exon:Arhgap9/Gli1<br>show all...
44 / rev	TCTGGCAGGTTTGGCTAGGA *AGG* PCR primers	66	41	60	0.0	1	43	48	6	-	71	0 - 0 - 3 - 19 - 149<br>0 - 0 - 1 - 3 - 0<br>171 off-targets	2:intergenic:Pitx1-H2afy<br>2:intergenic:Gm16036-Sdk1<br>2:intron:Epb4.1l5<br>show all...

3.Guide sequence: TCTGGCAGGTTTGGCTAGGA AGG **> best gRNA cutting efficiency**

validation Primers

guideRna44revLeft	AGCCTTGTCTTCCATGCTCC	Tm 60.035
guideRna44revRight	TGCTTGGCACATCGTAACCT	Tm 59.964

Genome fragment with validation primers and guide sequence

Genomic sequence chr11:8434468-8434491 including primers, forward strand:

AGCCTTGTCTTCCATGCTCC CTCCTTGTACAGCCCTTGTCCTTGGGGCCCTGATGAGGCATTTCCCGGGCCAAGATCCATCCACTCAAAATGCGCCTGACTATATAGGTGTGTCGGAAGAGCAGCTTCTTTAAGGCCAGTCAGTGGTGACATTGTTTGTTCTTGGTTTGAAGAGTTGTCATATTCTTCTCCTCTTGTTGAGTCTGGAATGTGAAGATTGTCAATCTGGTACGAATGGGGGCCCTGCCTCACCCCACTGCCATGTTGAGGGCCCTATGAGGCA**TCTGGCAGGTTTGGCTAGGAAGG**GCTCAGATCTTCTTAGGGGAGCCAATCATGACCTTTGACACAAAGTTGACAATGGCACTGGCAGGTTGCTCGGGGTCATGCTCTGCAAACAGGTGGCATACGTTGTCTGTAGCACTGCCCTGTTTCCGGGCCACAAATCCAAAGACTCTGCAAGAGACAAGTCTAGTCAGAGGAGCCAGTACCGTGTGCAGCAAGCACCATGCATGCATGCAGGCTTTCTCTTCCTCATGATAGCAACCTGGGAACAGTACAGACTGCCCCTGC *AGGTTACGATGTGCCAAGCA*

Sequence length: 588

Method: Primer3.2 with default settings, target length 500-700 bp

---

Expression of guide RNA

Summary of all primers explained below

guideRna44revT7sense	TAGG**TCTGGCAGGTTTGGCTAGGA**
guideRna44revT7antisense	AAAC**TCCTAGCCAAACCTGCCAGA**
guideRNA44revT7PromSense	TAATACGACTCACTATAGG**TCTGGCAGGTTTGGCTAGGA**GTTTTAGAGCTAGAAATAGCAAG
guideRNAallT7PromAntisense	AAAAGCACCGACTCGGTGCCACTTTTTCAAGTTGATAACGGACTAGCCTTATTTTAACTTGCTATTTCTAGCTCTAAAAC
guideRNA44revU6sense	ACACCG**TCTGGCAGGTTTGGCTAGGA**G
guideRNA44revU6antisense	AAAAC**TCCTAGCCAAACCTGCCAGA**CG
